# Pangenome Analysis of the Plant Pathogen *Pseudomonas syringae* Reveals Unique Natural Products for Niche Adaptation

**DOI:** 10.1002/anie.202503679

**Published:** 2025-05-02

**Authors:** Shuaibing Zhang, Ying Huang, Raed Nachawati, Philipp Huber, Grit Walther, Lucas Gregor, Ivan Vilotijević, Pierre Stallforth

**Affiliations:** ^1^ Department of Paleobiotechnology Leibniz Institute for Natural Product Research and Infection Biology – Hans Knöll Institute Beutenbergstraße 11a D‐07745 Jena Germany; ^2^ National Reference Center for Invasive Fungal Infections Leibniz Institute for Natural Product Research and Infection Biology – Hans Knöll Institute Beutenbergstraße 11a D‐07745 Jena Germany; ^3^ Institute of Organic Chemistry and Macromolecular Chemistry Friedrich Schiller University Jena Humboldtstraße 10 D‐07743 Jena Germany; ^4^ Cluster of Excellence Balance of the Microverse Friedrich Schiller University Jena Fürstengraben 1 D‐07743 Jena Germany

**Keywords:** Bioactivity, Halogenase, Natural products, *P. syringae*, Pangenome analysis

## Abstract

*Pseudomonas syringae* is a soil‐dwelling bacterium that exhibits remarkable niche adaptability, and it is known for its devastating impact as a plant pathogen. This bacterium has an outstanding capability to produce a wide array of biologically active natural products. *P. syringae* coexists with amoebal predators and fungal strains, which drives the production of secondary metabolites for predator evasion in addition to niche adaptation. In this study, we conducted a broad pangenomic analysis of 18 taxonomically distinct *P. syringae* strains, leading to the identification of 231 biosynthetic gene clusters (BGCs). Among these, nonribosomal peptide synthetases (NRPSs) were particularly abundant, indicating their potential significance within this ecological context. We discovered and elucidated the structures of two novel classes of bioactive compounds, the syrilipamides and chlorosecimides. Furthermore, a bioinformatic analysis enabled the identification of an undescribed halogenase, SecA, essential for the chlorination of secimide A. We observed that syrilipamides and secimides and in particular mixtures thereof, exhibit amoebicidal activities. Additionally, secimides showed selective antifungal activity. These findings provide valuable insights into the ecological roles of *P. syringae* natural products and highlight their potential for biotechnological and therapeutic applications.

## Graphical abstract

A pangenome analysis of the plant pathogen *Pseudomonas syringae* reveals two novel natural product families that facilitate niche adaptation. Comprehensive genomic and chemical analyses uncover unique biosynthetic gene clusters linked to host specificity and environmental fitness.

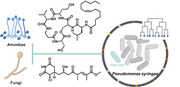

## Introduction

Bacteria of the species *Pseudomonas syringae* are prevalent and ubiquitous plant pathogens^[^ ^1^, ^2^ ^]^ known for their rich secondary metabolism.^[^ ^3^, ^4^ ^]^ The genome of this soil‐dwelling bacterium harbors an extensive number of biosynthetic gene clusters (BGCs) that encode enzymes responsible for the biosynthesis of structurally and functionally diverse natural products. These compounds include biosurfactants,^[^ ^5^ ^]^ siderophores,^[^ ^6^ ^]^ toxins,^[^ ^7^, ^8^ ^]^ and signaling molecules,^[^ ^9^ ^]^ which collectively facilitate the bacterium′s ability to adapt to diverse habitats and to infect a multitude of different plant hosts.^[^ ^10^, ^11^ ^]^ Whereas some of these metabolites display a broad spectrum of activities, many of them have highly specialized functions, which are shaped by specific evolutionary selection pressures. Consequently, much of the structural diversity remains undiscovered as they elude detection by conventional screens testing for generic antibiotic, antifungal, or anticancer activity. Our previous research demonstrated that many natural products produced by *Pseudomonas* species provide protection from bacterivorous predators such as amoebae or nematodes.^[^ ^12^, ^13^, ^14^, ^15^, ^16^, ^17^ ^]^

In this study, we surveyed 18 taxonomically distinct *P. syringae* strains that represent major subgroups of the species.^[^ ^18^ ^]^ As illustrated in the phylogenetic analysis (Figure S1), these strains capture an important portion of the genetic diversity within *P. syringae*. By combining pangenomic in silico analyses with an ecology‐driven functional investigation of the secondary metabolome, we discovered two families of novel natural products exhibiting toxicity against an amoebal predator and a fungal competitor. We provide a comprehensive characterization of the biological activity of these compounds and an initial investigation into their biosynthesis, which is crucial for elucidating how nature generates structural diversity and functional specificity. Moreover, the integration of biosynthetic and bioactivity studies allows the anticipation of natural products that remain elusive under standard laboratory culture conditions. Identifying key enzymes involved in these biosynthetic processes opens new avenues for genetic and metabolic engineering, potentially enhancing the production of these compounds or creating novel derivatives with improved properties.

## Results and Discussion

### Atlas of the Biosynthetic Gene Clusters (BGCs) in *P. syringae*

Eighteen taxonomically diverse *P. syringae* genomes were analyzed for the presence of natural product BGCs using antiSMASH 7.0.^[^ ^19^ ^]^ A total of 231 BGCs were identified and classified into five categories—nonribosomal peptide synthetases (NRPS), polyketide synthases (PKS), PKS/NRPS hybrids, biosynthetic machinery for terpenes, and ribosomally synthesized and post‐translationally modified peptides (RiPPs)—with an average of 13 BGCs per strain (Table S19), which is about 2–8 times higher than the average BGC count in other *Enterobacteria*.^[^ ^20^ ^]^ NRPSs represent the most prevalent BGC class in *P. syringae*, accounting for 48% of the total BGCs, with approximately six BGCs per strain. The abundance of NRPS BGCs suggests that their corresponding natural products may play important ecological roles, which potentially contribute to the pathogen's competitive adaptation to natural environments and its ability to evade predators. *Trans*‐acyltransferase type I polyketides (*trans*‐AT type I PKSs), responsible for producing secimide,^[^ ^21^ ^]^ and PKS/NRPS hybrid classes, such as syringolin BGC,^[^ ^22^ ^]^ are enriched and widely distributed. These compounds are hypothesized to play dual roles in plant pathogenesis and microbial competition, underscoring their relevance to *P. syringae*’s lifestyle.^[^ ^23^, ^24^ ^]^ In contrast, terpene and RiPP BGCs were found to be rather underrepresented, with only carotenoid^[^ ^25^ ^]^ and klebsazolicin^[^ ^26^ ^]^ BGCs identified in two or three selected *P. syringae* genomes, respectively. To explore the relationship between BGC diversity and host physiology, we examined the distribution of BGCs in relation to the pathovars’ environmental niches. Notably, NRPS clusters producing syringomycin‐like compounds were enriched in strains such as DSM 1241 (isolated from a pear tree), DSM 1242 (isolated from a lemon tree), and CECT 7752 (isolated from a mango tree). Woody hosts impose strong selection pressures on bacteria due to the potential production of antimicrobial compounds, robust physical barriers, and diverse microbial communities.^[^ ^27^, ^28^, ^29^ ^]^ The production of NRPS‐derived metabolites likely provides a competitive advantage in these environments by facilitating host colonization and microbial competition. Conversely, strains (e.g., SZ57, isolated from soil)^[^ ^16^ ^]^ infecting non‐woody hosts appear to harbor fewer NRPS and PKS BGCs since these environments impose other selective pressures for the production of complex secondary metabolites. This likely reflects a trade‐off between the ecological demands of the host environment and the energetic cost of maintaining large, multifunctional biosynthetic pathways.

### Pangenome Analysis of *P. syringae*

**Figure 1:**
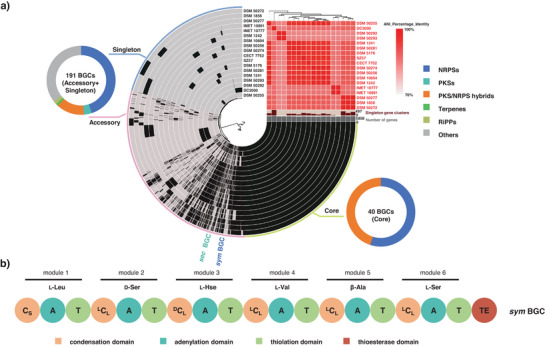
Pangenome analysis of 18 *P. syringae* strains. a) The central component of the interface portrays a dendrogram derived from hierarchical clustering, which is based on the presence/absence of genes. In the upper right corner, the red chart illustrates the average nucleotide identity (ANI) among these strains. Within the circular interface, each concentric layer shows the complete gene repertoire of a single genome. The interface is divided into three sections, the core region (light green), which contains genes that are ubiquitous across all 18 *P. syringae* genomes; the accessory region (pink), which contains genes shared by a subset of *P. syringae* genomes, the *sym* and *sec* BGCs are located in this region, and the singleton region (light blue), which contains species‐specific genes exclusive to a single genome. The total number of BGCs for the core, accessory, or singleton group is visible within the BGC donut charts as well as the proportion of each BGC class. b) Domain organization of the *sym* BGC, based on predictions from antiSMASH and NaPDoS. The predicted A‐domain substrates and C‐domain specificities are indicated. C_S_: C_starter_ domain; ^L^C_L_: C domains that condense two L‐amino acids; ^D^C_L_: C domain that condenses an L‐ and a D‐amino acid.

In order to identify previously undescribed bacterial natural products that are shared by several but not all phylogenetically distinct *P. syringae* strains, the genomes of 18 strains (Figure S1) were subjected to a pangenomic analysis.^[^ ^30^ ^]^ We thus classified BGCs into core, accessory, or singleton genes. Core genes, shared across all strains, are essential for fundamental biological processes. In contrast, accessory and singleton genes, present in some or unique to individual strains, respectively, often provide specific ecological adaptive advantages.^[^ ^31^ ^]^ As a result, a pangenomic analysis enabled the prediction of specific functions of the products of a BGC according to which group it belongs to. By performing BGC annotations within the pangenome, we systematically analyzed the distribution of genes involved in natural product biosynthesis across core, accessory, and singleton regions (Figure 1a). Among the core genes, we identified a BGC required for the production of a siderophore, Pf‐5 pyoverdine,^[^ ^32^ ^]^ which is crucial in iron acquisition, competition, and plant interactions.^[^ ^33^ ^]^ We also found a putative redox‐cofactor BGC with low similarity to the lankacidin BGC (the corresponding biosynthetic proteins share 13% sequence similarity). In the accessory genes group, we identified PKS/NRPS hybrid gene cluster, such as a syringolin BGC,^[^ ^24^ ^]^ *trans*‐AT type I PKS, a secimide BGC,^[^ ^21^ ^]^ and NRPS‐like BGC required for the generation of syringafactin^[^ ^34^, ^35^ ^]^ and syringomycin.^[^ ^36^ ^]^ COG20 (Clusters of Orthologous Groups, 2020 update) categories are a curated set of functional annotations based on orthologous relationships among genes. In this study, we used these categories to filter and identify NRPS genes by focusing on annotations associated with secondary metabolism pathways, using the anvi'o platform to examine accessory and singleton genes in the pangenome.^[^ ^37^ ^]^ This led to the identification of 13 NRPS genes (five in the accessory and eight in the singleton category, Figure S2, Table S20). Following identification, these NRPS genes were extracted, annotated, and localized within their respective genomes to identify NRPS modules from complete BGCs. The complete BGCs were found to encompass not only those enabling the biosynthesis of well‐characterized molecules such as syringafactin and syringomycin but also a previously uncharacterized BGC of NRPSs containing six distinct modules. Each module comprises condensation (C), adenylation (A), and thiolation (T) domains. Additionally, the C_starter_ domain suggested the incorporation of an N‐terminal fatty acid moiety.^[^ ^38^ ^]^ Taken together, this NRPS may produce a lipopeptide consisting of six amino acids (Figure 1b, Figure S3). Overall, this BGC is found in six out of the 18 *P. syringae* strains (Figure S4).

### Identification, Isolation, and Structure Elucidation of Syrilipamides

**Figure 2:**
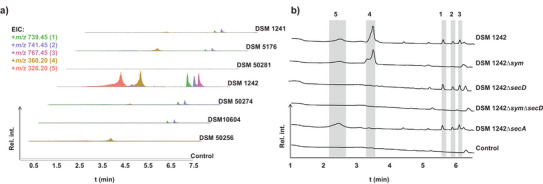
Metabolite analysis of *P. syringae* strains. a) Extracted ion chromatograms (EIC) of culture extracts from various *P. syringae* strains, demonstrating that DSM 1242 was the most prolific producer of both syrilipamides (**1**–**3**) and secimides (**4**–**5**). b) HPLC profile of crude extracts from DSM 1242 wild type and gene deletion mutants, showing the absence of syrilipamides in the DSM 1242∆*sym* mutant, the absence of secimides in the DSM 1242∆*secD* mutant, and the absence of both molecules in the double deletion mutant. Additionally, secimide B (**4**) is absent in the DSM 1242∆*secA* mutant, which shows an accumulation of secimide A (**5**) (detected at λ = 190 nm). A control using SM/5 medium extract is included for comparison.

**Figure 3:**
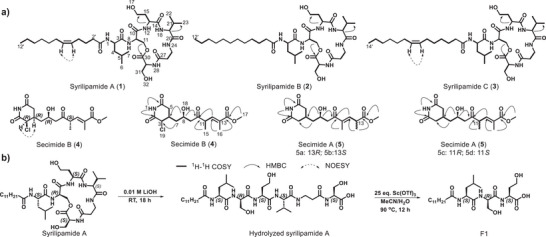
Structures of syrilipamides, secimides, and scandium(III) triflate‐mediated peptide hydrolysis product of syrilipamide A. a) Chemical structures of syrilipamides A–C (**1**–**3**) and secimides A (**5a**–**5d**) and B (**4**). Key ^1^H–^1^H, COSY, HMBC, and NOESY correlations are indicated. b) Scandium triflate‐mediated peptide cleavage yields fragment F1, which contains only D‐serine.

To identify the natural products associated with the unique NRPS BGC shown in Figure 1b, we initially cultured all six *P. syringae* strains containing this BGC in different liquid media (Figure S5). Analysis of bacterial culture extracts using SM/5 medium showed that only *P. syringae* DSM 1242 produced five distinct compounds. Three of these, with pseudomolecular masses of *m/z* 739.45, 741.45, and 767.45, appeared to be consistent with lipopeptide structures containing six amino acids (Figure 2a, Figures S6). The underlying reason why DSM 1242 is a prolific producer of natural products remains unclear; however, it appears that this strain harbors more regulatory genes in the vicinity of the respective BGC than the other strains (Figure S7). Next, we inactivated the respective BGC by generating an in‐frame deletion of the gene fragment coding for the C_starter_ domain (Δ*sym*). Comparison of HPLC profiles of culture extracts from DSM 1242 and the deletion mutant showed that the three peaks present in the wild type were absent in the deletion mutant, suggesting that these peaks could be attributed to the products of this NRPS BGC (Figure 2b, Figure S6). In order to isolate sufficient quantities of these natural products, the high‐producing strain DSM 1242 was cultured on an 8‐L scale in SM/5 medium. Extraction of the supernatant with ethyl acetate and chromatographic purification of the extract led to the isolation of three related compounds, which we named syrilipamides (*sym*). High‐resolution mass spectrometry (HRMS) measurements revealed pseudomolecular ion peaks with *m/z* 739.4574 [M + H]^+^ for syrilipamide A (**1**), *m/z* 741.4727 [M + H]^+^ for syrilipamide B (**2**), and *m/z* 767.4886 [M + H]^+^ for syrilipamide C (**3**), which are consistent with the molecular formulae C_36_H_62_N_10_O_6_, C_36_H_64_N_10_O_6_, and C_38_H_66_N_10_O_6_, respectively (Figure 3a).

To fully elucidate the structure of the syrilipamides, we began with an in silico prediction of the specificities of every A domain of the corresponding  NRPS. This provided insight into the amino acid sequence of the syrilipamides. The presence of a C_starter_ domain suggested the presence of an N‐terminal fatty acid moiety,^[^ ^38^ ^]^ which was consistent with nuclear magnetic resonance (NMR), tandem mass spectrometry (MS^2^), and chemical degradation studies. Additionally, NMR spectroscopy revealed that all three compounds contain a fatty acid moiety with chain lengths of C12 (compounds **1** and **2**) or C14 (compound **3**), featuring a double bond at C‐5′/C‐6′ (**1**) or C‐7′/C‐8′ (**3**). Derivatization of the hydrolyzed amino acids with Marfey's reagent confirmed the presence of 1 × L‐Ser, 1 × D‐Ser, 1 × L‐Leu, 1 × L‐Val, 1 × L‐Hse, and 1× β‐Ala. However, these combined analyses did not allow us to determine the specific locations of L‐ and D‐serine (Figure 3a, Figures S8–S10, Tables S1–S3).

Scandium(III) triflate‐mediated peptide hydrolysis^[^ ^12^, ^39^ ^]^ preferentially cleaves amide bonds adjacent to serine or threonine residues. Using this method, we obtained a shorter fragment of syrilipamide (F1), which was fully hydrolyzed and derivatized using Marfey's reagent. Analysis of F1 containing 1 × L‐leucine, 1 × D‐serine, and 1 × L‐homoserine, allowed us to assign the D‐configuration of serine at position 2 and L‐configuration of serine at position 6 (Figure 3b, Figure S11, Table S4).

The predicted molecular mass for a linear peptide, based on the amino acids and the fatty acid identified by MS^2^ experiments, differed by 18 mass units from the observed mass, suggesting that syrilipamide may be a macrocycle. Further analysis of NMR data showed an HMBC correlation between the hydrogens H‐11 (**1**: *δ*_H_ 4.42; **2**: *δ*_H_ 4.43; **3**: *δ*_H_ 4.43) and C‐30 (**1**: *δ*_C_ 170.04; **2**: *δ*_C_ 170.01; **3**: *δ*_C_ 170.01), indicating that syrilipamides are cyclic lipopeptides with macrolactones formed by esterification between the hydroxyl group of serine 2 and the C‐terminus of serine 6. Interestingly, the syrilipamides do not fall in any of the six established groups^[^ ^40^ ^]^ of *Pseudomonas*‐derived cyclic lipopeptides.^[^ ^41^, ^42^ ^]^ Instead, they form a new group characterized by six amino acids, including an unusual β‐alanine and a fatty acid moiety that lacks the 3‐OH group (Figure 3a, Table S5).

### Phylogenetic Analysis of the C_starter_ Domain and the A Domain of the β‐Alanine‐Incorporating Module

Since the syrilipamides differ from typical *P. syringae*‐derived lipopeptides due to an unusual fatty acid and amino acid moiety, we investigated the evolutionary origins of the corresponding biosynthetic genes. To this end, we performed phylogenetic analyses on the coding regions of the A and C domains utilizing Maximum Likelihood (ML) estimation. We anticipated that the domains would cluster based on their substrate specificity or their functional categories. Specifically, the C_starter_ domain and the A domain that selects β‐alanine are widely distributed within the *P. syringae* group, exhibiting amino acid identities that range from 50% to 100% (Figure S12). Phylogenetic analyses revealed close relationships between these domains and homologs in *Burkholderia* and *Xenorhabdus*, suggesting a potential horizontal gene transfer (HGT) event, as these genera share habitats with *P. syringae*. Additionally, we conducted a genome‐wide HGT analysis of strain DSM 1242 using the HGTector tool,^[^ ^43^ ^]^ which identified 569 genes with plausible HGT origins. However, the syrilipamide BGC was not among them (Table S28). A phylogenetic analysis of all C domains from the NaPDoS database^[^ ^44^ ^]^ indicated a close relationship between the C_starter_ domain of syrilipamide and that of syringomycin, also produced by *P. syringae* (Figure S12). This finding is unexpected, as syringomycin incorporates a fatty acid chain featuring a 3‐OH group. Collectively, these results suggest that the syrilipamide BGC likely arose through genetic recombination^[^ ^45^ ^]^ rather than HGT.

### Identification, Isolation, and Structure Elucidation of Secimides

We used the anvi'o platform to identify PKS genes within the accessory and singleton groups, resulting in the discovery of 13 PKS genes, of which six are in the accessory and seven in the singleton pangenome (Figure S2, Table S21). These PKS genes were extracted, annotated, and mapped within the genomes to distinguish between isolated genes coding for PKS modules and complete BGCs. Among the BGCs identified were those encoding for known NRPS/PKS hybrids, generating syringolin, and *trans*‐AT PKS secimide, the latter of which was present in seven out of the 18 *P. syringae* strains. We were particularly interested in the *trans*‐AT PKS products due to their remarkable structural diversity and bioactivity profiles.^[^ ^46^, ^47^, ^48^ ^]^ Subsequent metabolite analysis of seven *P. syringae* strains harboring this BGC revealed that only the DSM 1242 strain produced two prominent natural products (Figure 2a, Figures S5 and S6), with pseudomolecular ion peaks at *m/z* 360.1198 [M + H]^+^ and 326.1585 [M + H]^+^, corresponding to the molecular formulae C_16_H_22_O_6_NCl and C_16_H_23_O_6_N, respectively. The compound with the molecular formula C_16_H_23_O_6_N was shown to be secimide A,^[^ ^21^ ^]^ a known product of *trans*‐AT type I PKS, while the other was identified as undescribed chlorosecimide A. Deletion of the AT domain (Δ*secD*) abolished the production of both molecules, showing the link between the identified *trans*‐AT PKS BGC and the biosynthetic genes (Figure 2b, Figure S6). Following the purification of these compounds using HPLC, we could link a major isomer, secimide B (**4**), to the peak at *m/z* 360.1198. In contrast, the peak with *m/z* 326.1585 corresponded to secimide A (**5a**–**5d**) as a mixture of four isomers in a 1:1:1:1 ratio (Figure 3a).

To elucidate the connectivity of secimide B (**4**), 1D and 2D NMR analyses were performed. The ^1^H NMR (500 MHz, DMSO‐*d*_6_) spectrum shows the presence of three methyl groups at *δ*_H_ 1.11 (d, *J* = 7.06 Hz, H‐15), *δ*_H_ 1.87 (d, *J* = 1.25 Hz, H‐16), and *δ*_H_ 3.68 (s, H‐17), along with an olefinic proton signal at *δ*_H_ 6.57 (dd, *J* = 10.1, 1.25 Hz, H‐12). Analysis of the ^13^C NMR and HSQC spectral data revealed 16 carbon signals, encompassing three methyl (one oxygenated), three methylene, five methine (one olefinic, one oxygenated, one chlorinated, and two hydrogenated), and five carbons without hydrogens (one olefinic and four carbonyls). Further investigation via ^1^H‐^1^H COSY correlations unveiled two spin coupling systems: H‐15 (*δ*_H_ 1.11) – H‐11 (*δ*_H_ 3.65) – H‐12 (*δ*_H_ 6.57) and H‐9 (*δ*_H_ 2.57) – H‐8 (*δ*_H_ 4.00) – H‐7 (*δ*_H_ 1.53) – H‐4 (*δ*_H_ 2.71) – H‐3(*δ*_H_ 4.73) / H5 (*δ*_H_ 2.51). Additionally, HMBC cross‐peaks from NH‐1 (*δ*_H_ 11.17) to C‐2 (*δ*_C_ 168.92) and C‐6 (*δ*_C_ 171.81); from H‐4 (*δ*_H_ 2.71) to C‐2 (*δ*_C_ 168.92), C‐6 (*δ*_C_ 171.81), C‐5 (*δ*_C_ 58.06), C‐8 (*δ*_C_ 63.49); from H‐8 (*δ*_H_ 4.00) to C‐9 (*δ*_C_ 48.91) and C‐10 (*δ*_C_ 208.50); from H‐12 (*δ*_H_ 6.57) to C‐10 (*δ*_C_ 208.50), C‐15 (*δ*_C_ 15.71), C‐13 (*δ*_C_ 128.61), C‐16 (*δ*_C_ 12.71), and C‐14 (*δ*_C_ 167.38); from H‐17 (*δ*_H_ 3.68) to C‐14 (*δ*_C_ 167.38) facilitated the establishment of its connectivity, showing that secimide B bears a 3‐chlorine substitution compared to **5c/d**.

To fully elucidate the absolute configuration of secimide B, a combination of extensive NMR analysis and computational predictions was performed. The relative configuration of secimide B was determined by NMR chemical shift calculations for eight diastereomers (Figure S13), and DP4 + analyses gave a 98.24% probability of **4d** (3R*,4R*,8R*,11S*). Additionally, ROESY data further supported the relative configurations of 3R*,4R*, evidenced by the correlation between H‐4 (*δ*_H_ 2.71) and H‐3 (*δ*_H_ 4.73). The absolute configuration of **4** was determined to be 3*R*,4*R*,8*R*,11*S* (Figure 3a) based on the calculated ECD spectrum, which closely matched the experimental ECD data of secimide B (Figures S14 and S15, Tables S6–S8). As for the isomer mixture of **5a**–**5d**, individual isomers were separated using HPLC on a C18 column (Figure S16). However, subsequent NMR analysis indicated that the isomers had reverted to a mixture (Figure S17). The structural differences between **5a** and **5b**, as well as **5c** and **5d**, were traced back to differences in the configuration of stereogenic centers at C‐13 and C‐11, respectively. Specifically, **5a** and **5b** have a double bond between C‐11/C‐12, while **5c** and **5d** have a double bond between C‐12/C‐13. Notably, the double bond can readily isomerize between these positions, facilitating interconversion between the four isomers (Figure 3a, Table S8). We hypothesized that four interconverting isomers originate from a single diastereomer that is generated by the *sec* biosynthetic machinery. To identify the parental isomer, we cultured the strain at 5° C, followed by freeze‐drying and extraction at low temperature. Notably, under these conditions, isomer **5d** was identified as the predominant isomer of secimide A in the extracts (Figure S16). Although only partial structural data (¹H and HSQC) were obtained before its conversion into the other isomers, density functional theory (DFT) calculations assigned its structure with an 8*R*,11*S* configuration (Table S9).

### Identification of the Enzyme That Chlorinates Secimide A

**Figure 4:**
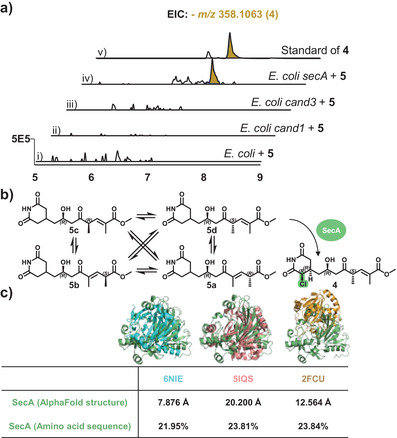
Heterologous expression and structural analysis of halogenase candidates. a) Heterologous expression of *cand1*, *cand3*, and *secA*, demonstrating the conversion of secimide A (**5**) to secimide B (**4**) by SecA. b) SecA‐catalyzed chlorination of interconverting secimides **5a**–**d**. c) AlphaFold model of SecA aligned with known Fe(II)/α‐KG‐dependent halogenases identified based on a FoldSeek analysis. Three crystal structures of the structurally most similar halogenases—BesD (PDB: 6NIE), WelO5 (PDB: 5IQS), and SyrB2 (PDB: 2FCU)—are shown. They exhibit low structural similarity and sequence identity to SecA.

As secimide B is an unknown 3‐chloro congener of secimide A, we investigated the underlying chlorination mechanism. A close inspection of the *trans*‐AT PKS BGC did not reveal any candidate genes encoding a halogenase enzyme. This suggests that secimide A is chlorinated post‐assembly via a halogenase that may not be encoded within the *sec* BGC. Using genes of all four known halogenase groups (Fe(II)/α‐KG‐dependent, flavin‐dependent, SAM‐dependent halogenase, and haloperoxidase)^[^ ^49^, ^50^ ^]^ as queries for a basic local alignment search tool (BLAST) search,^[^ ^51^ ^]^ we identified four candidate halogenase genes in the genome of *P. syringae* DSM 1242 (*cand1*–*cand4*) belonging to three distinct groups (Table S10). *Cand1*, *cand3*, and *cand4* are annotated to encode Fe(II)/α‐KG‐dependent halogenases, while *cand2* is annotated as flavin‐dependent halogenase gene^[^ ^49^, ^50^, ^52^ ^]^ based on their PFAM analysis (Table S10). *Cand1*, *cand3*, and *cand4* were the most likely candidates; however, mutants Δ*cand1*, Δ*cand2*, Δ*cand3*, Δ*cand4*, Δ*cand3*Δ*cand4*, and Δ*cand1*Δ*cand3*Δ*cand4* were still able to produce chlorinated secimide B (Figure S6). Taking into account that Fe(II)/α‐KG‐dependent halogenases are members of the oxygenase family and highly similar to related enzymes that catalyze hydroxylation reactions,^[^ ^53^ ^]^ a gene, *secA* (*cand5*), located near the secimide BGC and annotated as coding for a non‐heme Fe(II)/α‐KG‐dependent 2,4‐dichlorophenoxyacetate dioxygenase,^[^ ^54^ ^]^ caught our attention. Additionally, phylogenetic analyses comparing this enzyme with α‐KG‐dependent enzymes revealed that SecA differs from known halogenases of this family (Figure S18). Indeed, the Δ*secA* mutant was not able to produce secimide B but accumulated secimide A (Figure 2b). Subsequent heterologous expression of *cand1*, *cand3*, and *secA* in *Escherichia coli* showed that only the strain containing *secA* was able to convert secimide A to secimide B (Figure 4a). Therefore, the results confirmed that although SecA clusters phylogenetically with dioxygenases (Figure S19), it acts as a halogenase, enabling the chlorination of secimide A in the final step of the biosynthesis (Figure 4b). Furthermore, phylogenetic analysis indicates that SecA is widely distributed within the *P. syringae* group, exhibiting high sequence identities ranging from 90% to 100%. In contrast, SecA shows significantly lower identity (below 45%) in other bacterial groups (Figure S20).

To investigate the structural similarity of SecA to known halogenases, we conducted a FoldSeek search.^[^ ^55^ ^]^ Candidate halogenases identified through this search were aligned to an AlphaFold^[^ ^56^, ^57^, ^58^ ^]^ model of SecA using PyMOL.^[^ ^59^ ^]^ The SecA model incorporated Fe^2^⁺ and Cl⁻ ions to mimic the enzyme's active state, facilitating a detailed comparison of structural features relevant to its function. Interestingly, the closest structural homolog identified was BesD (PDB: 6NIE),^[^ ^60^ ^]^ an Fe(II)/α‐KG‐dependent chlorinase, albeit with a remarkably low structural similarity (RMSD = 7.876 Å) and a low sequence identity (21.95%), which was surprising given the anticipated conservation within this enzyme family (Figure 4c, Figure S20).

Additional comparisons with other Fe(II)/α‐KG‐dependent chlorinases, such as WelO5 (PDB: 5IQS)^[^ ^61^ ^]^ and SyrB2 (PDB: 2FCU),^[^ ^62^ ^]^ revealed an even greater structural divergence, with RMSD values of 20.200 and 12.564 Å, respectively. These results suggest that SecA is structurally distinct from these well‐characterized halogenases. Despite these differences, analysis of the facial triad sequences (Figure S18) indicates that SecA contains an HXG/A motif, a hallmark of Fe(II)/α‐KG‐dependent halogenases. In contrast, other enzymes within the same phylogenetic clade exhibit an HXD/E motif, characteristic of hydroxylases. Therefore, although SecA's structural uniqueness may hint at a novel subclass within the Fe(II)/α‐KG‐dependent halogenase family, its functional characteristics and the evolutionary conservation of the HXG/A motif support its classification within this enzyme family.^[^ ^63^, ^64^ ^]^

### Substrate Specificity Analysis of SecA

Secimide **5** occurs as a mixture of four isomers, which interconvert at room temperature. Interestingly, secimide B is predominantly present as a single isomer, and more stable at room temperature than secimide A. We sought to understand the selectivity of the SecA enzyme. The most parsimonious explanation is that only isomer **5d** is the substrate of SecA. In order to understand this selectivity, we investigated the mechanism of chlorination by SecA using an in silico approach. SecA was initially annotated as a dioxygenase and it is structurally different from known halogenases. We used the FoldSeek search server to identify similar crystal structures containing an iron complex. The crystal structure of alkyl sulfatase AtsK (PDB: 1OIK)^[^ ^65^ ^]^ met our threshold criteria for similarity. Structural alignment of SecA with AtsK revealed a low RMSD of 1.045 Å (AF3 model) and 1.199 Å (AF2 model), indicating a high degree of structural similarity between the two enzymes (Figure S21).

Following the structural alignment with AtsK, as well as the HXG motif in SecA, we identified potential catalytic sites within SecA by comparison. Given the high local structural similarity between the two enzymes, we hypothesized that SecA also harbors a 2‐oxoglutaric acid molecule at the same locus as AtsK. To explore the interaction potential of the four secimide A isomers (**5a**–**5d**) within the catalytic pocket of SecA, we assessed the proximity of each isomer's site of halogenation to the putative iron ion. The four isomers were first optimized using DFT at the B3LYP/6–31G(d) level^[^ ^66^ ^]^ and subsequently docked into the AlphaFold2‐model of SecA.^[^ ^56^ ^]^ Docking results revealed that only isomer **5d** penetrated deeper into the catalytic pocket compared to the other isomers (Figure S21), with respective distances from the active site to the iron ion of 4.4, 3.8, 4.2, and 3.2 Å (Figure S21). This spatial proximity post‐chlorination suggests that (8*R*,11*S*)‐**5** (**5d**) is the most likely substrate to undergo catalytic chlorination by SecA, as opposed to the other isomers. This is in agreement with the fact that **5d** is the initial biosynthetic product of the secimide BGC. These findings suggest that the position of the double bond and the configuration of the methyl groups at positions 13 and 15 play critical roles in determining whether the substrate can successfully access the catalytic site of SecA.^[^ ^56^ ^]^ This structural specificity likely underlies the enzyme's selective catalytic efficiency toward particular substrates. Although our in silico approaches provide insights into the selective chlorination of isomer **5d** by SecA, these findings still remain speculative. Experimental validation, e.g., based on a crystal structure of SecA, is necessary to confirm the precise positioning of the ion within the active site and to fully elucidate the molecular basis of its substrate specificity.

### Biological Activity and Synergy of Syrilipamides and Secimides

Given that *P. syringae* and social amoebae share the same ecological niche,^[^ ^67^ ^]^ we assessed the susceptibility of the 18 *P. syringae* strains to amoebal predation using a plaque assay. To this end, vegetative *Dictyostelium discoideum* AX2 cells were placed onto solid medium containing a lawn of each respective bacterial strain. The formation of a grazing plaque, which eventually develops into amoebal fruiting bodies, indicates that the amoebae can feed on the bacteria. In contrast, the absence of grazing plaques or fruiting bodies indicates bacterial resistance to amoebal predation.^[^ ^16^ ^]^ Among the 18 *P. syringae* strains tested, only *P. syringae* DSM 1242 exhibited resistance to amoebal predation (Figure S22). Considering that the production of amoebicidal secondary metabolites enable bacteria to efficiently kill amoebal predators, we proceeded to evaluate the toxicity of the syrilipamides and secimides against amoebae. This ecological function of the bacterial secondary metabolites appeared particularly relevant since only *P. syringae* DSM 1242 was able to produce both the syrilipamides and secimides in substantial quantities. Syrilipamides A–C exhibited potent amoebicidal activity, with IC_50_ (*D. discoideum*) values of 13.5, 5.5, and 2.1 µg mL^−1^, respectively. In contrast, secimide A did not show any amoebicidal activity, while secimide B demonstrated moderate toxicity, with an IC_50_ (*D. discoideum*) value of 49.2 µg mL^−1^. We proceeded to test combinations of compounds mirroring the production ratios observed in SM/5 media (Figure S23). A mixture of syrilipamides A–C (1:1:1, wt/wt/wt) exhibited an IC_50_ (*D. discoideum*) value of 1.1 µg mL^−1^. A mixture of secimides A (**5a–d**) and B (4:1, wt/wt) displayed increased toxicity compared to the individual components with a combined IC_50_ (*D. discoideum*) value of 27.1 µg mL^−1^. A combination of syrilipamides A–C, secimide A, and secimide B (1:1:1:1:4, wt/wt/wt/wt/wt) resulted in total IC_50_ (*D. discoideum*) of 5.5 µg mL^−1^ (Table S11). These findings demonstrate the strong bioactivity of syrilipamides, comparable to other potent CLPs such as jessenipeptin (4 µg mL⁻¹),^[^ ^12^ ^]^ nunapeptin B (11.5 µg mL⁻¹),^[^ ^15^ ^]^ and surpassing the activity of anikasin (94 µg mL⁻¹).^[^ ^13^ ^]^ This suggests that syrilipamides are highly bioactive secondary metabolites, potentially providing *P. syringae* with a competitive ecological advantage by enabling efficient predation defense or interactions within its environmental niche. To evaluate the edibility of the mutant strains by *D. discoideum*, we conducted grazing assays using Δ*sym*, Δ*secD*, and Δ*symΔsecD* strains. None of these strains were susceptible to grazing by *D. discoideum*, indicating that they are inedible to this organism. These findings suggest that additional secondary metabolites produced by DSM 1242 may act as virulence factors, further contributing to its defense against predation. Additionally, both secimides A and B demonstrated selective activity against the fungal strain *Sporobolomyces salmonicolor* (MIC = 25 µg mL^−1^, Tables S12–S14).^[^ ^68^ ^]^ These findings further highlight the important biological roles of bacterially produced cyclic lipopeptides and *trans*‐AT PKS polyketides,^[^ ^47^, ^69^ ^]^ particularly in the context of microbial interactions.^[^ ^12^, ^13^, ^15^, ^17^, ^70^, ^71^ ^]^ Importantly, the modification of secimide A by a halogenase alters its biological function.^[^ ^72^, ^73^, ^74^, ^75^ ^]^

## Conclusion

*P. syringae's* ability to thrive in its ecological niche relies on the dual ability to infect both plant hosts and resist antagonistic interactions with other soil microorganisms, including amoebal predators.^[^ ^16^ ^]^ These requirements impose strong evolutionary selection pressure on its infection and defense mechanisms, which heavily depend on the synthesis of bioactive natural products.^[^ ^10^ ^]^ Consequently, *P. syringae* has evolved a rich secondary metabolome, with natural products that exhibit diverse and often highly context‐dependent functions. Although members of the species *P. syringae* inhabit similar ecological niches, the species exhibits a remarkable strain‐specific variability in BGCs within the species. For instance, the genome of *P. syringae* DSM 50255 has a BGC count that is 3.5 times higher than that of *P. syringae* DSM 50272 and *P. syringae* DSM 50256 genome has 16 NRPS BGCs, compared to only three in the *P. syringae* DSM 50293 genome. Some of these biosynthetic genes are integral to the core genome, such as those required for the production of pyoverdine siderophores,^[^ ^76^, ^77^ ^]^ highlighting their universal importance across the species. Conversely, many BGCs are located within the accessory genome or exist as singletons with minimal prevalence across the species. These accessory or singleton genes often provide specific ecological advantages and facilitate niche‐specific diversification.^[^ ^78^, ^79^, ^80^ ^]^

A pangenomic analysis of 18 taxonomically distinct *P. syringae* strains enabled the identification of accessory biosynthetic genes with minimal similarity to known *P. syringae* BGCs. We isolated and structurally elucidated the corresponding natural products, syrilipamides A–C, which are cyclic lipopeptides featuring six amino acids with C12 or C14 fatty acid moieties lacking hydroxyl groups at the C‐3′ position. In addition, we identified a novel chlorinated congener of *trans*‐AT type I PKS‐derived secimide A, and subsequently discovered a previously undescribed halogenase that converts secimide A into secimide B via late‐stage halogenation. Docking simulation suggested that SecA, initially classified as a dioxygenase,^[^ ^81^, ^82^ ^]^ could specifically select one of the four secimide A isomers for chlorination, highlighting the enzyme's substrate specificity.

The syrilipamides and secimides show amoebicidal activity individually as well as in combination, and secimides show inhibitory activity selectively against *S. salmonicolor*, suggesting their role in niche adaptation within hostile environments. This detailed characterization of *P. syringae's* unique natural products will further enhance our understanding of how these compounds facilitate the bacterium's capacity to adapt to different niches.

## Conflict of Interests

The authors declare no conflict of interest.

## Supporting information



Supporting Information

Supporting Information

## Data Availability

The data that support the findings of this study are openly available in NCBI at https://www.ncbi.nlm.nih.gov/datasets/genome/GCF_040120475.1/, reference number [REF].
