# Supplementary material for: Pangenome Analysis of the Plant Pathogen Pseudomonas syringae Reveals Unique Natural Products for Niche Adaptation
Source: Angew Chem Int Ed Engl. 2025 May 2;64(25):e202503679. doi: 10.1002/anie.202503679 (PMC12171316; doi:10.1002/anie.202503679)

SUPPORTING INFORMATION

Pangenome Analysis of the Plant Pathogen *Pseudomonas syringae* Reveals Unique Natural Products for Niche Adaptation

Shuaibing Zhang [a], Ying Huang[a], Raed Nachawati[a], Philipp Huber[a], Grit Walther[b], Lucas Gregor[c], Ivan Vilotijević[c,d] and Pierre Stallforth[a,c,d,*]

[a]Leibniz Institute for Natural Product Research and Infection Biology – Hans Knöll Institute, Department of Paleobiotechnology; Beutenbergstraße 11a, D-07745 Jena, Germany

[b]Leibniz Institute for Natural Product Research and Infection Biology – Hans Knöll Institute, National Reference Center for Invasive Fungal Infections; Beutenbergstraße 11a, D-07745 Jena, Germany

[c]Friedrich Schiller University Jena, Institute of Organic Chemistry and Macromolecular Chemistry; Humboldtstraße 10, D-07743 Jena, Germany

[d]Cluster of Excellence Balance of the Microverse, Friedrich Schiller University Jena, Fürstengraben 1 D-07743 Jena, Germany

[*]Email: pierre.stallforth@leibniz-hki.de

Table of Contents

Materials and Methods 4

Bacterial strains and culture conditions 4

Reagents and solvents 4

Genomic DNA extraction, whole genome sequencing and genome assembly 4

Pan-genome analysis of *P. syringae* 4

Whole genome-based phylogenetic analysis of *P. syringae* strains 5

Generation of gene deletion mutants in *P. syringae* 5

Plaque assay 6

Amoebicidal activity testing and IC_{50} determination 6

Antimicrobial Activity of syrilipamides and secimides 6

Heterologous expression of halogenase proteins in *E. coli* 7

Phylogenetic analysis for the α-KG dependent enzymes 7

Phylogenetic analysis for the C starter domain, A domain of β-Ala in *sym* BGC 8

HGT analysis for *P. syringae* DSM 1242 8

Western blot 8

Instruments 8

Metabolic profiling *via* LC-MS 9

Isolation of syrilipamides and secimides 9

Determination of the configuration of the amino acids in lipopeptides using Marfey's reagent 9

Determination of the positions of D- and L-serine in syrilipamides 10

Structure elucidation of syrilipamides 10

Computational analysis of compounds **4** and **5d** 11

Docking simulations of SecA 11

Isolation of secimide A (**5d**) 12

Supplementary Figures 13

Figure S1. Phylogenetic tree of *P. syringae* 13

Figure S2. NRPS and PKS search in the anvi'o platform 14

Figure S3. *sec* and *sym* BGCs were identified using the anvi'o platform 14

Figure S4. Phylogenetic tree based on the whole genomes of *P. syringae* strains 15

Figure S5. HPLC profiles of syrilipamides (**1-3**) and secimides (**4-5**) across diverse *P. syringae* strains in different media 15

Figure S6. Analysis of related gene deletion mutants. 16

Figure S7. Bioinformatic analysis of the *sym* and *sec* BGCs across diverse *P. syringae* strains 17

Figure S8. MS^2-fragmentation of syrilipamide A (**1**) 18

Figure S9. MS^2-fragmentation of syrilipamide B (**2**) 18

Figure S10. MS^2-fragmentation of syrilipamide C (**3**) 19

Figure S11. MS^2-fragmentation of F1. 19

Figure S12. Phylogenetic analysis of $C_{starter}$ and A domain of β-Ala domains of *sym* BGC 21

Figure S13. Eight and four stereoisomers illustrating the relative configuration identification of **4** and **5d**. 21

Figure S14. Comparison between the experimental and theoretical ECD spectra of **4**. 22

Figure S15. NMR data comparison of reported secimide A and compound **5**. 23

Figure S16. Purification of four secimides A isomers (**5a**-**5d**) 24

Figure S17. Analysis for four isomers of secimide A. 25

Figure S18. Phylogenetic analysis of α-KG-dependent enzymes. 26

Figure S19. Phylogenetic and BGC analysis related to SecA.. 27

Figure S20. Amino acid sequence of SecA aligned with three known Fe(II)/α-KG-dependent halogenases 28

Figure S21. Docking simulation of SecA. 30

Figure S22. Amoebal plaque assays for all of the *P. syringae* strains 31

Figure S23. Analysis of ratio of syrilipamides and secimides. 32

Figure S24. Verification of DSM 1242 mutant strains 33

Supplementary Tables 34

Table S1. Results of Marfey's analysis for syrilipamide A (**1**). 34

Table S2. Results of Marfey's analysis for syrilipamide B (**2**). 34

Table S3. Results of Marfey's analysis for syrilipamide C (**3**). 34

Table S4. Results of Marfey's analysis for fragment F1. 34

Table S5.^{1}H NMR (600MHz) data and ^{13}C NMR (150 MHz) of compounds **1−3** (δ in ppm, *J* in Hz) 35

Table S6. The DP4+ probabilities of **4a**-**4h**. 36

Table S7. Experimental CD data and calculated ECD data. 37

Table S8.^{1}H NMR (500MHz) data and ^{13}C NMR (125 MHz) of compounds **4** and **5** (δ in ppm, *J* in Hz) 38

Table S9. The DP4+ probabilities of **5d**. 38

Table S10. Accession numbers for halogenases 39

Table S11. Amoebicidal activity of syrilipamides and secimides. 39

Table S12. Antimicrobial activity assay of syrilipamides and secimides. 39

Table S13. MIC value of secimides against *Sporobolomyces salmonicolor* 549 (μg mL^{-1}). 39

Table S14. Results of the broth dilution method assay for different human pathogenic fungi.. 40

Table S15. List of primers used for amplification and cloning. 41

Table S16. Plasmids used in this study. 42

Table S17. Accession numbers for *sym* and *secD* coding genes in DSM 1242. 42

Table S18. Energy analysis for conformers of **4a**–**4h** at B3LYP/6-31G(d) level 43

References 49

NMR and HRMS spectra 52

Materials and Methods

Bacterial strains and culture conditions

The strains utilized in this investigation were sourced from DSMZ (Leibniz Institute DSMZ) and the Jena Microbial Resource Collection (JMRC). The bacteria were cultured in various media at 28 °C or 22 °C, including Luria Bertani medium (LB, Carl Roth, Germany), brain heart infusion (BHI, Sigma-Aldrich, Germany), SM/5 medium (Formedium™, UK), King's medium B, KB (20 g protease peptone, 1.5 g K_2HPO_4, 1.5 g $MgSO_4 \cdot 7\ H_2O$, and 10 mL glycerol per 1 L ddH_2O), or M9 (containing per 3 g KH_2PO_4, 6.78 g Na_2HPO_4, 1 g NH_4Cl, 0.5 g NaCl, 4 g D-glucose, 4 mL 1 M aqueous $MgSO_4$, 1 mL 10 mM aqueous $FeCl_3$, and 10 mL trace elements solution (2 mg $CaCl_2 \cdot 2H_2O$, 2 mg $ZnSO_4 \cdot 7H_2O$, 2 mg $MnSO_4 \cdot 2H_2O$, 50 mg thiamin, 1 mg biotin) per 1 L ddH_2O).

SM/5 liquid medium was specifically employed for secondary metabolite production. As required, antibiotics were used at the following concentrations: gentamicin 15 or 5 µg mL^{-1}. Bacterial strains were preserved as glycerol stocks and stored at –70 °C.

Reagents and solvents

Reagents used during this study were purchased from ABCR, Sigma-Aldrich, TCI, CDN Isotopes, Alfa Aesar, or Carl Roth. HPLC grade solvents were purchased from VWR. Anhydrous solvents were purchased either from Acros Organics or Alfa Aesar.

Genomic DNA extraction, whole genome sequencing and genome assembly

All previously unsequenced *P. syringae* strains used in this study were cultured for 24 hours for genomic DNA (gDNA) isolation. The gDNA extraction was performed using the QIAamp® DNA Mini kit (Qiagen). Subsequently, the DNA was eluted in nuclease-free water, quantified, and then subjected to Illumina NextGen genome sequencing by Eurofins Genomics GmbH, Constance, Germany. The raw forward and reverse reads underwent quality checks using the fastqc tool (v0.22.9). For genome assembly, the reads were error corrected and assembled using the SPAdes assembler (v3.14).[1] Contigs resulting from the assembly were filtered by size, retaining only those greater than 1,000 bp for further analysis. The contigs were annotated using the Prokka annotation tool (v1.14.0).[2] The Genbank files generated by Prokka were then utilized for the identification of secondary metabolite synthesis-related gene clusters using antiSMASH version 7.0.[3] Subsequently, the genome sequences of these *P. syringae* strains were submitted to NCBI for public access and reference. Genomes of DSM 1241, DSM 50274, DSM 50255, DSM 10604, SZ57, and DC3000 were obtained from the National Center for Biotechnology Information (NCBI).

Pan-genome analysis of *P. syringae*

The pangenome analysis described here primarily adhered to the anvi'o v7.1 pangenomic workflow.[4, 5] The central organization of the pangenome interface, illustrated in the dendrogram, was represented by 'presence/absence' patterns. The core gene bin was characterized by searching the gene homology group, which represents amino-acid sequences from one or more genomes aligned by MUSCLE.[6] Filters were applied with 'Min number of genomes gene homology group occurs, value=18'. The singleton bin was identified by 'Max number of genomes gene homology group occurs, value=1'. All remaining gene clusters that were appended to the accessory bin. NRPS (nonribosomal peptide synthetase)

and PKS (polyketide synthase) genes were characterized by searching for 'functions' using sources such as 'COG20_CATEGORY', 'COG20_PATHWAY', and 'COG20_FUNCTION'. The resulting protein sequences were exported using 'anvi-get-sequences-for-gene-clusters' and subsequently identified in the related genomes. The gene clusters were aligned using Clinker[7] for visualization.

Whole genome-based phylogenetic analysis of *P. syringae* strains

A total of 884 *Pseudomonas syringae* strains were downloaded from NCBI and dereplicated using dRep (version 3.4.2).[8] The filtered dataset, along with our genomes (in total 629 genomes), was subjected to phylogenetic analysis based on the GToTree workflow (version 1.6.31) with the Bacteria.hmm (74 genes) target set for mapping.[9] The resulting sequences were aligned using MUSCLE,[6] trimmed with TRIMAL,[10] and subsequently used to construct a maximum likelihood tree with FastTree2.[11] The genome of *Lysobacter antibioticus* served as an outgroup, and the final phylogenetic tree was visualized using ITоL.[12] A similar phylogenetic analysis was performed for the 18 *P. syringae* strains following the same methodology.

Generation of gene deletion mutants in *P. syringae*

For the generation of marker-less genomic deletion mutants, a gentamicin resistance (*gentR*) selection and sucrose counter-selection (*sacB*) strategy was used. The corresponding pEXG2-based suicide vector[13] was constructed using the Gibson Assembly method. The parent plasmid pEXG2 was linearized using *Hind*III and *EcoR*I restriction enzymes. Left and right homology arms (LA and RA) were PCR-amplified from genomic DNA of DSM 1242 using primer pairs LA fwd/rev and RA fwd/rev, respectively (Table S15). Both primers included an 18 bp sequence adjacent to the PCR fragment complementary to the linearized vector (the primers were designed using: http://nebuilder.neb.com). The LA and RA were ligated into *Hind*III/*EcoR*I-digested pEXG2 vector using the standard Gibson Assembly protocol (New England Biolabs) to yield the respective plasmid pEXG2Δ*goi* (*goi* = gene of interest).

The vectors were transformed into chemically competent *E. coli* DH5α cells *via* heat shock at 42 °C and after 1 h outgrow in SOC medium, cells were plated on LB agar plates containing 15 µg mL^{-1} gentamicin. Single colonies obtained the next day were checked *via* colony PCR for the presence of a plasmid containing the right-sized insert. Positive clones were inoculated in antibiotic-containing liquid medium for plasmid extraction the following day. Plasmids were purified from positive *E. coli* DH5α clones using QIAprep® Spin Miniprep Kit (Qiagen) and then validated by Sanger sequencing. For the knockout procedure in *P. syringae*, 4 mL of the overnight culture in LB was centrifuged and washed twice with 1 mL of 1 mM aq. $MgSO_4$ each time. Finally, the pellet was resuspended in 50 µL of 1 mM aq. $MgSO_4$. Approximately 1 µg of plasmid was added into the suspension, and the mixture was subjected to electroporation at 1500 V. Immediately after electroporation, the transformed bacteria were transferred to 1 mL of BHI medium and incubated with shaking at 28 °C for 3 h. Following incubation, the culture was centrifuged, and the bacterial pellet was plated onto BHI agar supplemented with 5 µg mL^{-1} gentamicin. After 48 hours of incubation at 28 °C, individual colonies were picked and streaked onto fresh BHI agar containing 5 µg mL^{-1} gentamicin. Following an 18-hour incubation at 28 °C, single colonies were selected and grown in 200 µL of LB medium at 28 °C for 4 hours. These cultures were then streaked onto LB plates lacking NaCl but containing 10% (w/w) sucrose to facilitate the selection of double crossover knockout mutants. Colony PCR using DreamTaq Green PCR 2x Master Mix (Thermo Scientific, Darmstadt) and primer pairs

targeting the upstream and downstream regions of the homology arms was employed to confirm the identity of the deletion mutants. As a result, a markerless *goi* deletion mutant was successfully obtained.

Plaque assay

Edibility of *P. syringae* or their mutants were tested using a *Dictyostelium discoideum* AX2-based plaque assay in a 24-well plate format. Briefly, bacterial strains to be tested were first cultured overnight in SM/5 broth. The next day, 30 µL of each of these cultures were added to individual SM/5 agar wells of a 24 well plate (in triplicates) and left to dry for 2 h. A total of 10,000 amoebae cells were added onto individual bacterial lawns. Overnight culture of *K. aerogenes*, a food bacterium for AX2, was used as a positive control for the assay. Plates were subsequently incubated at 22 °C for a period of 5 – 6 days. At the end of the incubation period, wells were checked for the presence of amoebal fruiting bodies (indicating that amoeba could graze successfully on these bacteria) and photographs were taken (Canon EOS 200D camera). Inability of AX2 to form clear plaques or fruiting bodies on any individual bacterium lawn or combinations thereof was taken as an indication of their unpalatability or toxicity. The assay was performed in triplicates.

Amoebicidal activity testing and IC_{50} determination

D. discoideum AX2 was used to assay the amoebicidal activities of syrilipamides and secimides. AX2 cells were cultured in 96-well plates (Sarstedt) with each well containing 2,500 cells in 200 µL HL5 medium. 1 µL of compounds to be tested (dissolved in DMSO with different concentration) were added to respective wells in triplicates (*n* =3) and sterile 1% DMSO served as positive growth control. Plates were then incubated at 22 °C for a period of 72 h. After the incubation period, percentage viability of cells in each well was determined using a resazurin assay. Briefly, the assay is based on the principle that live cells can convert or reduce non-fluorescent blue dye resazurin to the highly fluorescent red dye resorufin. The amount of resorufin formed is directly proportional to the number of viable cells in the well, which correlates directly to the fluorescent intensity values from each well. 20 µL of 1 mM resazurin was added to each well and plates were incubated at 28 °C for a period of 3 h. Fluorescence intensities were then determined using a TECAN plate reader (InFinitePro-M200) set at excitation/emission values of 570/585 nm. The fluorescence intensity values were plotted against the decadic logarithm of the concentration of the compounds or their combinations and the IC_{50} value was determined using PRISM (GraphPad, Version 5.03). The assay was repeated in three biological replicates (*n* = 3) and the final value obtained was the average of all the experiments.

Antimicrobial Activity of syrilipamides and secimides

A disk diffusion assay was conducted for qualitative screening of various microorganisms. Each organism was cultured on Mueller-Hinton agar, and 9 mm wells were created. Compounds were dissolved in a designated solvent to achieve specific concentrations. Then, 50 µL of these solutions were applied to each well. Following a 24-hour incubation at 37°C, the diameters of the inhibition zones were measured.

The activity assay utilized the broth dilution method as outlined in the EUCAST DEFINITIVE DOCUMENT E.DEF 9.3.2.12.[14] *B. cinerea* and *A. solani* were cultured following the NCCLS guidelines for dilution antimicrobial susceptibility tests for bacteria that grow aerobically.[15] Compounds **1-5** were solubilized in DMSO.

Heterologous expression of halogenase proteins in *E. coli* and *in vivo* feeding experiments

The halogenase genes from DSM 1242 genomic DNA were cloned into pET28a plasmids, resulting in plasmids pET28a-cand1, pET28a-cand3, and pET28a-secA, respectively. *E. coli* BL21 (DE3) cells were transformed with the aforementioned plasmids individually, as well as with the blank pET28a vector serving as a negative control. Cultures were then incubated overnight in LB media containing kanamycin (50 µg mL^{-1}) at 37°C and 180 rpm. The pre-culture was inoculated into 20 mL LB media containing kanamycin (50 µg mL^{-1}) and adjusted to an initial OD_{600} of 0.1, followed by incubation at 37°C and 180 rpm. When the culture density reached an OD_{600} of 0.6, cells were maintained at 22°C for 30 minutes and then induced with 1 mM isopropyl-β-D-thiogalactopyranoside (IPTG), shaking at 22°C for 3 h. Next, Bacteria were harvested via centrifugation for 5 min at 4,500 g at 4°C and resuspended in 10 mL M9 media supplemented with 50 µg mL^{-1} kanamycin and 1 mM IPTG. Subsequently, 60 µg of secimide A (**5**) was added to the culture, and the mixture was incubated for 48 hours at 22°C. Afterward, the culture was extracted with 25 mL of ethyl acetate (EtOAc), and the organic phase was dried with Na_2SO_4 and removed *in vacuo*. The residue was dissolved in 300 µL of methanol (MeOH), filtered through a 0.2 µm PTFE syringe filter, and further analyzed *via* LC-HRMS.

Western blot

Following induction with 1 mM IPTG, 10 mL culture of *E. coli* BL21(DE3) harboring the pET28a plasmid was grown at 22°C for 18 h. Cells were harvested by centrifugation at 6000×g for 15 min at 4°C and resuspended in 4 mL of Tris buffer (pH 8.0). Cells were lysed by sonication (40% power, 20 pulses) and centrifuged at 20,000×g for 20 min at 4°C. The supernatant was collected, mixed with loading dye, and heated at 90°C for 10 min (300 rpm). Samples (25 µL) and a 10 µL peqGOLD Protein Marker IV were loaded onto a NuPAGE 4–12% Bis-Tris gel (NP0336BOX) in a mini gel tank containing 1X MOPS buffer. Electrophoresis was performed using a pre-programmed Mini Gel-Blot-Bis-Tris-MOPS method (200v for 35 min). Proteins were transferred to an iBlot 2 NC mini membrane using the P0 program (20 V for 1 min, 23 V for 4 min, 25 V for 2 min).

The membrane was blocked with 5% skim milk in 1X TBS for 2 h at room temperature with shaking, followed by three washes with water. For primary antibody incubation, the membrane was incubated overnight at 4°C in 20 mL of 2.5% milk in 1X TBS containing a 1:2000 dilution of anti-6XHis monoclonal antibody (Thermo Fischer) with shaking. After three washes with 1X TBS, the membrane was incubated for 2 h at room temperature in 20 mL of 2.5% milk in TBS containing a 1:10,000 dilution of secondary Goat anti-mouse IgG (H+L) antibody (Thermo Fischer). Following three 5-min washes with 1X TBS, protein bands were detected using Novex AP chromogenic substrate (WP20001) with shaking for 10 min. Membranes were imaged for documentation.

Phylogenetic analysis for the α-KG dependent enzymes

Phylogenetic analysis was conducted based on the GToTree workflow (version 1.6.31).[9] The phylogenetic tree includes the secA along with other reported α-KG dependent enzymes from the UniProt database to ensure a comprehensive and representative comparison. All sequences were dereplicated prior to analysis to avoid duplicates by CD-Hit (version 4.8.1).[16, 17] These sequences were aligned using MUSCLE[18] and trimmed using TRIMAL.[10] The

trimmed alignment was then used to construct a maximum likelihood tree using FastTree2.[11] The tree was visualized using iTOL tree viewer.[19]

Phylogenetic analysis for the C starter domain, A domain of β-Ala in *sym* BGC, and secA related enzymes

Phylogenetic analysis was conducted based on the GToTree workflow (version 1.6.31).[9] The sequences for the phylogeny were obtained from BLASTP (Blast version 2.12.0+) search with a maximum target sequence number of 1000, and dereplicated by CD-Hit (version 4.8.1).[16, 17] These sequences were aligned using MUSCLE[18] and trimmed using TRIMAL.[10] The trimmed alignment was then used to construct a maximum likelihood tree using FastTree2.[11] The tree was visualized using iTOL tree viewer.[19] All of the aligned BGCs showed in this study were generated by Clinker.[7]

HGT analysis for *P. syringae* DSM 1242

The evolutionary history of *P. syringae* DSM 1242 was investigated through horizontal gene transfer (HGT) analysis using HGTector2.[20] Protein sequence similarity searches were conducted with DIAMOND[21] under default parameters. The searches utilized a comprehensive database generated by HGTector on 2023-01-02, based on NCBI RefSeq release 215. This database comprises 129,809,746 unique protein sequences derived from 40,310 microbial genomes, representing three domains of life, 75 phyla, 153 classes, 356 orders, 847 families, 4,095 genera, and 40,309 species. The extensive dataset enabled robust inference of HGT events, providing insights into the evolutionary processes shaping the genome of DSM 1242. This analysis identified 569 genes with plausible HGT origins (please check in Table S28). However, the syrilipamide biosynthetic gene cluster (including the Cstarter domain and A domain of β-alanine incorporation) was not among these identified genes.

Instruments

LC-MS Analysis: For the detection of secondary metabolites from mono- and co-cultures, LC-MS measurements were performed on a Shimadzu UHPLC-MS System (LC-30AD, SPD-M30A, and LCMS-2020). The system is equipped with an electrospray ion source and a Kinetex® C18 column (50 x 2.1 mm, particle size 1.7 µm, pore diameter 100 Å, Phenomenex). Column oven was set to 40 °C; scan range of MS was set to *m/z* 150 to 2,000 with a scan speed of 10,000 u/s and event time of 0.25 s under positive and negative mode. Desolvation line temperature was set to 250 °C with an interface temperature of 350 °C and a heat block temperature of 400 °C. The nebulizing gas flow was set to 1.5 L min^{-1} and dry gas flow to 15 L min^{-1}. If not otherwise stated a standard LC-method was used: flow rate = 0.7 mL min^{-1}; 0 – 0.5 min: 10% (v/v) acetonitrile (MeCN) in water containing 0.1% formic acid; 0.5 – 8.5 min: linear gradient 10 – 100% MeCN in water containing 0.1% formic acid; 8.5 – 11.5 min: 100% MeCN in water containing 0.1% formic acid, 11.5 – 13 min: 10% MeCN in water containing 0.1% formic acid, 13 – 14 min: 10% MeCN in water containing 0.1% formic acid, injection volume: 10 µL. For the Luna® Omega PS C18 column (100 x 2.1 mm, particle size 1.6 µm, pore diameter 100 Å, Phenomenex), parameters setting is the same as the Kinetex® C18 column, the only difference is LC method: flow rate = 0.2 mL min^{-1}; 0 – 0.5 min: 10% (v/v) MeCN in water containing 0.1% formic acid; 0.5 – 20.5 min: linear gradient 10 – 100% MeCN in water containing 0.1% formic acid; 20.5 – 28.5 min: 100% MeCN in water containing 0.1% formic acid, 28.5 – 32 min: 10% MeCN in water containing 0.1% formic acid, 32 – 40 min: 10%

MeCN in water containing 0.1% formic acid. LC-MS results were analyzed using LabSolutions Postrun and Browser (v.5.60).

Nuclear Magnetic Resonance (NMR) Spectroscopy: NMR spectra of peptides were recorded on Bruker Avance III 600 spectrometers. Deuterated NMR solvents were purchased from VWR. Chemical shift δ are reported in ppm, coupling constants J in Hz. The residue proton signals of the respective solvent were used as internal standards (DMSO-d_6: δ = 2.50 ppm (^{1}H), δ = 39.51 ppm (^{13}C). The signal fine structures are described, using the following abbreviations: s (singlet), d (doublet), t (triplet), q (quartet) and m (multiplet) as well as combinations of these. Spectra were analyzed using Bruker TopSpin software.

High Resolution Mass Spectrometry (HRMS): LC-ESI-HRMS measurements were performed on a Q-Exactive Plus Hybrid Quadrupole Orbitrap masss pectrometer using electrospray ionization and a Dionex UltiMate 3000 UHPLC system (Thermo Fisher Scientific) equipped with a Kinetex C18 column (2.1 × 150 mm, 2.5 µm, 100 Å, Phenomenex). The analytical method is a gradient elution of solvents A (water, 0.1% formic acid) and B (acetonitrile, 0.1% formic acid) at a flow rate of 0.3 mL/min: 5% B for 0.5 min, a linear gradient to 97% B for 11.5 min, then 97% B for 3 min, and 5% B for 3 min. For MS^2 measurements, higher-energy collisional dissociation (HCD) was used at a normalized collision energy of 30. HRMS data was analyzed using the XCalibur software (Thermo Fisher Scientific).

Metabolic profiling *via* LC-MS

The samples were prepared as follows: 10 mL of SM/5 culture from the respective bacterial strains were extracted with 25 mL of EtOAc. The organic phase was collected, and the solvent was removed under vacuum. The resulting residue was dissolved in 300 µL of MeOH, filtered through a 0.2 µm PTFE syringe filter, and subsequently analyzed *via* LC-MS.

Isolation of syrilipamides and secimides

After the incubation period, 8 L of SM/5 culture were extracted with EtOAc. The organic phase was subsequently dried over Na_2SO_4, and the solvents were removed *in vacuo*. The resulting residue, weighing 1.2 g, was then fractionated using a C18 cartridge containing 30 g of stationary phase. Elution was performed using a gradient of 10%, 25%, 50%, 75%, and 100% (v/v) MeCN in water. LC-MS analysis revealed that the 25% fraction contained secimides, while the 50% fraction contained syrilipamides. These fractions were further purified using semi-preparative HPLC (Shimadzu) equipped with a Luna® C18(2) column (250 × 10 mm, 5 µm, 100 Å, Phenomenex), with a flow rate of 5 mL min^{-1}. For purification of syrilipamides: Method: 0 – 1.5 min: 10% (v/v) MeCN in water, 1.5 – 5 min: gradient from 10% to 50% MeCN in water, 5 – 20 min: gradient from 50% to 65% (v/v) MeCN in water, yielding the following NRPS products: syrilipamide A (**1**, 3.2 mg) at t_R = 15.0 min, syrilipamide B (**2**, 3.0 mg) at t_R = 18.5 min, syrilipamide C (**3**, 2.6 mg) at t_R = 21.0 min. The amino acid sequences of the pyrofactins were determined by LC-MS/MS analysis. For purification of secimides: Method: 0 – 10 min: 26% (v/v) MeCN in water, yielding secimide A (**5**) (mixture, four diastereomers, 40.0 mg) at t_R = 8.6 min; further purification yielded four diastereomers at t_R = 16.7 min (**5a**, 8.0 mg), t_R = 17.7 min (**5b**, 8.0 mg), t_R = 19.0 min (**5c**, 6.5 mg) and t_R = 19.7 min (**5d**, 6.0 mg) with the method 0 – 20 min: 20% (v/v) MeCN in water; Method: 0 – 20 min: 20% (v/v) MeCN in water, yielding secimide B (**4**, 10.0 mg) at t_R = 14.4 min.

Determination of the configuration of the amino acids in lipopeptides using Marfey's reagent

Briefly, 250 µL 6 N aq. HCl were added to 100 – 250 µg of the respective peptide. The reaction vessel was heated to 100 °C and shaken at that temperature for 16 h. Solvent was removed *in vacuo* and the residue was dissolved in 100 µL water. 200 µL Marfey's reagent (1-fluoro-2,4-dinitrophenyl-5-L-alanine amide, FDAA, 1% solution in acetone, Thermo Scientific) and 40 µL 1 M aq. $NaHCO_3$ solution were added. The reaction was shaken for 1 h at 40 °C. Then, the reaction was quenched with 20 µL 2 N aq. HCl and filtered through a PTFE filter (0.2 µm). Analysis was performed on a semipreparative HPLC (Shimadzu) equipped with a Luna® C18(2) column (250 × 4.6 mm, 5 µm, 100 Å, Phenomenex, flow rate = 1 mL min^{-1}, method: 0 – 1 min: 25% (v/v) MeCN in water containing 0.1% formic acid; 1 – 46 min: linear gradient 25 – 65% MeCN in water containing 0.1% formic acid). Amino acids (5 µmol) for comparison were dissolved in 100 µL water and treated in the same manner.[22]

Standard retention times (in min):
L-Ser (t_R = 9.16); D-Ser (t_R = 9.48); L-Homo-Ser (Hse) (t_R = 9.83); D-Hse (t_R = 10.22); β-Ala (t_R = 13.51); L-Val (t_R = 20.12); D-Val (t_R = 24.41); L-Leu (t_R = 25.05); D-Leu (t_R = 29.23).

Determination of the positions of D- and L-serine in syrilipamides by $Sc(OTf)_3$-mediated hydrolysis followed by Marfey's analysis

The hydrolysis was carried out according to literature protocols.[23] A quantity of 2 mg (2.7 µmol, 1 eq.) of syrilipamide A was first linearized using 0.01 M LiOH and then directly processed for hydrolysis with $Sc(OTf)_3$. The linearized syrilipamide A, along with 33.4 mg (67.5 µmol, 25 eq.) of $Sc(OTf)_3$, was dissolved in a 1000 µL solvent mixture consisting of MeCN and water in a 2:1 volume ratio. While stirring continuously, the reaction mixture was heated to 90 °C and maintained at this temperature for a duration of 12 hours. After allowing the reaction to cool down to room temperature, it was extracted with EtOAc (3 extractions, each with 1.0 mL). The organic phases were combined, and the solvent was subsequently removed under vacuum. The resulting crude extract underwent purification using semi-preparative HPLC (Shimadzu) equipped with a Luna® C18(2) column (dimensions: 250 x 10 mm, 5 µm, 100 Å, Phenomenex). The purification method employed a flow rate of 5 mL min^{-1} and a gradient elution program: 0-2 minutes with 50% (v/v) MeCN in water containing 0.1% formic acid, followed by a linear gradient from 50% to 55% MeCN in water containing 0.1% formic acid over the course of 2 to 17 minutes. LC-HRMS analysis of the collected fractions indicated that fragment F1 with a measured mass of 500.3328 (calculated mass: 500.3331) for the ion $[C_{25}H_{46}N_3O_7]^+$. The amino acid sequence of fragment F1 was further confirmed through LC-MS/MS analysis.

Structure elucidation of syrilipamides

Syrilipamides A (**1**), B (**2**), and C (**3**) were isolated as white powder. According to Marfey's analysis, these compounds are composed of one L-leucine, one D-serine, one L-homo-serine, one β-alanine, one L-valine, and one L-serine residue each. Positive HRESIMS analysis further determined their molecular formulae to be $C_{36}H_{62}O_{10}N_6$, $C_{36}H_{64}O_{10}N_6$, and $C_{38}H_{66}O_{10}N_6$, respectively, revealing nine, eight, and nine degrees of unsaturation. This suggests the existence of one or two additional rings in addition to what Marfey's analysis indicates. This is supported by HMBC correlations observed between hydrogens H-11 (**1**: δ_H 4.42; **2**: δ_H 4.43; **3**: δ_H 4.43) and C-30 (**1**: δ_C 170.04; **2**: δ_C 170.01; **3**: δ_C 170.01), implying a cyclic peptide structure. Moreover, the presence of a *cis*-double bond in both **1** and **3** was confirmed by the coupling constant between H-5' and H-6' (9.9 Hz) in **1**, and between H-7' and H-8' (10.5 Hz) in **3**. The identification and order of the other amino acids were determined using COSY and HMBC

spectra. The positions of D-serine and L-serine were identified by $Sc(OTf)_3$ partial hydrolysis and subsequent Marfey's analysis.

Computational analysis of compound 4 and 5d

Conformational analyses were conducted for isomers **4a–4h** utilizing molecular mechanics with the MMFF94s force field in Spartan'20 (Wavefunction, Inc. Irvine, CA). Conformers with a population higher than 1% were selected for further optimization. Optimization was performed using density functional theory (DFT) at the B3LYP/6-31G(d) level in the Gaussian 16 program package. This process yielded six (**4a1** to **4a6**), five (**4b1** to **4b5**), eight (**4c1** to **4c8**), ten (**4d1** to **4d10**), twelve (**4e1** to **4e12**), five (**4f1** to **4f5**), five (**4g1** to **4g5**), and five (**4h1** to **4h5**) predominant conformers within a 3.0 kcal/mol energy threshold from a global minimum. For these predominant conformers NMR spectra predictions were performed. Gauge-Independent Atomic Orbital (GIAO) predictions of ^{13}C NMR spectra were performed using density functional theory (DFT) at the MPW1PW91/6-311+g(d,p) level in DMSO with the PCM model. The calculated NMR data of these conformers were averaged according to the Boltzmann distribution theory and their relative Gibbs free energy.

The relative and absolute configurations of compound **4** were assigned *via* computational methods. Utilizing the DP4+ probability,[24-26] the relative configuration of **4** was determined. Among the eight observed stereoisomers, (3*R*,4*R*,8*R*,11*S*)-**4** (**4d**) was established with 98.24% confidence. NOESY data further supported the relative configurations of 3*R**,4*R**, evidenced by the correlation between H-4 (δ_H 2.71) and H-3 (δ_H 4.73). For the determination of absolute configuration, ECD calculations employing time-dependent density functional theory (TD-DFT) method at the CAM-B3LYP/6-311+G(d,p) level[27] in methanol with the PCM model were conducted. Calculated ECD spectra for (3*R*,4*R*,8*R*,11*S*)-**4** (**4d**), (3*S*,4*S*,8*S*,11*R*)-**4** (**4dm**), and their weighted ECD were generated using SpecDis 1.71 with σ = 0.3 eV.[28] Comparison of theoretically calculated and experimental ECD spectra showed that the calculated curves of **4d** were similar to the experimental data, indicating the absolute configuration of **4** as 3*R*,4*R*,8*R*,11*S*.

Secimide A (**5**), isolated as a yellow oil with a molecular formula of $C_{16}H_{23}O_6N$, presented as a mixture of four isomers (**5a**, **5b**, **5c**, and **5d**). Upon comparison of the NMR data between compounds **4** and **5**, it was deduced that **4** is a 3-chloro derivative of **5a**. This assignment was corroborated by the absence of C-3 (δ_C 58.06) in **4**, while C-3 (δ_C 38.04) appeared in **5**. The sole difference between **5a** and **5b** is the stereogenic center at C-11, whereas the difference between **5c** and **5d** is the stereorgenic center at C-13. In **5a** and **5b**, a double bond is situated at the C-12/C-13 position, whereas in **5c** and **5d**, it is at the C-11/C-12 position. Notably, due to the facile isomerization of the double bond from the C-11/C-12 position to the C-12/C-13 position, the interconversion among the four isomers occurs rapidly.

However, under cold conditions (5°C for growth, followed by freeze-drying for extraction), we successfully purified isomer 5d. Its absolute configuration was assigned using computational methods, as described above. The four candidate structures (**5a–5d**) were considered, with absolute configurations of 8*R*,13*R*; 8*R*,13*S*; 8*R*,11*R*; and 8*R*,11*S*, respectively. DP4+ probability analysis determined the relative configuration of **5d** to be 8*R*,11*S*. Given that the absolute configuration at C-8 should be consistent with compound **4**, the absolute configuration of **5d** was therefore assigned as 8*R*,11*S*.

Docking simulations of SecA

Molecular docking studies were performed on the SecA protein using AutoDock4 and AutoDock Vina.[29, 30] Initially, the structure of SecA was predicted using AlphaFold2.[31] Ligand geometries were optimized using Spartan'20 (Wavefunction, Inc., Irvine, CA) and Gaussian 16[32], employing the B3LYP-D3(BJ)/6-31G(d) level of theory.[33] Covalent docking parameters were prepared with MGLTools in conjunction with AutoDock4 and AutoDockTools. The docking box was centered on a grid of (-3.683, -0.343, -6.145) Cartesian x, y, z points, spaced at 0.375 Å. AutoDock Vina simulations were exhaustively run (32 runs) in a 126 Å box to cover the whole protein.

Isolation of secimide A (5d)

Overnight cultures grown in SM/5 medium were harvested via centrifugation at 4,500 × *g* for 5 minutes at 4°C, followed by washing the cell pellets twice with cold PBS buffer. The washed pellets were inoculated into fresh SM/5 medium at an initial OD_{600} of 2.0 and incubated at 5°C with shaking at 180 rpm for 96 hours. After the incubation period, 1 L of the culture was freeze-dried to obtain a residue, which was subsequently dissolved in methanol and filtered for LC-MS analysis and purification. LC-MS analysis confirmed the production of a single isomer of secimide A (**5d**) and secimide B (**4**) under these conditions. The sample was further purified using semi-preparative HPLC (Shimadzu) equipped with a Luna® C18(2) column (250 × 10 mm, 5 µm, 100 Å, Phenomenex) at a flow rate of 5 mL min^{-1}. The purification method involved an isocratic of 20% (v/v) acetonitrile in water over 21 minutes, yielding secimide A (**5d**, 1.0 mg) at a retention time (t_R) of 19.75 minutes.

Supplementary Figures

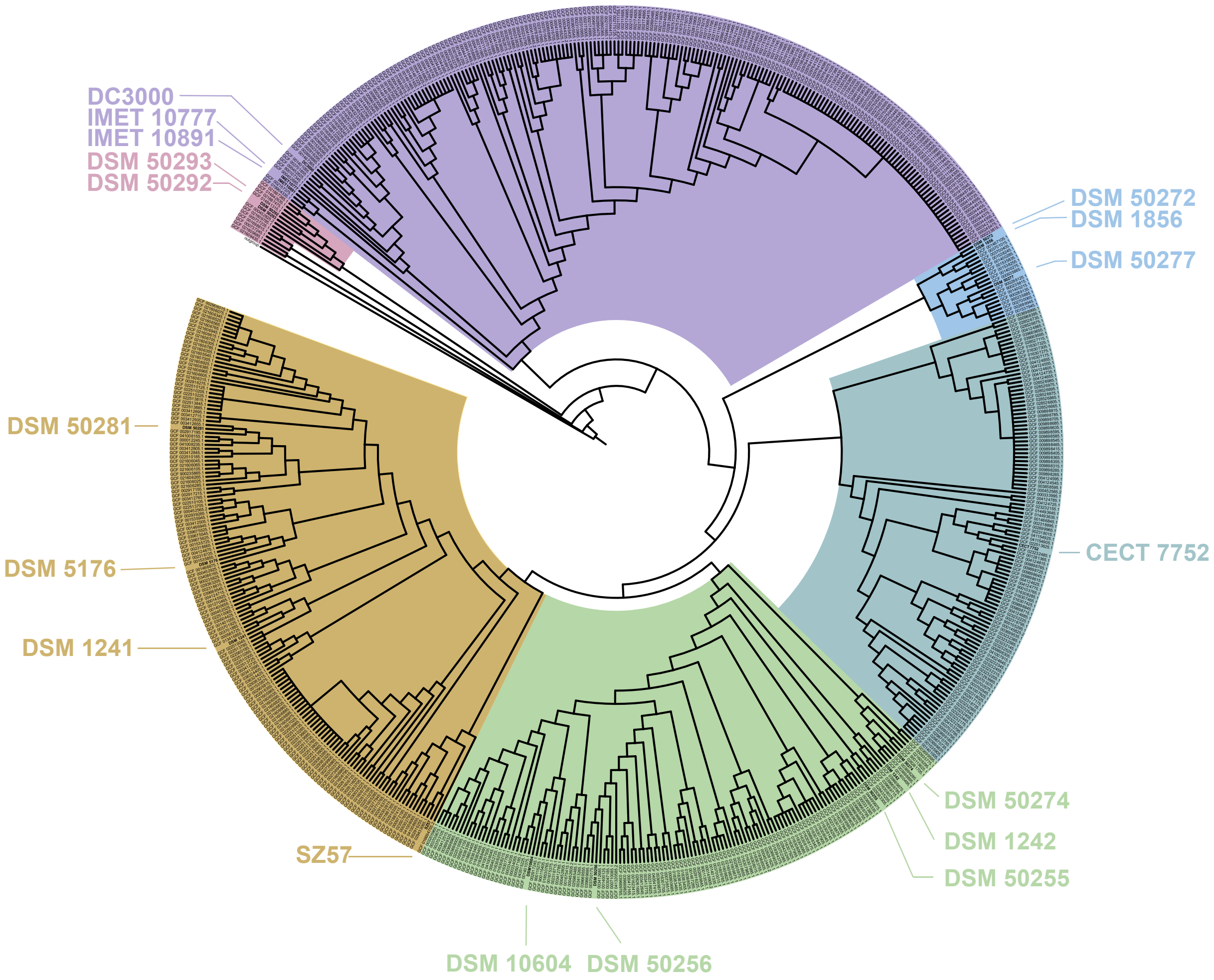

Figure S1. Phylogenetic tree of *P. syringae.* The phylogenetic tree was constructed based on the whole-genome sequences of 884 *P. syringae* strains deposited in the NCBI database. From this dataset, 18 *P. syringae* strains representing the six major subgroups identified in the phylogenetic tree were selected for this study.

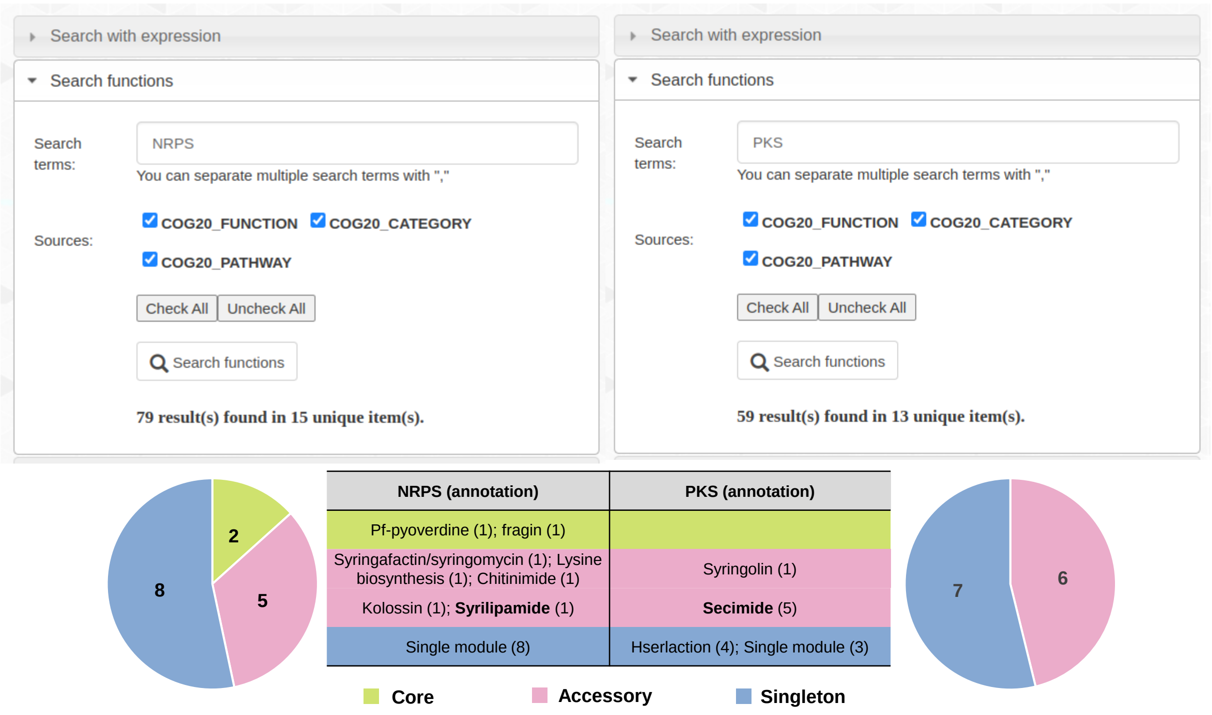

Figure S2. NRPS and PKS search in the anvi'o platform with the further annotation of candidate genes. Numbers in parentheses indicate how many hit genes the product has.

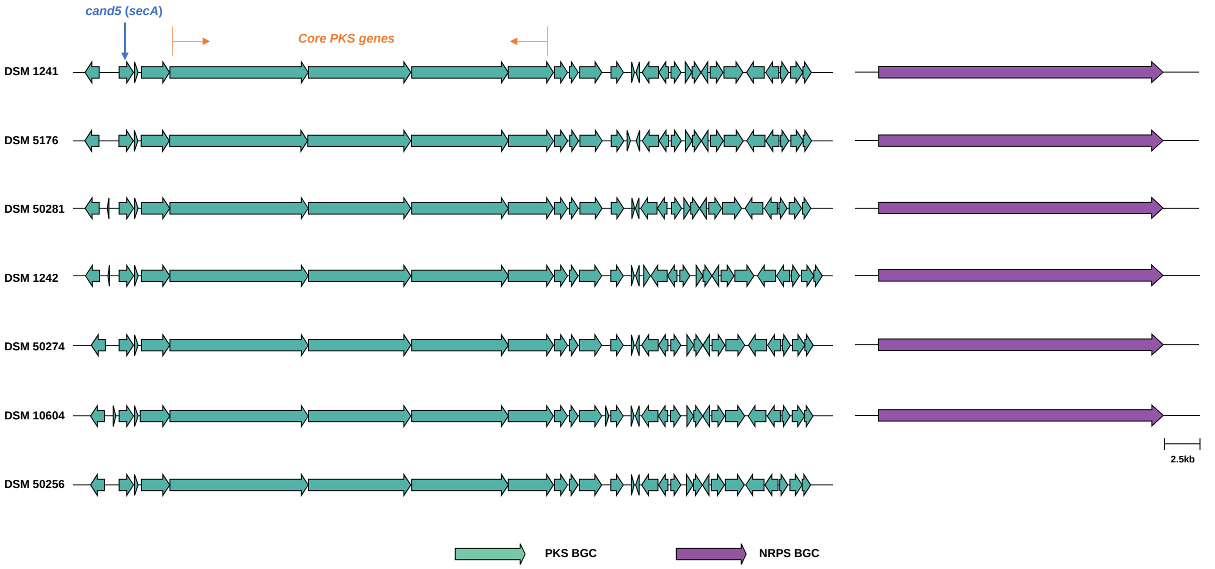

	PKS BGC														
			secA	*secB*	*secC*	*secD*	*secE*	*secF*	*secG*	*secH*	*secI*	*secJ*	*secK*	*secL*	*secM*
**DSM1241**	WP_024640791.1		WP_024640792.1	WP_003399878.1	WP_231992689.1	WP_080346768.1	WP_032653302.1	WP_032653299.1	WP_032653296.1	WP_032653293.1	WP_032607420.1	WP_024641320.1	WP_024641319.1	WP_003313844.1	WP_024639707.1
**DSM5176**	WP_024640791.1		WP_058822775.1	WP_058822776.1	WP_235595641.1	WP_349344634.1	WP_060409426.1	WP_349344633.1	WP_060409425.1	WP_024639703.1	WP_349344632.1	WP_024639705.1	WP_024639706.1	WP_003399908.1	WP_024639707.1
**DSM50281**	WP_349345592.1	WP_231992691.1	WP_116831039.1	WP_003399878.1	WP_231992689.1	WP_116831040.1	WP_349345591.1	WP_349345590.1	WP_349345589.1	WP_024639703.1	WP_060409424.1	WP_116831044.1	WP_162885260.1	WP_003313844.1	WP_024639707.1
**DSM1242**	WP_346394924.1	WP_162889022.1	WP_162889022.1	WP_010418193.1	WP_003399878.1	WP_346394925.1	WP_346394926.1	WP_346394927.1	WP_346394929.1	WP_198700444.1	WP_003392075.1	WP_346394930.1	WP_074909835.1	WP_232913843.1	WP_116817992.1
**DSM50274**	WP_024650538.1		WP_057406049.1	WP_003399878.1	WP_235592770.1	WP_080391926.1	WP_074951496.1	WP_080391925.1	WP_057406047.1	WP_057406046.1	WP_044312197.1	WP_044312196.1	WP_232913843.1	WP_003340813.1	WP_044312193.1
**DSM10604**	WP_024959333.1	WP_154232058.1	WP_024664313.1	WP_003399878.1	WP_235810960.1	WP_080278431.1	WP_024959329.1	WP_024959327.1	WP_024959326.1	WP_032628809.1	WP_024959324.1	WP_024959323.1	WP_024959322.1	WP_024959321.1	WP_024959320.1
**DSM50256**	WP_024650538.1		WP_029571526.1	WP_003399878.1	WP_246765959.1	WP_349347405.1	WP_349347404.1	WP_080288473.1	WP_029571523.1	WP_003392075.1	WP_029571522.1	WP_029571521.1	WP_010418266.1	WP_003313844.1	WP_024663132.1

	PKS BGC														NRPS BGC
	secN'	*secN*	*secO*	*secP*	*secQ*	*secR*	*secS*	*secT*	*secU*	*secV*	*secW*	*secX*	*secY*	*secZ*	
**DSM1241**	WP_024641318.1	WP_032653289.1	WP_011268948.1	WP_010426725.1	WP_003399918.1	WP_024640221.1	WP_024640222.1	WP_003399924.1	WP_177410931.1	WP_003399927.1	WP_003399930.1	WP_024642208.1	WP_024642209.1	WP_003399936.1	WP_253915621.1
**DSM5176**	WP_349344631.1	WP_011268949.1	WP_011268948.1	WP_054092212.1	WP_003399918.1	WP_057457205.1	WP_060405823.1	WP_003399924.1	WP_240879690.1	WP_024650680.1	WP_003399930.1	WP_024640224.1	WP_024642209.1	WP_003399936.1	WP_349344608.1
**DSM50281**	WP_096127261.1	WP_116831046.1	WP_003399914.1	WP_010426725.1	WP_058399509.1	WP_003399920.1	WP_024650682.1	WP_003399924.1	WP_205417546.1	WP_003399927.1	WP_003399930.1	WP_024640224.1	WP_003399934.1	WP_003399936.1	WP_349345885.1
**DSM1242**	WP_004416042.1	WP_349341283.1	WP_003368738.1	WP_016569021.1	WP_003313837.1	WP_003392086.1	WP_004411882.1	WP_003313834.1	WP_003340788.1	WP_183145851.1	WP_349341282.1	WP_003313831.1	WP_346394933.1	WP_024693196.1	WP_349341507.1
**DSM50274**	WP_057406044.1	WP_003368738.1	WP_003424658.1	WP_003313837.1	WP_003392086.1	WP_003313835.1	WP_057406043.1	WP_003340788.1	WP_183131170.1	WP_057406041.1	WP_003313831.1	WP_003313830.1	WP_057406040.1	WP_003313828.1	WP_058877201.1
**DSM10604**	WP_024959319.1	WP_003313837.1	WP_003392086.1	WP_024959318.1	WP_024959317.1	WP_003340788.1	WP_024959316.1	WP_003313832.1	WP_003313831.1	WP_003313830.1	WP_024959315.1	WP_003313828.1	WP_003313827.1	WP_024640227.1	WP_024960041.1
**DSM50256**	WP_122243128.1	WP_096137838.1	WP_004416046.1	WP_002555259.1	WP_003392086.1	WP_074909841.1	WP_346397730.1	WP_003340788.1	WP_183134329.1	WP_003313832.1	WP_116895461.1	WP_003424653.1	WP_003392094.1	WP_003313828.1	

Figure S3. Top: *sec* and *sym* BGCs were identified using the anvi'o platform. Bottom: accession number of representative genes.

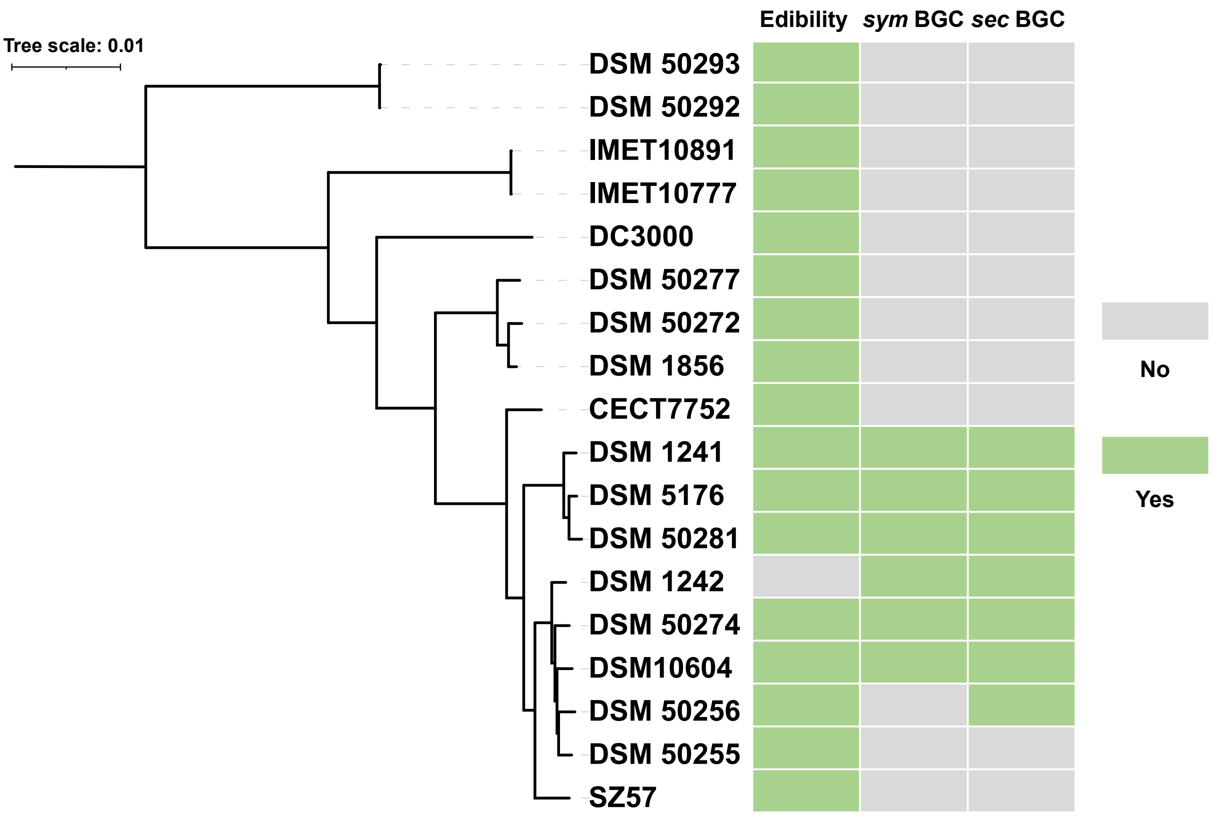

Figure S4. Phylogenetic tree based on the whole genomes of all sequenced *P. syringae* strains, showing the presence of *sym* and *sec* BGCs within certain strains, as depicted using the anvi'o platform, alongside an assessment of their edibility to *D. discoideum* AX2.

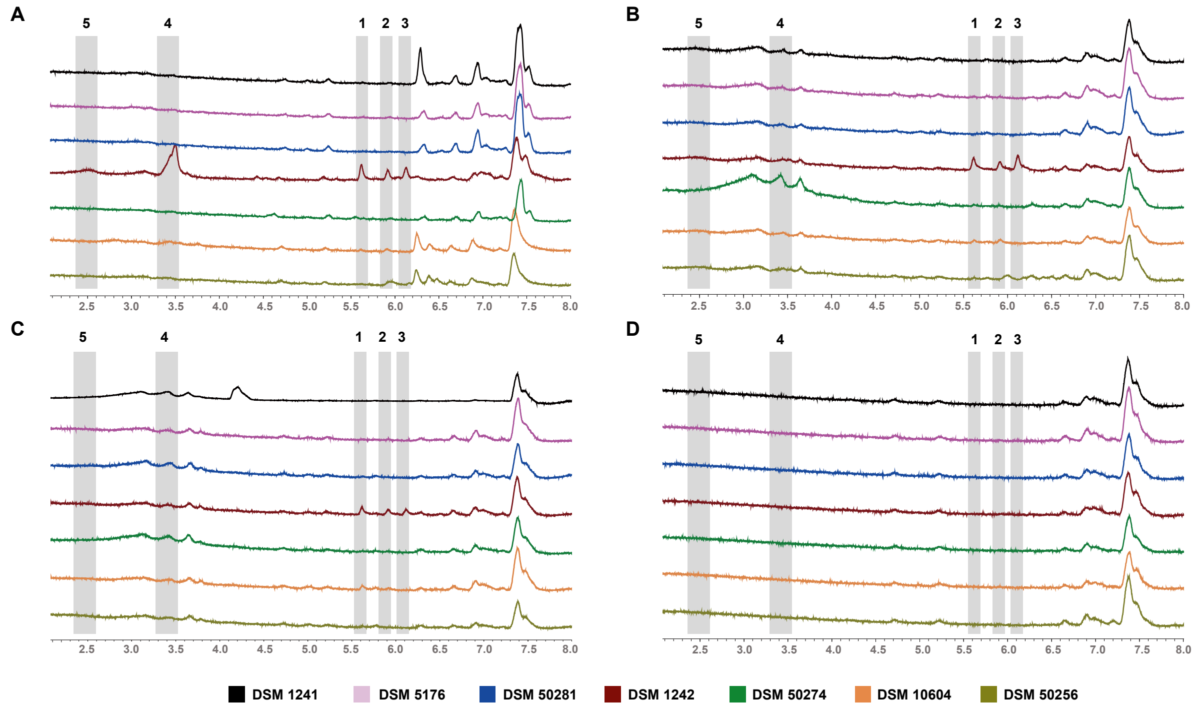

Figure S5. HPLC profiles of syrilipamides (**1-3**) and secimides (**4-5**) across diverse *P. syringae* strains in different media (UV detected at λ=190 nm). (A) SM/5 medium. (B) KB medium. (C) LB medium. (D) M9 medium.

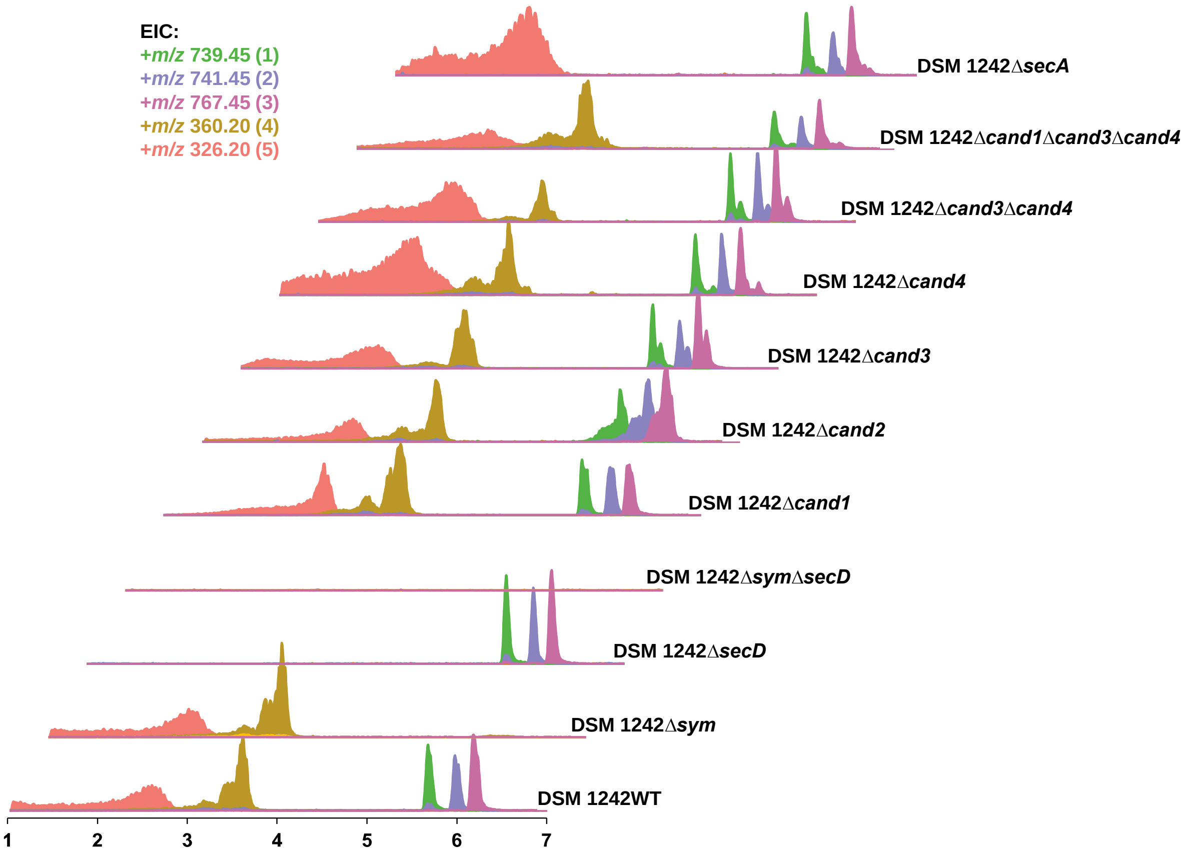

Figure S6. Analysis of related gene deletion mutants. Extracted ion chromatograms (EIC) showing i) syrilipamides (**1**-**3**) were not detected in the DSM 1242Δ*sym* mutant; ii) secimides (**4**-**5**) were gone in the DSM 1242Δ*secD* mutant; iii) both types of molecules disappeared in the DSM 1242Δ*sym*Δ*secD* mutant; iv) secimide B (**4**) was not present in the DSM 1242Δ*secA* mutant, with a concomitant increase in the production of secimide A (**5**).

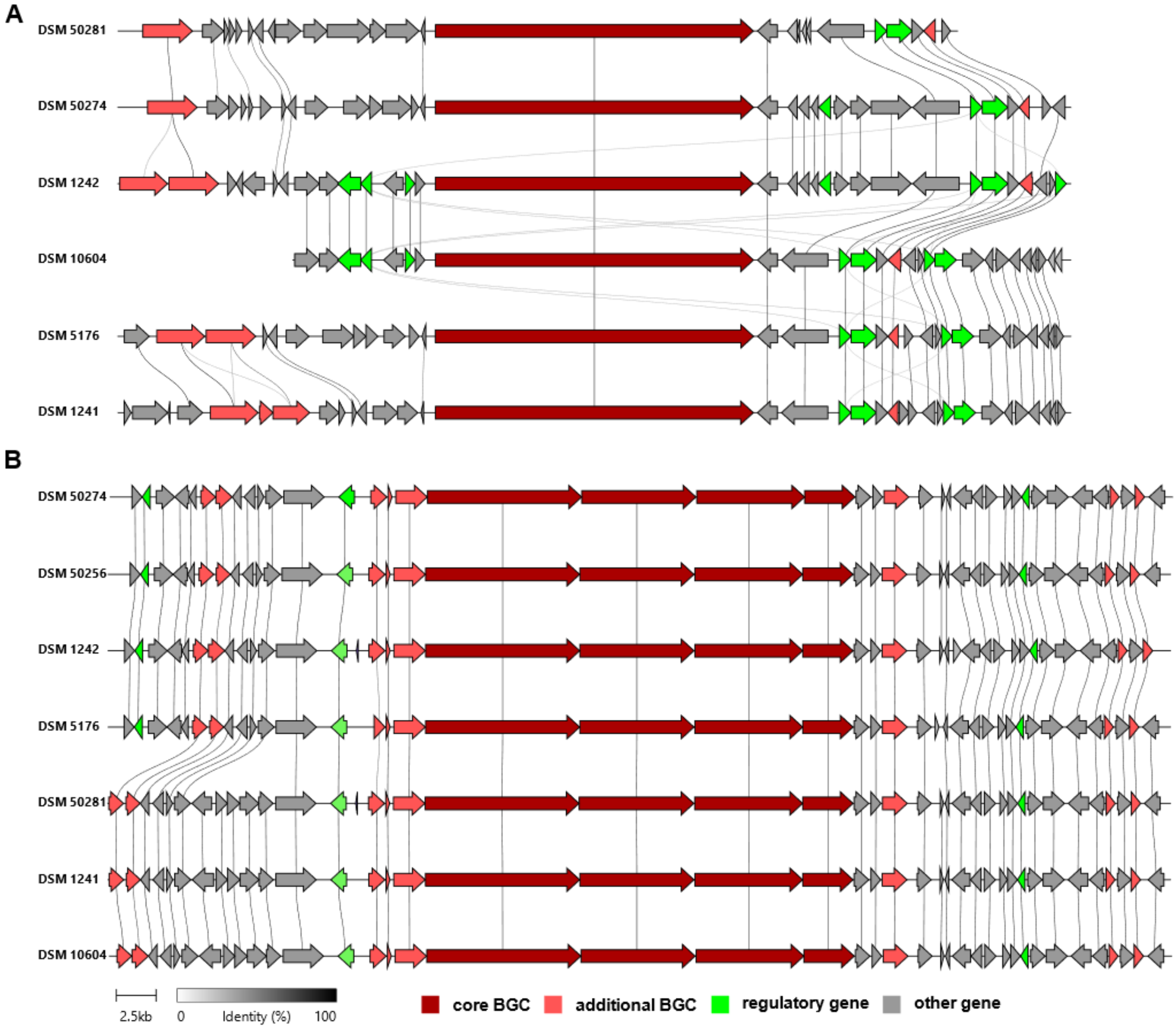

Figure S7. Bioinformatic analysis of the *sym* and *sec* BGCs across diverse *P. syringae* strains. (A) Bioinformatic analysis of the *sym* BGC in six *P. syringae* strains reveals that DSM 1242 contains a greater number of regulatory genes located near the core NRPS BGC. (B) Bioinformatic analysis of the *sec* BGC in seven *P. syringae* strains suggests that the regulatory gene marked on the left is crucial for secimide production, while the first gene on the right is hypothesized to encode a poly-(aspartic acid) hydrolase, which may be involved in the degradation of secimides.

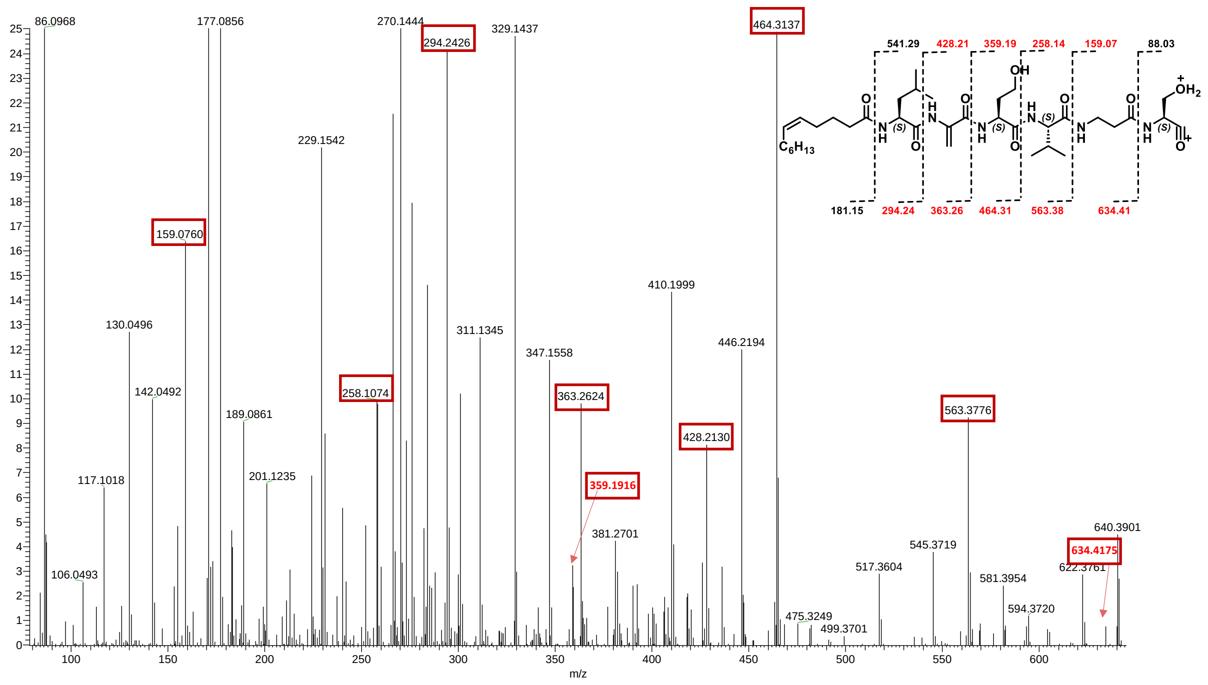

Figure S8. MS^2-fragmentation of syrilipamide A (**1**)

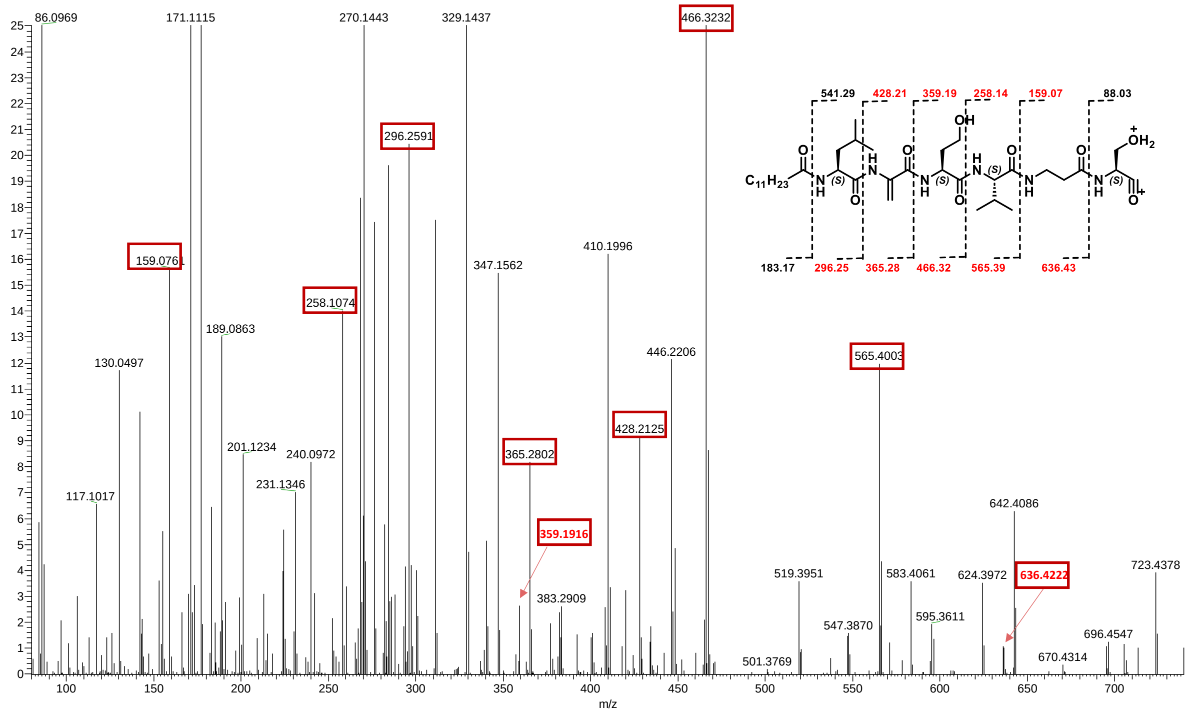

Figure S9. MS^2-fragmentation of syrilipamide B (**2**)

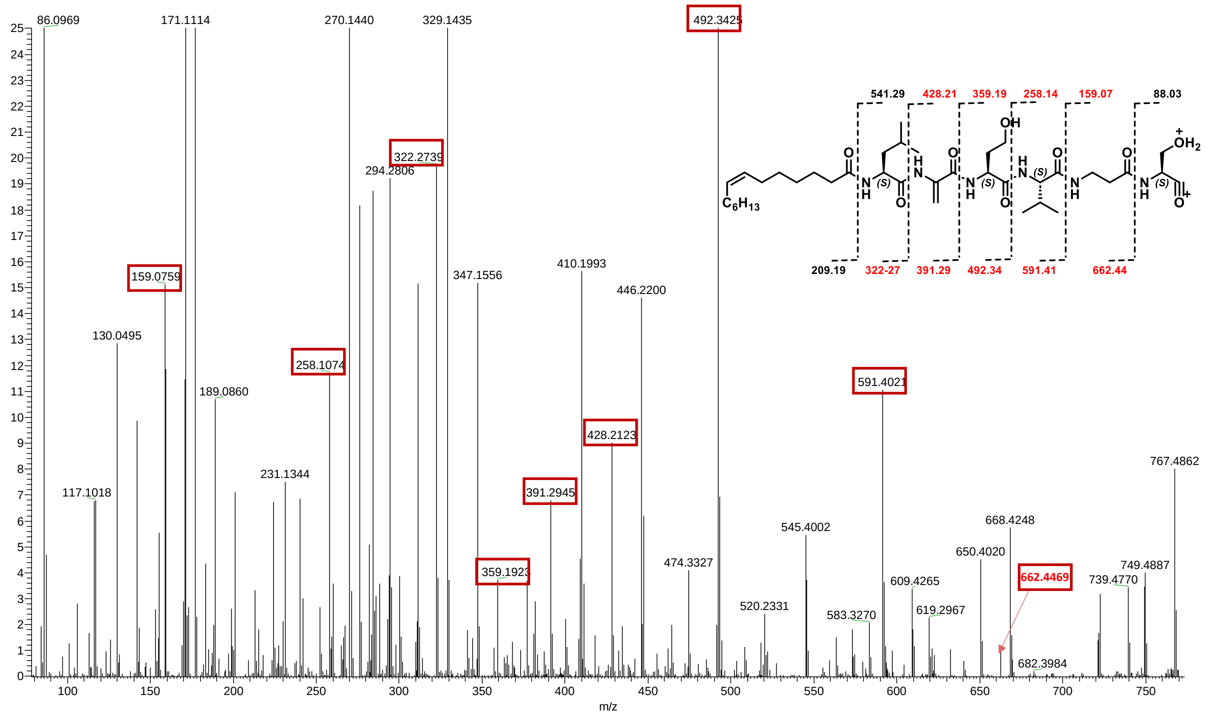

Figure S10. MS^2-fragmentation of syrilipamide C (**3**)

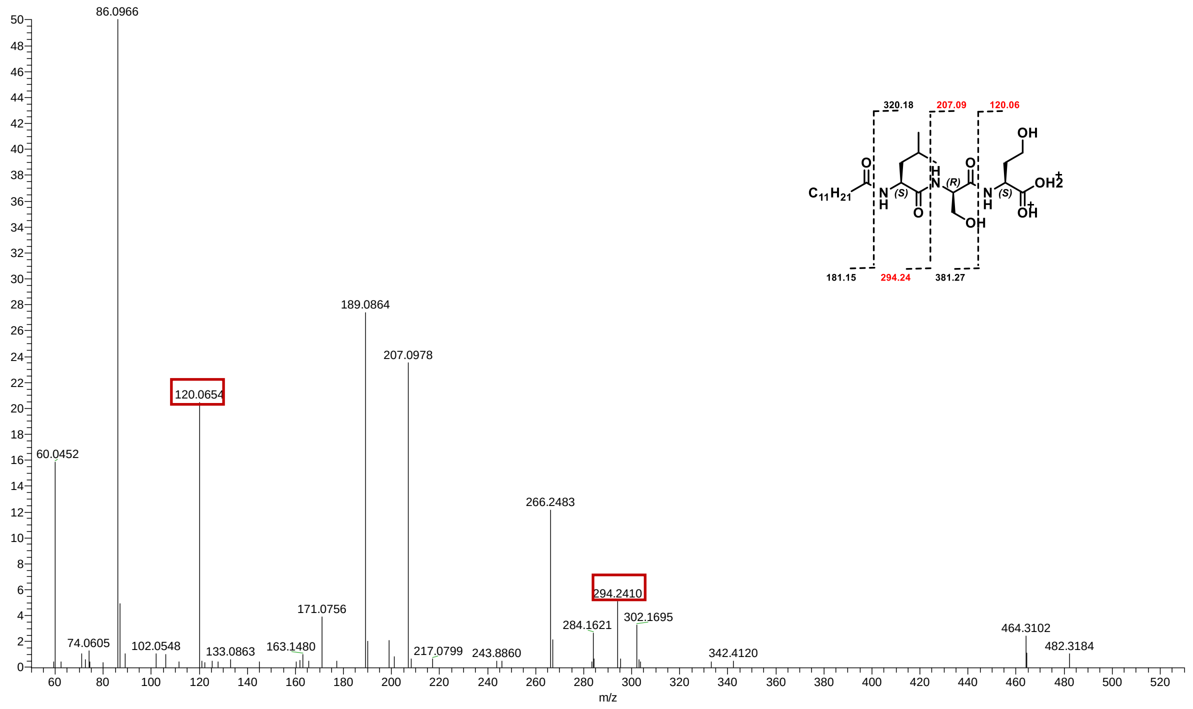

Figure S11. MS^2-fragmentation of F1.

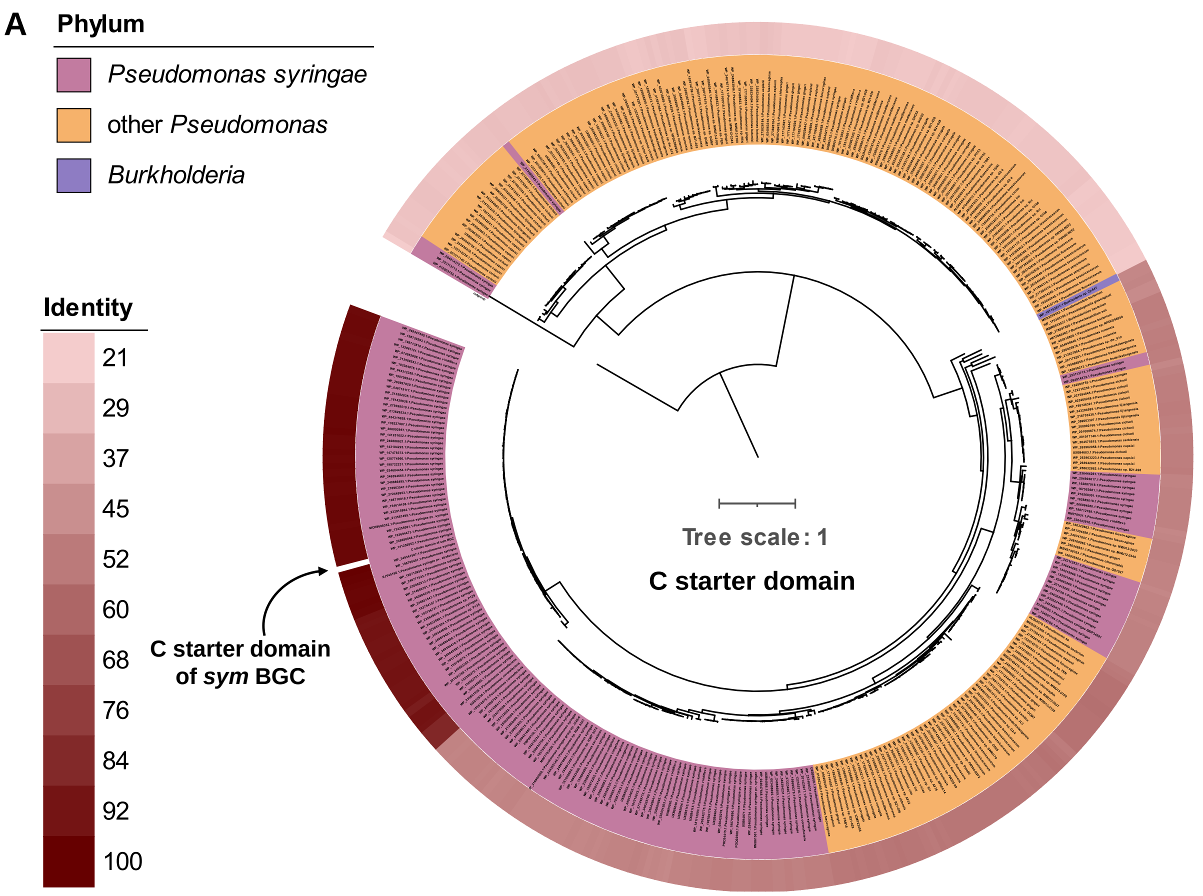
A
Phylum
Pseudomonas syringae
other Pseudomonas
Burkholderia
Identity
21
29
37
45
52
60
68
76
84
92
100
C starter domain of sym BGC
Tree scale: 1
C starter domain

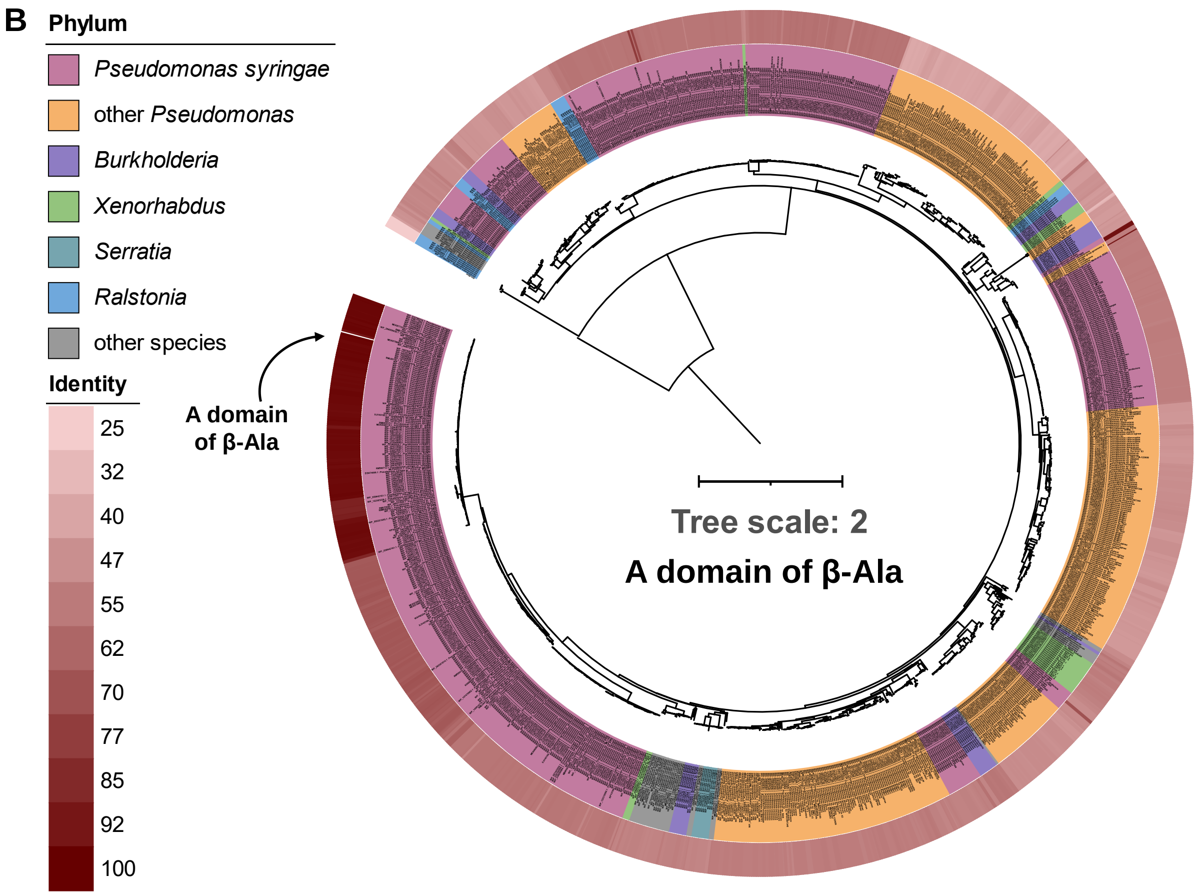
B
Phylum
Pseudomonas syringae
other Pseudomonas
Burkholderia
Xenorhabdus
Serratia
Ralstonia
other species
Identity
25
32
40
47
55
62
70
77
85
92
100
A domain of β-Ala
Tree scale: 2
A domain of β-Ala

C

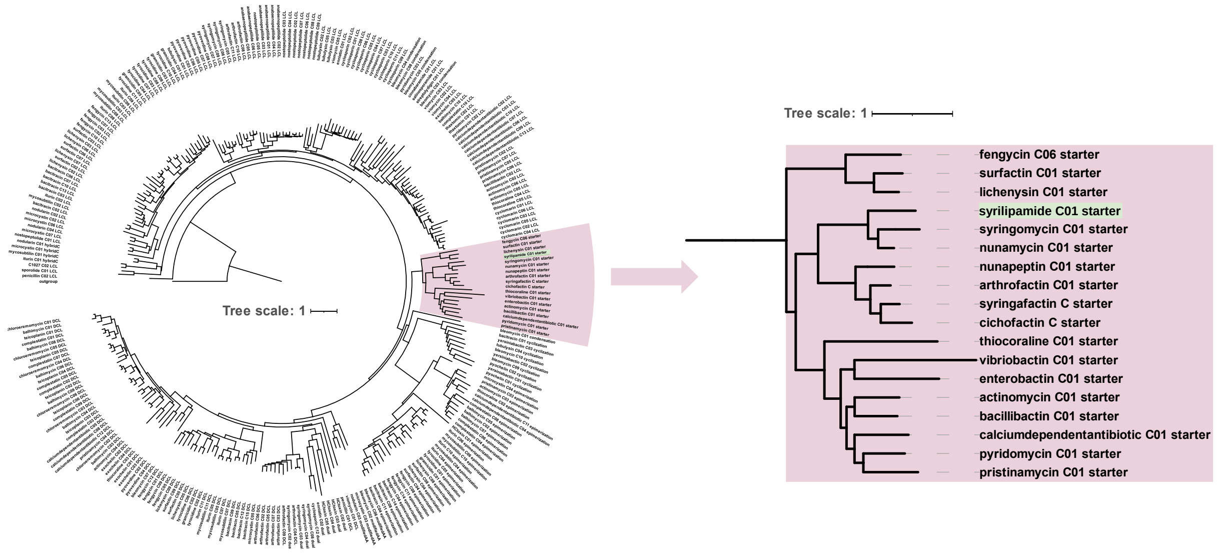

Figure S12. Phylogenetic analysis of $C_{starter}$ and A domain of β-Ala domains of *sym* BGC. (A) The phylogenetic analysis of the *sym* $C_{starter}$ domain shows that homologs thereof are widely distributed within *P. syringae*. (B) Analysis of the A domain of the β-Ala module shows that similar modules are prevalent within the *P. syringae* species as well as across various other *Pseudomonas* species. The color coding in the outermost circle of the phylogenetic tree shows amino acids identity between C starter domain/ A domian of β-Ala and biosynthetic genes or BGC. (C) A phylogenetic analysis containing all C domains listed in the Natural Product Domain Seeker (NaPDoS) database highlights a close relationship between the $C_{starter}$ domain of the syrilipamide NRPS and that of syringomycin.

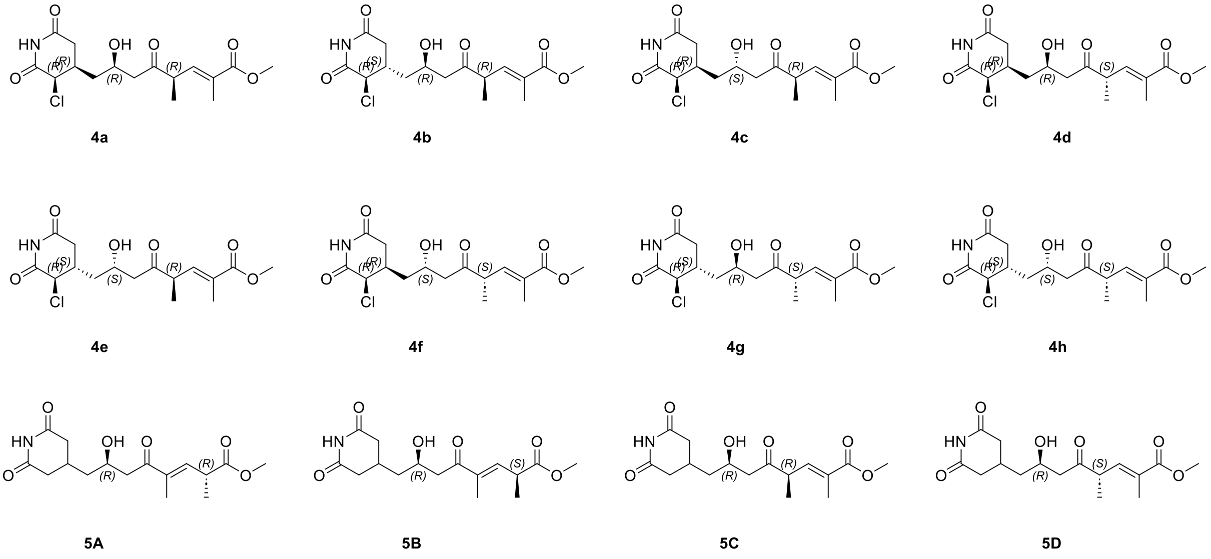

Figure S13. Eight and four stereoisomers illustrating the relative configuration identification of **4** and **5d**, respectively.

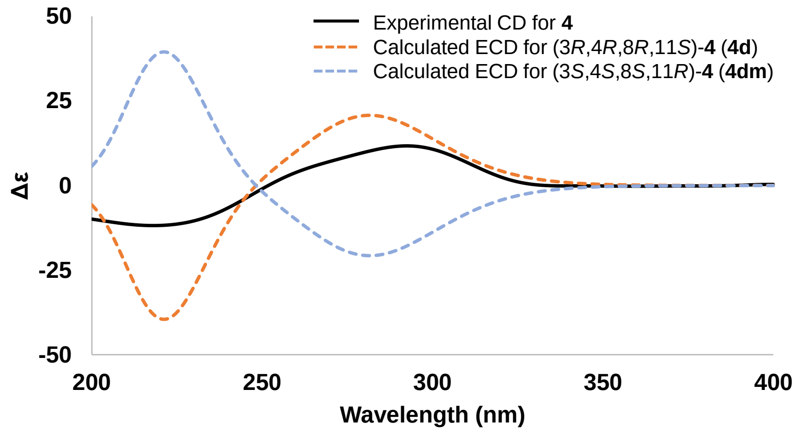

Figure S14. Comparison between the experimental (line) and theoretical (dashed) ECD spectra of the two enantiomers of **4**.

A

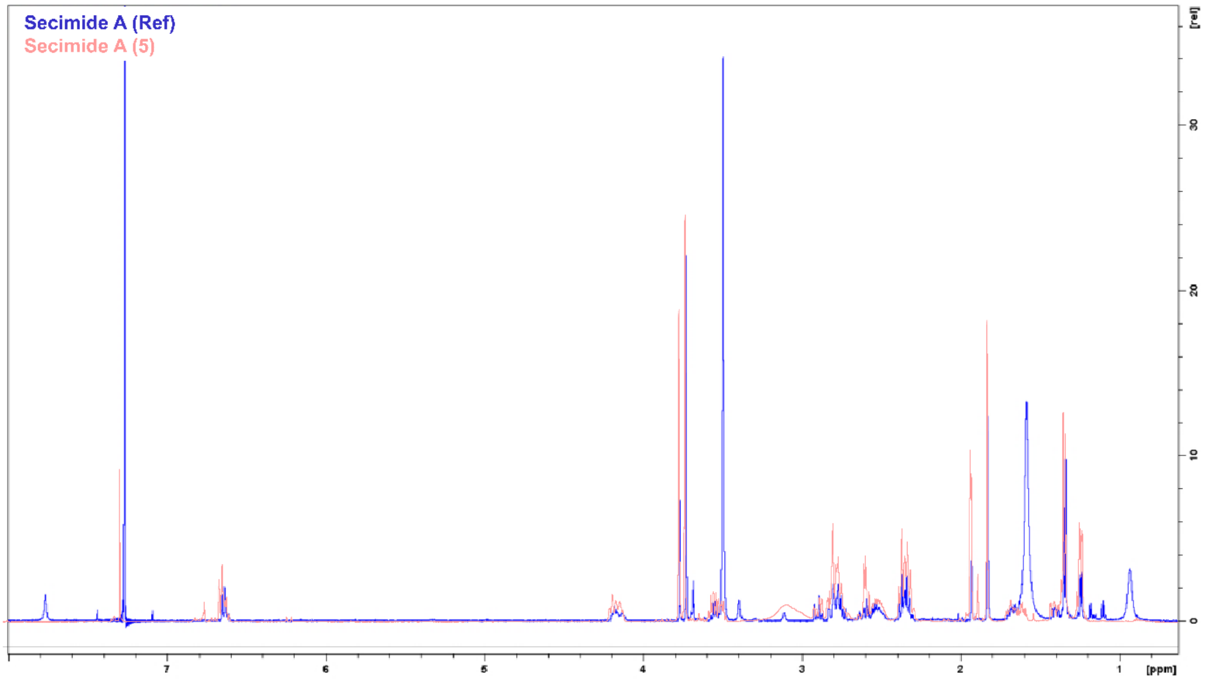

B

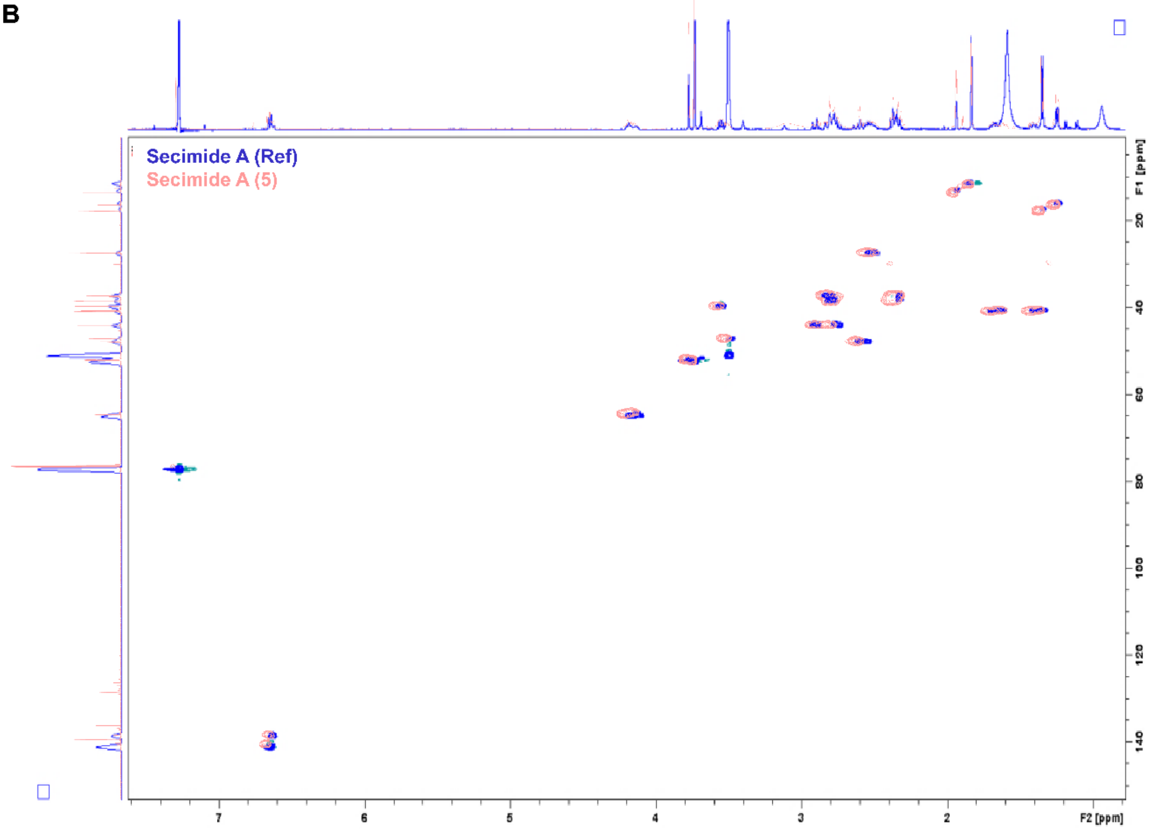

Figure S15. NMR data comparison of reported secimide A and compound **5**. (A) Comparison of the ^{1}H NMR spectra for secimide A and compound **5**. (B) Comparison of HSQC data. These data suggest that the configurations of the two compounds are identical.

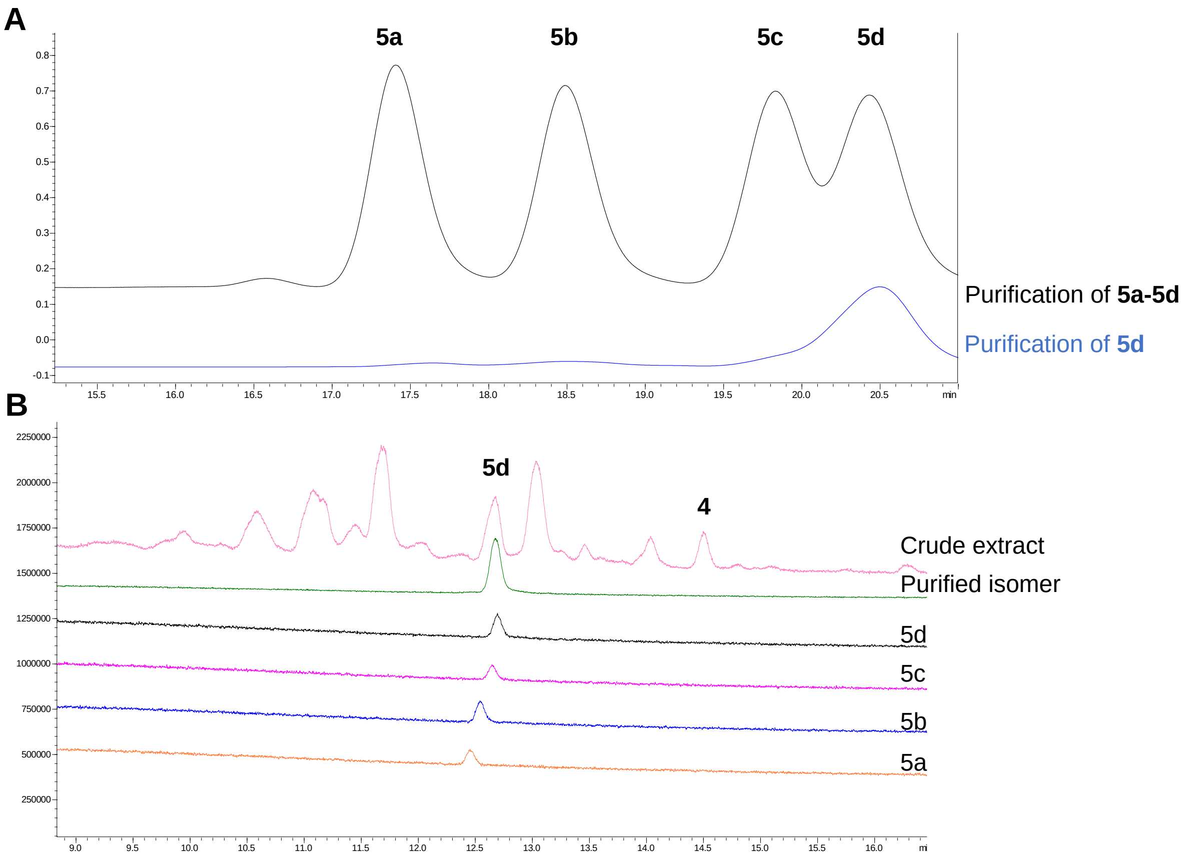

Figure S16. Purification of four secimides A isomers (**5a**-**5d**). (A) HPLC profiles of crude extracts under different conditions: black trace represents the extract from normal growth conditions (22°C incubation, 40°C extraction), while the blue trace represents the extract from cold conditions (5°C incubation, freeze-dried extraction). (B) LCMS profile of the crude extract from cold conditions, demonstrating that the bacteria exclusively produce a single isomer, secimide A (**5d**).

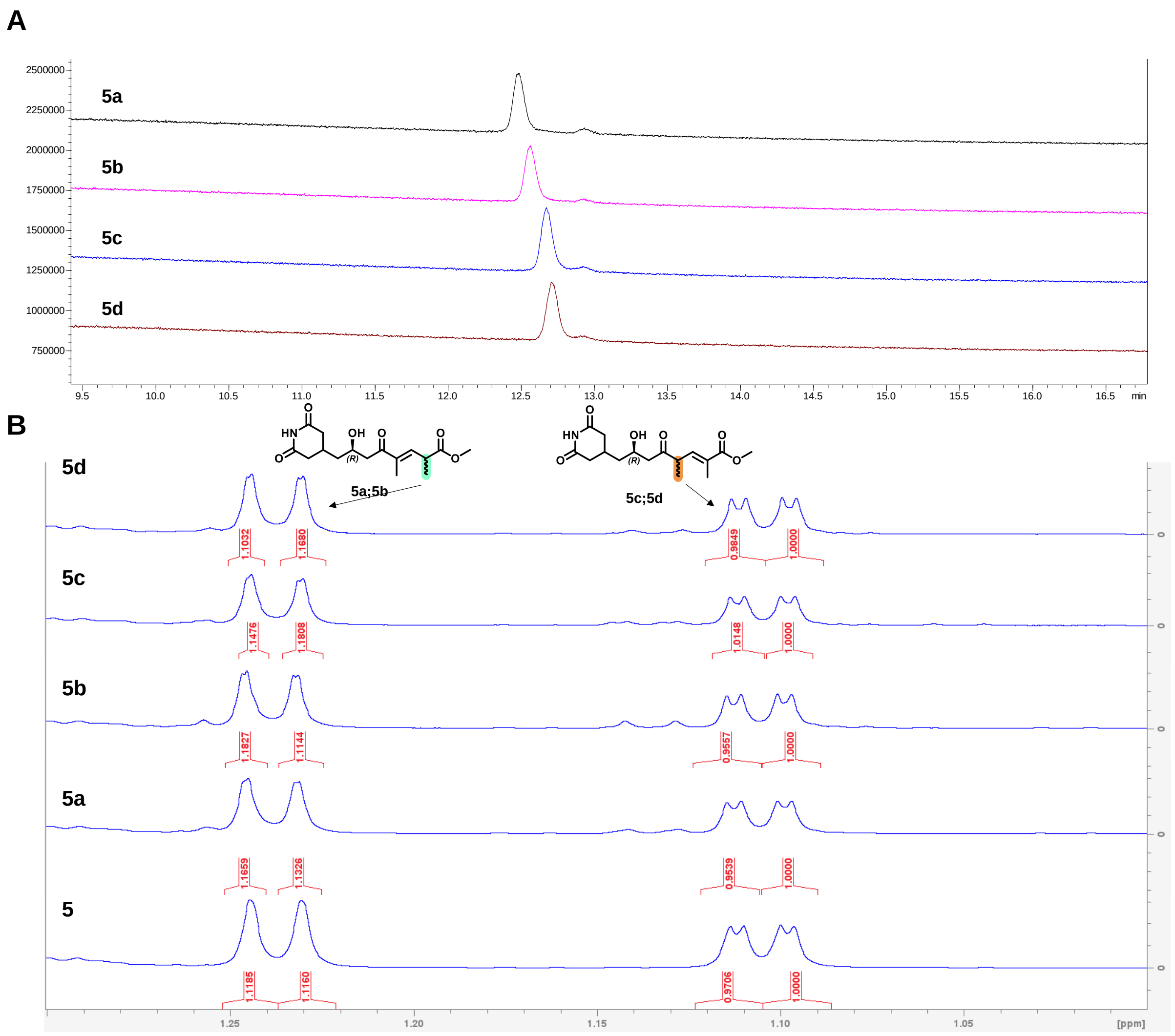

Figure S17. Analysis for four isomers of secimide A. (A) Purified four isomers of secimide A (**5a–5d**). (B) The ^{1}H NMR spectra revealed that the purified secimide A isomers reverted to a mixture of the four isomers either prior to or during the NMR measurement, with an observed ratio of 1:1:1:1.

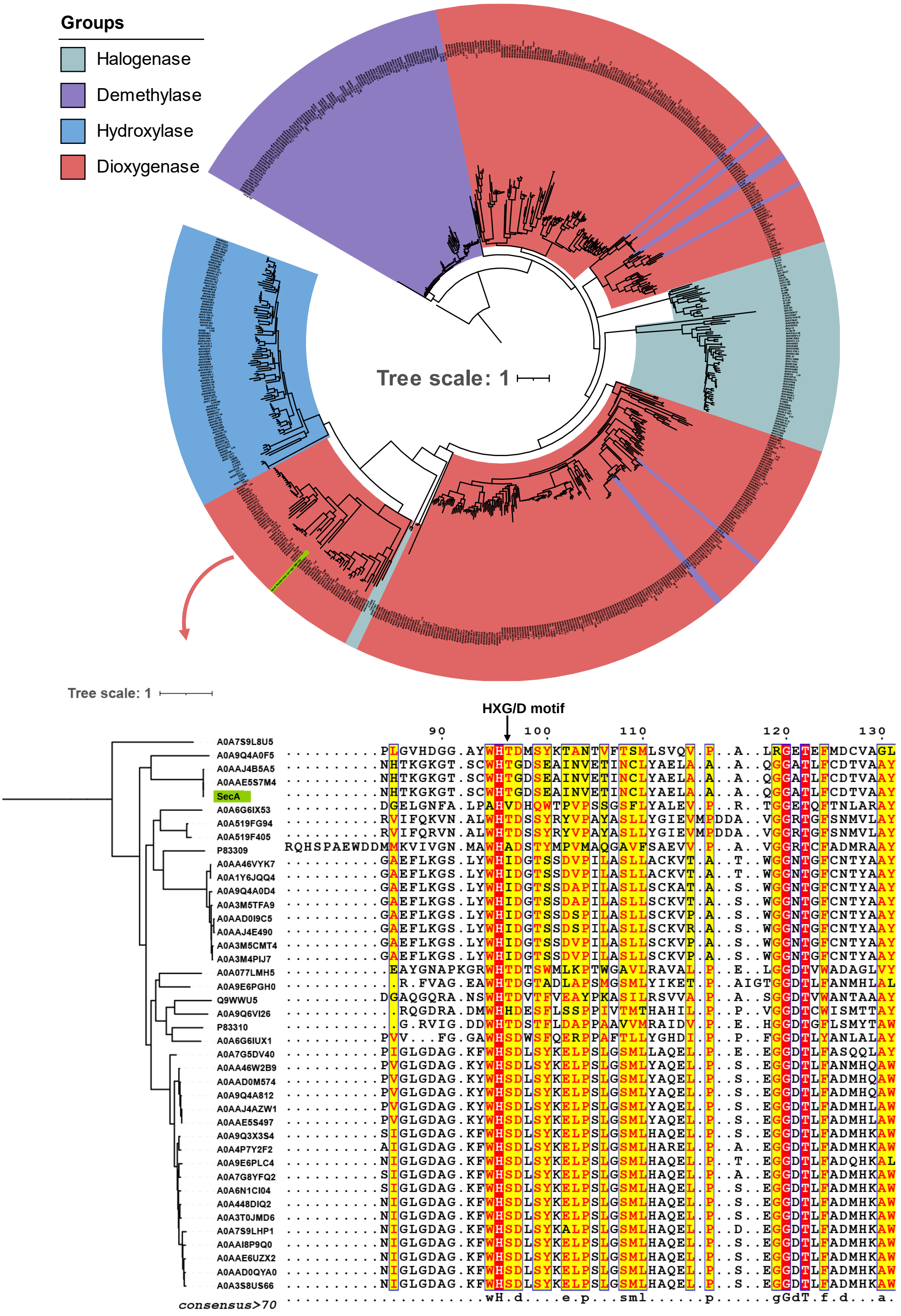

Figure S18. Phylogenetic analysis of α-KG-dependent enzymes. The phylogenetic analysis revealed four distinct groups of α-KG-dependent enzymes, with SecA positioned within the dioxygenase group (top panel). The bottom panel highlights enzymes within the same clade as SecA, which share conserved sequences. Notably, SecA possesses a characteristic halogenase HXG motif, whereas other enzymes in this clade exhibit an HXD motif, typically associated with hydroxylation activity.

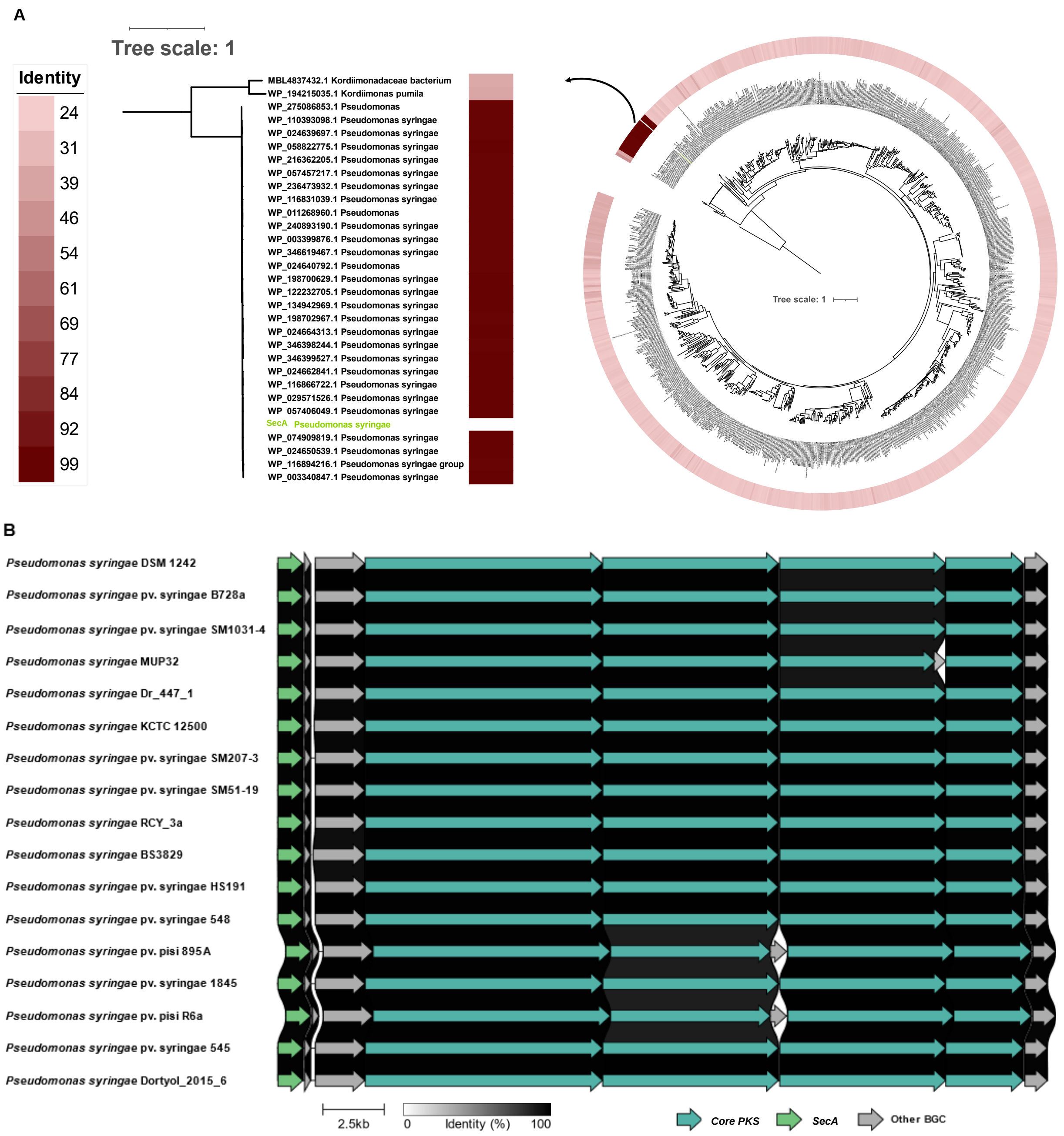

Figure S19. Phylogenetic and BGC analysis related to SecA. (A) Phylogenetic analysis indicates that SecA is widely distributed within the *P. syringae* group, exhibiting high sequence identities ranging from 90% to 100%. In contrast, SecA shows significantly lower identity (below 45%) in other bacterial groups. (B) Analysis of selected *P. syringae* strains from (A) with high sequence identity to SecA reveals that SecA is consistently located near the secimide core PKS genes.

```
T-COFFEE, Version_11.00 (Version_11.00)
Cedric Notredame
SCORE=376
*
 BAD AVG GOOD
*
BesD     :  37
SecA     :  35
SvrB2    :  38
Wel05    :  34
cons     :  37

BesD     GPHMGSNRQE-----------LKD---VCAPLEK----------------------------------------------DDIRRLSQAFHR-
SecA     MGLKLSSVPFEISYPAQSFGVEIENLKLHKNLSF----------------------------------------------DTIAAIKKCLAY-
SyrB2    GSHMSK--------------------------KF----------------------------------------------ALTAEQRASFEK-
Wel05    MKHHHHHHHSDYDIP------TTENLYFQGSMSNNTVSTKPALHFLDINATEVKKYPTAIQDIIINRSFDGMIIRGVFPRDTMEQVARCLEEG

cons                                     .                                                       .:

BesD     --FGIVTVTELIEPHTRKLVRA-EADRLLDQYAERRDLRLATTDYT-----RRSMSVVPSETIAANSELVTGLYAHRELL------APLEAIA
SecA     --RGFVLFR--NQNL-TRREQI-AVTRLLGN-PNLK---LHSWAPQIETATFGDDEVIPNGLP---------LINHGEIL------YF---VN
SyrB2    --NGFIGPFDAYSPEEMKETWKRTRLRLLDR----------------SAAAYQDLDAISGGTN---------IANYDRHLDDDFLASH---IC
Wel05    NDGGMKSILNKNEEFGTKVAQI-YGHAIVGQSPDLK---DYFAS---------------SAIF-----------RQACRT------MF---QG

cons        *:       .    :         ::..                            .

BesD     GERLHPCPKADEEFL----ITRQ----EQRGD-THGWHWGDFSF---------------A----LIWVLQA--PPIDVGGLLQCVPHT--TW-
SecA     GPD--FCDKTQDEHA----IWDEKD-NHTKGKGTSCWHTGDSEA---------------IN-VETINCLYA--ELAAAQGGATLFCDTVAAYN
SyrB2    RPE--ICDRVESILGPNVLCWRTEFFPKYPGDEGTDWHQADTFANASGKPQIIWPENEEFGGTITVWTAFT--DANIANGCLQFIPGTQNSMN
Wel05    SPD--FEEQVESIFHS-----------L-SGLPVEIPTGPEGQT---------------YT-PATIRLLLEGREIAVHVGNDFLLMPA--AN-

cons             :.:.                  *         :                        :             *    .  :  :

BesD     ----------------------DKASPQINRY-----LVENPIDTY-----------------------------------------HFESGD
SecA     DLEDSLKKRIDNLRVVHYFVDPQRTEPVSQPL-----VKTNPITRQKYLYLNYNTMERVEGLSKSESYRLLKFLFDHQIKDQYVYEHFWKQDD
SyrB2    ---------YDETK-------RMTYEPDANNS-----VVKDGVRRGF-FGYDYRQLQIDENW-KPDEA--------------SAVPMQMKAGQ
Wel05    ----HLKTLLDLSDQLSYFIPL-T-VPEAGGELVVYSLEWNPQEASKYAQMQ-EYMDDVEFKIKSNQS--------------QSVAYAPGPGD

cons                               *          :  :                                                  .:

BesD     VYFLRTDTTLHRTIPLRED--TTR-------IILNMTW-AGERDL---------------SRKLAADD--RW----WDNAEVSAARAIKD---
SecA     FLIWNCNGTMHKRGALVSN--HNRIMRRTQAIIPEFTS-YYERDK---------------SLAVTSHDNEDWHPYPFERAELESAGSA-----
SyrB2    FIIFWST-LMHASYPHSGESQEMRMGF-ASRYVPSFVH-VYPDSDHIEEYGGRISLEKYGAVQVIGDE--TPE---YNRLVTHTTRGKKFEAV
Wel05    MLLFNGGRYYHRVSEVIGN--SPRRT------IGGFLAFSKQRDK---------------IYYW--------------------------S

cons     . :       *       :    *        :  :       .
```

Figure S20. Amino acid sequence of SecA aligned with three known Fe(II)/α-KG-dependent halogenases: BesD (PDB: 6NIE), WelO5 (PDB: 5IQS), and SyrB2 (PDB: 2FCU) in the T-Coffee server.[34]

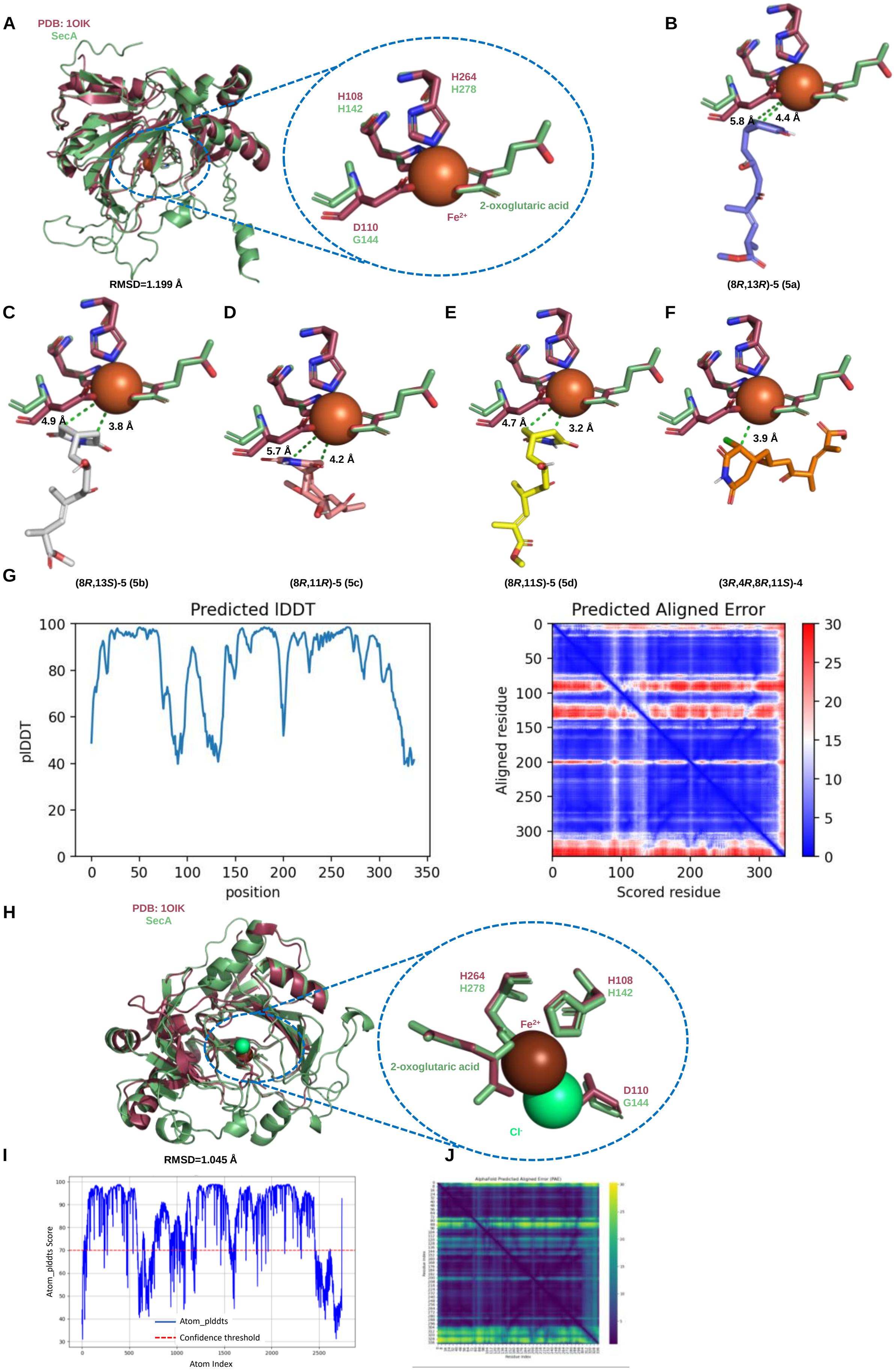
A
PDB: 1OIK
SecA
H264
H278
H108
H142
2-oxoglutaric acid
Fe2+
D110
G144
RMSD=1.199 Å
B
5.8 Å
4.4 Å
(8R,13R)-5 (5a)
C
4.9 Å
3.8 Å
(8R,13S)-5 (5b)
D
5.7 Å
4.2 Å
(8R,11R)-5 (5c)
E
4.7 Å
3.2 Å
(8R,11S)-5 (5d)
F
3.9 Å
(3R,4R,8R,11S)-4
G
Predicted lDDT
plDDT
position
Predicted Aligned Error
Aligned residue
Scored residue
H
PDB: 1OIK
SecA
H264
H278
H108
H142
Fe2+
2-oxoglutaric acid
D110
G144
Cl-
RMSD=1.045 Å
I
Atom_plddts Score
Atom_plddts
Confidence threshold
Atom Index
J

Figure S21. Docking simulation of SecA. (A) Alignment of an AlphaFold2 model of SecA and the crystal structure of Atsk (PDB: 1OIK) containing an iron and 2-oxoglutaric acid complex. Both structures display a high similarity at the catalytical sites. (B) Docking simulation between (8*R*,13*R*) secimide (**5a**) and SecA. (C) Docking simulation between (8*R*,13*S*)- secimide (**5b**) and SecA. (D) Docking simulation between (8*R*,11*R*) secimide (**5c**) and SecA. (E) Docking simulation between (8*R*,11*S*) secimide (**5d**) and SecA. (F) Docking simulation between (3*R*,4*R*,8*R*,11*S*) chlorosecimide and SecA. (G) Predicted LDDT and Predicted Aligned Error of SecA. (H) Alignment of an AlphaFold3 model of SecA and the crystal structure of Atsk (PDB: 1OIK) containing iron and chlorine ions together with 2-oxoglutaric acid complex. Both structures display a high similarity at the catalytical sites. (I) Predicted LDDT of SecA modeled by Alphafold3. (J) Predicted Aligned Error of SecA modeled by Alphafold3.

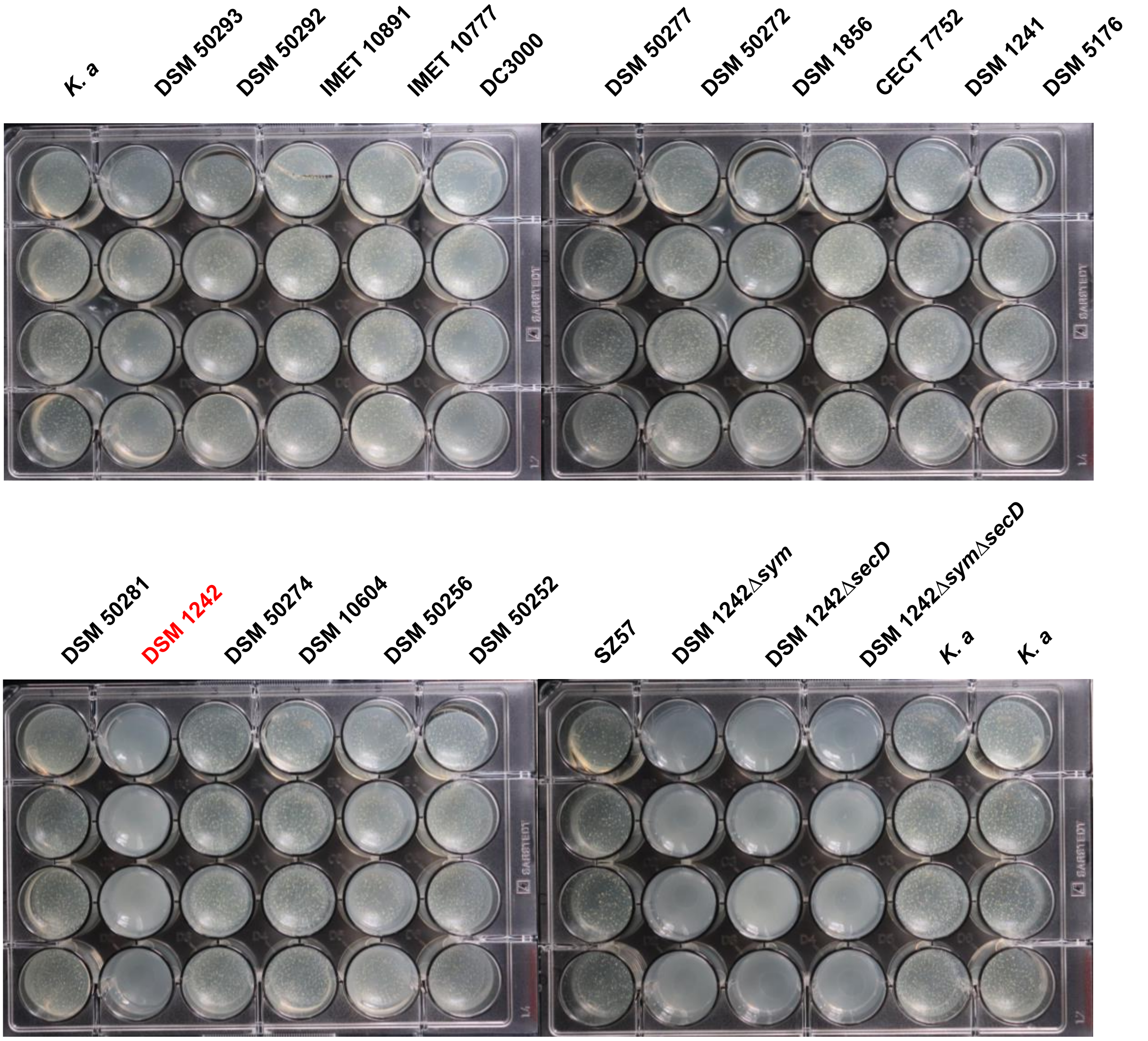

Figure S22. Amoebal plaque assays for all of the *P. syringae* strains in combination with *D. discoideum* AX2.

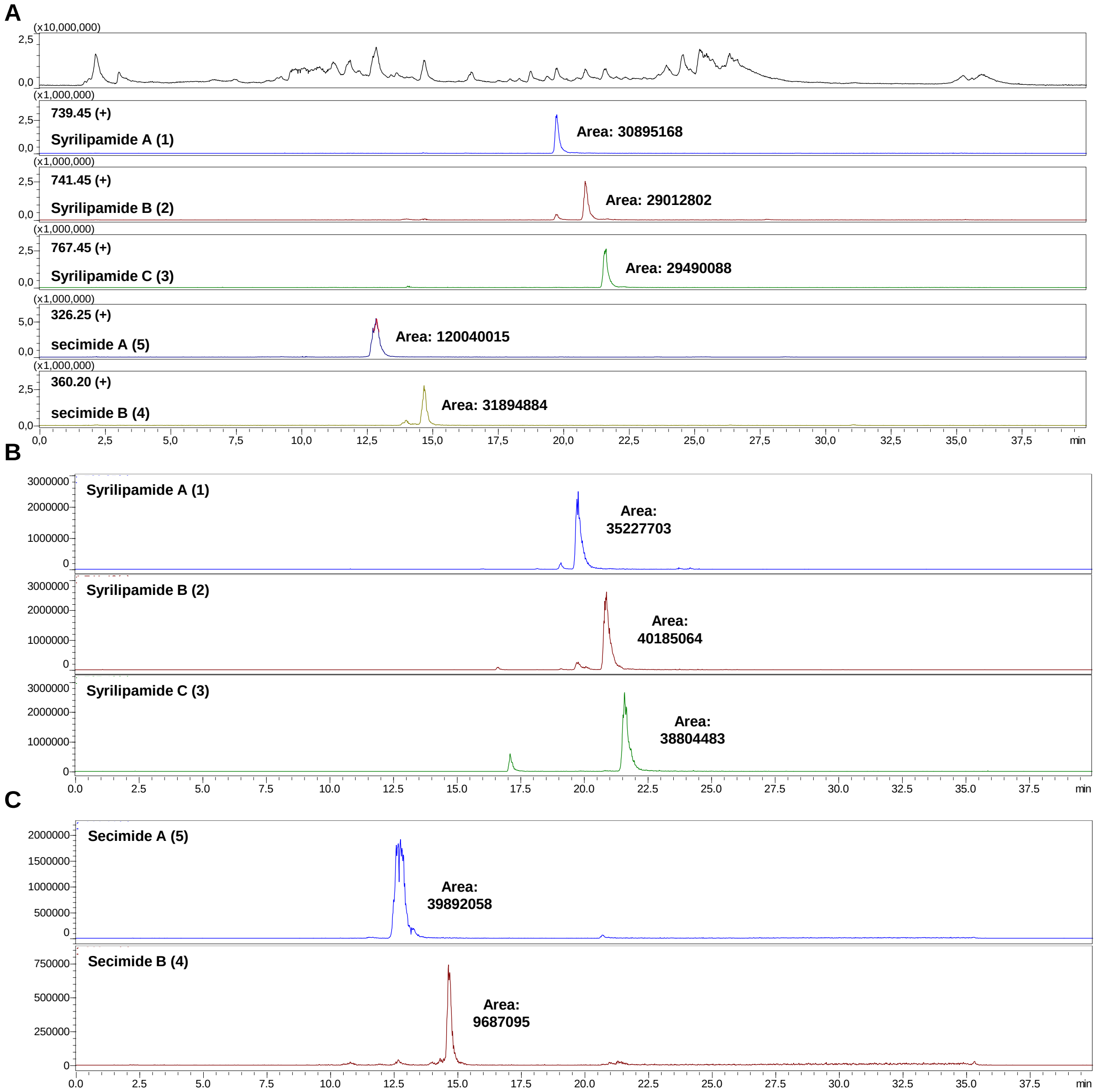

Figure S23. Analysis of ratio of syrilipamides and secimides. (A) The ratio of syrilipamides (1:1:1) and secimides (1:4) in the crude extract, as determined by MS. (B) MS analysis of a prepared mixture of syrilipamides (50 µg:50 µg:50 µg) confirmed the same 1:1:1 ratio. (C) MS analysis of a mixture of secimides (200 µg:50 µg) reproduced the observed 4:1 ratio.

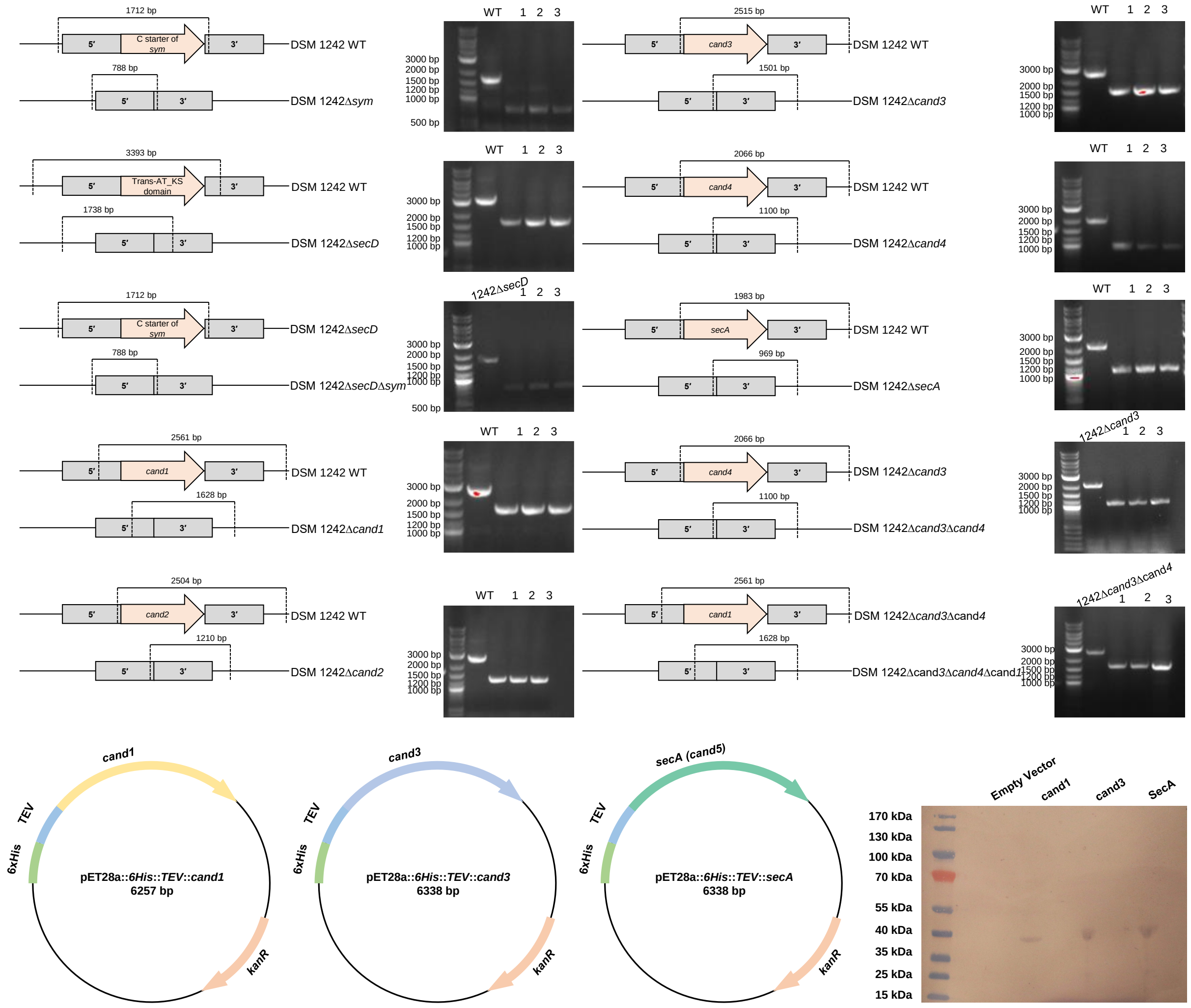

Figure S24. Verification of DSM 1242 mutant strains. Schematics, diagnostic PCRs and Western blots for the generation of the DSM 1242 mutants used in this study. Single-gene deletions were introduced into the DSM 1242 strain through homologous recombination to create seamless mutants. Primers employed for diagnostic PCRs are listed in Table S15. Western blot analyses confirmed the expression of specific proteins in the mutants, with observed molecular weights as follows: Cand1 (38.59 kDa), Cand3 (41.10 kDa), and SecA (42.06 kDa).

Supplementary Tables

Table S1. Results of Marfey's analysis for syrilipamide A (**1**).

Peak#/amino acid	t_R/[min]	Area (detected at λ = 330 nm)
1/L-Ser	9.217	1761787
2/D-Ser	9.503	1989174
3/ L-Home-Ser	9.690	708662
4/ β-Ala	13.440	960075
5/ L-Val	20.150	1432304
6/ L-Leu	25.056	2501897
Result: 1× L-Ser, 1× D-Ser, 1× L- Hse, 1× β-Ala, 1× L-Val, 1× L-Leu		

Table S2. Results of Marfey's analysis for syrilipamide B (**2**).

Peak#/amino acid	t_R/[min]	Area (detected at λ = 330 nm)
1/L-Ser	9.207	5159213
2/D-Ser	9.493	5726057
3/ L-Home-Ser	9.681	1900199
4/ β-Ala	13.426	2612011
5/ L-Val	20.136	3820802
6/ L-Leu	25.047	5367786
Result: 1× L-Ser, 1× D-Ser, 1× L-Hse, 1× β-Ala, 1× L-Val, 1× L-Leu		

Table S3. Results of Marfey's analysis for syrilipamide C (**3**).

Peak#/amino acid	t_R/[min]	Area (detected at λ = 330 nm)
1/L-Ser	9.200	3233912
2/D-Ser	9.486	3590408
3/ L-Home-Ser	9.673	1787958
4/ β-Ala	13.428	1752619
5/ L-Val	20.129	2355188
6/ L-Leu	25.040	3080630
Result: 1× L-Ser, 1× D-Ser, 1× L- Hse, 1× β-Ala, 1× L-Val, 1× L-Leu		

Table S4. Results of Marfey's analysis for fragment F1.

Peak#/amino acid	t_R/[min]	Area (detected at λ = 330 nm)
1/D-Ser	9.477	2191925
2/ L-Home-Ser	9.648	1924784
3/ L-Leu	24.984	2883814
Result: 1× D-Ser, 1× L- Hse, 1× L-Leu		

Table S5.^{1}H NMR (600MHz) data and ^{13}C NMR (150 MHz) of compounds **1−3** (*δ* in ppm, *J* in Hz)

	Syrilipamide A (**1**)[a]		Syrilipamide B (**2**)[a]		Syrilipamide C (**3**)[a]	
1	-NH	8.505 (d, 5.10)	-NH	8.504 (d, 4.8)	-NH	8.496 (d, 5.10)
2	52.77	4.141 (m)	52.82	4.127 (m)	52.81	4.136 (m)
3	172.61		172.61		172.6	
4	39.44	1.522;1.459 (m)	39.36	1.524;1.4463 (m)	39.37	1.522;1.435 (m)
5	24.08	1.649 (m)	24.07	1.659 (m)	24.07	1.661 (m)
6	22.67	0.913 (d, 6.6)	22.65	0.914 (d, 6.5)	22.65	0.919 (d, 6.6)
7	21.91	0.873 (d, 6.7)	21.93	0.868 (d, 6.6)	21.93	0.872 (d, 6.5)
8	-NH	8.336 (d, 8.9)	-NH	8.299 (d, 8.9)	-NH	8.287 (d, 8.9)
9	62.92	4.418 (m)	62.87	4.43 (m)	62.87	4.43 (m)
10	168.75		168.65		168.66	
11	51.42	4.572 (ddd, 9.0, 4.4, 2.1)	51.40	4.573 (ddd, 9.0, 4.4, 2.1)	51.39	4.58 (ddd, 9.0, 4.4, 2.0)
12	-NH	7.947 (d, 8.1)	-NH	7.919 (d, 8.7)	-NH	7.902 (d, 8.6)
13	52.14	4.326 (td, 9.0, 5.3)	52.15	4.343 (td, 9.1, 5.3)	52.15	4.343 (td, 9.0, 5.3)
14	171.41		171.40		171.40	
15	35.19	1.820;1.766 (m)	35.25	1.819;1.750 (m)	35.22	1.822;1.761 (m)
16	57.16	3.379;3.319 (m)	57.15	3.380;3.312 (m)	57.15	3.383;3.318 (m)
17	-OH		-OH		-OH	
18	-NH	7.557 (d, 8.6)	-NH	7.572 (d, 8.6)	-NH	7.558 (d, 8.6)
19	58.08	4.052 (dd, 8.5, 6.8)	58.02	4.06 (dd, 8.7, 6.8)	58.00	4.061 (dd, 8.7, 6.9)
20	170.78		170.76		170.76	
21	30.03	2.066 (q, 6.8)	30.06	2.06 (q, 6.8)	30.06	2.06 (q, 6.8)
22	19.19	0.829 (d, 6.8)	19.17	0.823 (d, 6.6)	19.17	0.831 (d, 6.5)
23	17.92	0.812 (6.8)	17.90	0.806 (d, 6.7)	17.89	0.813 (d, 6.6)
24	-NH	7.788 (t, 5.8)	-NH	7.792 (t, 5.8)	-NH	7.795 (t, 5.8)
25	34.82	3.435;3.145 (m)	34.84	3.422;3.153 (m)	34.81	3.429;3.153 (m)
26	34.99	2.463;2.241 (ddd, 15.6, 8.5, 2.7)	34.93	2.467;2.255 (ddd, 15.7, 8.4, 2.8)	34.95	2.476;2.253 (ddd, 15.7, 8.4, 2.8)
27	171.45		171.45		171.44	
28	-NH	8.395 (d, 6.30)	-NH	8.399 (d, 6.2)	-NH	8.386 (d, 6.2)
29	55.42	4.17 (m)	55.41	4.16 (m)	55.40	4.166 (m)
30	170.04		170.01		170.01	
31	60.87	3.67 (dd, 5.4, 2.6)	60.83	3.668 (dd, 5.4, 2.2)	60.83	3.671 (dd, 5.5, 2.5)
1'	174.11		174.38		174.32	
2'	34.50	2.177 (m)	34.78	2.163 (m)	34.78	2.158 (m)
3'	25.40	1.547 (m)	25.24	1.492 (m)	25.15	1.498 (m)
4'	26.18	1.978 (m)	28.49	1.234 (m)	28.19	1.256 (m)
5'	128.80	5.314 (m)[a]/ 5.24 (dt, 9.9, 7.7)[b]	28.90	1.234 (m)	28.29	1.256 (m)
6'	130.25	5.348 (m)[a]/ 5.42 (dt, 9.9, 7.7)[b]	29.10	1.234 (m)	26.54	1.969 (m)
7'	26.64	1.978 (m)	28.99	1.234 (m)	129.48	5.322 (m)[a]/ 5.322 (dt, 10.5, 7.6)[b]
8'	29.11	1.294 (m)	29.01	1.234 (m)	129.70	5.322 (m)[a]/ 5.377 (dt, 10.5, 7.6)[b]
9'	28.34	1.257 (m)	28.72	1.234 (m)	26.60	1.969 (m)
10'	31.16	1.248 (m)	31.29	1.235 (m)	29.09	1.306 (m)
11'	22.10	1.257 (m)	22.10	1.258 (m)	28.8889	1.229 (m)
12'	13.95	0.856 (t, 7.1)	13.96	0.859 (t, 7.2)	31.13	1.245 (m)
13'					22.08	1.261 (m)
14'					13.94	0.856 (t, 7.2)

[a] measured in DMSO-d_6; [b] measured in chloroform-*d*

Table S6. The DP4+ probabilities of **4a**-**4h**.

Functional			Solvent		Basis Set			Type of Data		
mPW1PW91			PCM		6-311+G(d,p)			Shielding Tensors		
		DP4+	1.55%	0.00%	0.00%	98.24%	0.18%	0.03%	0.00%	0.00%
Nuclei	Sp2?	Exptl (**4**)	**4a**	**4b**	**4c**	**4d**	**4e**	**4f**	**4g**	**4h**
C		51.85	133.483104	133.543386	133.618253	133.471110	133.540089	133.533870	133.553254	133.477060
C	x	167.38	11.880247	12.297039	12.568603	12.240937	12.058320	12.341539	12.093233	12.069999
C	x	128.61	47.857314	48.190064	46.067931	48.146185	48.505977	48.128198	48.304720	48.195744
C		12.71	172.109332	172.025958	172.226779	171.974432	172.101889	171.841596	171.994212	172.050302
C	x	140.31	38.041173	38.300558	39.238811	37.712023	37.470727	38.590572	37.685716	38.239980
C		46.03	132.534222	132.250757	132.609291	133.093745	133.561397	133.745299	133.469283	134.266609
C		15.71	171.407902	172.143647	170.174140	172.238310	172.513766	171.554008	172.355932	171.669483
C	x	208.50	-41.294757	-41.282594	-40.880077	-39.709318	-39.162192	-39.787308	-39.400402	-38.762880
C		48.91	139.635018	139.092638	140.332636	137.720596	137.327465	138.235362	137.249069	140.040577
C		63.49	119.176813	116.828781	117.960695	120.116478	119.227304	120.211994	114.659565	118.140023
C		37.51	150.092770	149.506086	148.157220	149.250804	147.636725	148.366977	147.978093	147.542729
C		31.33	152.028298	147.626801	151.597618	152.574951	148.223589	152.672528	144.230074	147.854147
C		33.36	151.432220	153.668574	153.448576	151.402308	151.521080	154.005204	149.530541	151.066362
C	x	171.81	8.975851	9.264991	8.930140	8.953765	8.592526	8.821956	9.112600	8.658087
C	x	168.92	12.178219	12.266572	12.238648	12.130106	12.448694	12.385002	11.918885	12.709782
C		58.06	121.945570	121.920144	118.347832	121.999771	122.267219	118.198244	123.687405	123.203162
H		3.68	28.024062	28.031711	28.028678	28.032384	28.044549	28.032756	28.050994	28.045202
H		3.68	28.031105	28.052725	28.061702	28.029084	28.039958	28.031104	28.043495	28.046746
H		3.68	28.025155	28.034008	28.016330	28.034217	28.041660	28.049243	28.050419	28.044421
H		1.87	29.648281	29.661689	29.916166	29.609667	29.889877	29.910305	29.743559	29.732809
H		1.87	29.919621	29.897158	29.886482	29.769768	29.744957	29.638602	29.920290	29.868992
H		1.87	29.778828	29.749245	30.082195	29.862609	29.615753	29.756210	29.589052	29.575271
H	x	6.57	24.739368	24.894334	24.677932	24.845410	24.830930	24.887715	24.866187	24.929946
H		3.65	28.068125	28.053266	28.250108	27.949205	28.011857	27.921046	28.000803	27.917453
H		1.11	30.412181	30.429474	30.546369	30.411910	30.937888	30.615871	30.387077	30.624717
H		1.11	30.749014	30.901630	30.339677	30.883578	30.580997	30.788035	30.894150	30.820087
H		1.11	30.476830	30.565047	30.261162	30.509414	30.384847	30.231278	30.559970	30.230382
H		2.57	29.027066	29.065487	29.329326	29.045069	29.547043	29.594239	29.128941	29.458990
H		2.52	29.233371	29.150299	29.146132	29.449631	29.126798	28.959705	29.516751	29.009108
H		4.00	27.923538	27.975146	28.016491	27.792660	27.859348	27.902599	27.628762	27.850457
H		4.88	27.581915	27.627765	27.413273	27.469816	27.411728	27.273167	27.166946	27.572168
H		1.39	30.307503	30.597726	30.267355	30.440941	30.534000	30.351412	30.507978	30.287723
H		1.53	30.217524	30.293018	30.267741	30.392188	30.210190	30.256526	30.525043	30.162346
H		2.71	28.998563	29.067371	28.964438	29.017384	29.126240	28.919405	29.298262	29.222648
H		2.51	29.095659	28.590071	29.224812	29.150531	28.554881	29.271570	28.495054	28.534573
H		2.42	29.467923	29.302034	29.226432	29.494984	29.289740	29.155828	29.495944	29.550291

Table S7. Experimental CD data and calculated ECD data at the CAM-B3LYP/6-311+G(d,p) level in methanol with the PCM model.

Experimental CD of **4** vs Calculated ECD of (3*R*,4*R*,8*R*,11*S*)-**4** (**4d**)											
Wavelength (nm)	Exptl CD	Calcd ECD	Wavelength (nm)	Exptl CD	Calcd ECD	Wavelength (nm)	Exptl CD	Calcd ECD	Wavelength (nm)	Exptl CD	Calcd ECD
200	-9.9391	-5.71009	250	-0.98267	1.767924	300	10.75219	13.77622	350	-0.13295	0.327084
201	-10.0824	-6.80083	251	-0.42162	2.6987	301	10.4757	13.2004	351	-0.14153	0.296662
202	-10.2306	-8.05017	252	0.12766	3.594532	302	10.1681	12.6256	352	-0.15056	0.268917
203	-10.3816	-9.46655	253	0.66295	4.461384	303	9.83131	12.05431	353	-0.15956	0.243631
204	-10.5335	-11.0522	254	1.18235	5.304659	304	9.46758	11.4888	354	-0.16808	0.220603
205	-10.6845	-12.8028	255	1.68432	6.129097	305	9.07946	10.93115	355	-0.17567	0.199647
206	-10.8325	-14.7066	256	2.16771	6.938694	306	8.66979	10.38323	356	-0.18195	0.180588
207	-10.9758	-16.7451	257	2.63177	7.736652	307	8.24163	9.846693	357	-0.18661	0.163267
208	-11.1126	-18.8928	258	3.07615	8.525345	308	7.79824	9.322992	358	-0.18941	0.147535
209	-11.2414	-21.118	259	3.50085	9.306312	309	7.34305	8.813387	359	-0.19022	0.133255
210	-11.3607	-23.3839	260	3.90627	10.08027	310	6.87958	8.318941	360	-0.18902	0.120301
211	-11.4692	-25.6498	261	4.29311	10.84714	311	6.4114	7.840538	361	-0.18588	0.108558
212	-11.5659	-27.8723	262	4.66237	11.60611	312	5.9421	7.378886	362	-0.18098	0.097917
213	-11.6497	-30.0073	263	5.0153	12.35567	313	5.47521	6.934532	363	-0.17458	0.088281
214	-11.7198	-32.011	264	5.35333	13.09371	314	5.01418	6.507872	364	-0.16702	0.07956
215	-11.7754	-33.8422	265	5.67804	13.81759	315	4.56229	6.099158	365	-0.15869	0.071672
216	-11.8161	-35.4634	266	5.99109	14.52424	316	4.12266	5.708518	366	-0.15	0.064539
217	-11.841	-36.8421	267	6.29416	15.21024	317	3.69813	5.335958	367	-0.14139	0.058094
218	-11.8499	-37.9517	268	6.5889	15.87194	318	3.29131	4.981384	368	-0.13326	0.052273
219	-11.842	-38.7726	269	6.87688	16.50554	319	2.90448	4.644604	369	-0.12598	0.047018
220	-11.8169	-39.2923	270	7.15952	17.1072	320	2.53959	4.325345	370	-0.11986	0.042276
221	-11.774	-39.5057	271	7.43807	17.67312	321	2.19824	4.023261	371	-0.1151	0.037999
222	-11.7126	-39.4148	272	7.71354	18.19962	322	1.88165	3.737945	372	-0.11183	0.034143
223	-11.632	-39.0285	273	7.98671	18.68325	323	1.59068	3.468937	373	-0.11004	0.030668
224	-11.5314	-38.3614	274	8.25806	19.12084	324	1.32579	3.215732	374	-0.10962	0.027538
225	-11.41	-37.4338	275	8.52777	19.50956	325	1.08708	2.977792	375	-0.11034	0.02472
226	-11.2669	-36.2698	276	8.79571	19.84697	326	0.87431	2.754551	376	-0.11182	0.022184
227	-11.1011	-34.8969	277	9.06145	20.13107	327	0.68687	2.54542	377	-0.11363	0.019902
228	-10.9117	-33.3448	278	9.32422	20.36033	328	0.52387	2.349801	378	-0.11522	0.017849
229	-10.6978	-31.6444	279	9.58299	20.5337	329	0.38412	2.167082	379	-0.11598	0.016004
230	-10.4584	-29.8266	280	9.83641	20.65063	330	0.26619	1.996652	380	-0.11529	0.014346
231	-10.193	-27.9219	281	10.0829	20.71106	331	0.16846	1.8379	381	-0.11249	0.012856
232	-9.90074	-25.9593	282	10.32066	20.71543	332	0.08913	1.690219	382	-0.10699	0.011518
233	-9.58135	-23.9656	283	10.54767	20.66462	333	0.02631	1.553014	383	-0.09824	0.010317
234	-9.23462	-21.9657	284	10.76177	20.55998	334	-0.02199	1.425698	384	-0.08579	0.009238
235	-8.86062	-19.981	285	10.96071	20.40326	335	-0.05776	1.307702	385	-0.06934	0.008271
236	-8.45975	-18.0305	286	11.14214	20.19658	336	-0.083	1.198472	386	-0.04871	0.007403
237	-8.0327	-16.1297	287	11.30371	19.94241	337	-0.09963	1.09747	387	-0.02395	0.006625
238	-7.58052	-14.2912	288	11.44307	19.6435	338	-0.10947	1.004181	388	0.00473	0.005927
239	-7.10459	-12.5245	289	11.55797	19.30286	339	-0.11423	0.918108	389	0.03689	0.005302
240	-6.60665	-10.8364	290	11.64626	18.92369	340	-0.11546	0.838775	390	0.07188	0.004742
241	-6.08874	-9.23084	291	11.70594	18.50936	341	-0.11451	0.76573	391	0.10887	0.00424
242	-5.55322	-7.7096	292	11.73522	18.06334	342	-0.11256	0.69854	392	0.14679	0.003791
243	-5.00272	-6.2723	293	11.73254	17.58918	343	-0.11055	0.636796	393	0.18444	0.003388
244	-4.44006	-4.91679	294	11.69662	17.09044	344	-0.10924	0.580109	394	0.22047	0.003028
245	-3.86828	-3.63945	295	11.62649	16.57069	345	-0.10917	0.528112	395	0.25341	0.002706
246	-3.29049	-2.43548	296	11.52148	16.03343	346	-0.11067	0.48046	396	0.28175	0.002417
247	-2.7099	-1.29919	297	11.38129	15.4821	347	-0.11388	0.436827	397	0.30395	0.002159
248	-2.12969	-0.22431	298	11.206	14.92002	348	-0.1188	0.396908	398	0.31851	0.001929
249	-1.55295	0.795809	299	10.99602	14.35038	349	-0.12525	0.360416	399	0.32399	0.001722

Table S8. ^{1}H NMR (500MHz) data and ^{13}C NMR (125 MHz) of compounds **4** and **5** (δ in ppm, J in Hz)

	Secimide B (**4**)[a]		Secimide A (**5a-5d**)[a]		Secimide A (**5d**)[a]	
1	-NH	11.168 (s)	-NH	10.681 (s)	-NH	10.678 (s)
2	168.92		173.41; 173.32; 173.39; 173.30		173.39	
3	58.06	4.726 (d, 2.50)	36.88; 36.86; 38.00; 38.04	2.494; 2.272 (m); 2.553; 2.239 (m)	38.00	2.553; 2.239 (m)
4	31.33	2.71 (m)	26.63; 26.63; 26.64; 26.60	2.236 (m)	26.64	2.236 (m)
5	33.36	2.508;2.424 (m)	36.88; 36.86; 36.87; 36.84	2.494; 2.272 (m); 2.553; 2.239 (m)	36.84	2.553; 2.239 (m)
6	171.81		173.41; 173.32; 173.39; 173.30		173.30	
7	37.51	1.534;1.387 (dq, 14.12, 3.63)	41.67; 41.64; 41.70; 41.65	1.340 (m)	41.65	1.340 (m)
8	63.49	3.998 (m)	64.75; 64.55; 63.83; 63.71	3.990 (m); 3.958 (m)	63.71	3.958 (m)
9	48.91	2.572;2.518 (dd, 7.37, 5.10)	45.50; 45.48; 49.06; 49.03	2.834; 2.657 (m); 2.536; 2.570 (m)	49.06	2.536; 2.570 (m)
10	208.50		200.29; 200.19; 208.78; 208.73		208.73	
11	46.03	3.654 (m)	137.63; 137.57; 46.17; 46.05	3.632 (m), 3.648 (m)	46.05	3.632 (m)
12	140.31	6.574 (dd, 10.1, 1.25)	140.26; 140.22; 140.35; 140.25	6.625 (m) 6.573 (m)	140.35	6.573 (m)
13	128.61		38.91; 38.91; 128.55; 128.51	3.602 (m)	128.55	
14	167.38		173.55; 173.53; 167.39; 167.39		167.39	
15	15.71	1.110 (d, 7.06)	11.38; 11.34; 15.75; 15.70	1.732 (s); 1.10 (d,7.10))	15.70	1.10 (d,7.10)
16	12.71	1.869 (d, 1.25)	16.84;16.84; 12.70; 12.68	1.24 (d, 7.08); 1.860 (d, 1.25)	12.68	1.860 (d, 1.25)
17	51.85	3.675 (s)	52.03; 52.03; 52.03; 51.83	3.641 (s); 3.677 (s)	51.83	3.677 (s)
18	-OH	4.88 (d, 5.73)	-OH	4.730 (s); 4.761 (d; 5.78)	-OH	4.761 (d, 5.78)

[a] measured in DMSO-d_6

Table S9. The DP4+ probabilities of **5d**.

Functional			Solvent	Basis Set		Type of Data
mPW1PW91			PCM	6-311+G(d,p)		Shielding Tensors
		DP4+	0.00%	0.00%	0.00%	100%
Nuclei	Sp2?	Exptl (**5d**)	**5A (8*R*,13*R*)**	**5B (8*R*,13*S*)**	**5C (8*R*,11*R*)**	**5D (8*R*,11S)**
C		51.83	132.9471068	132.2266	132.4479735	133.277814
C	x	167.39	3.128608998	0.2397	9.340754957	11.45823315
C	x	128.55	143.4613361	143.1601	47.63809846	48.17583052
C		12.68	166.6589691	168.1751	170.4475764	172.310085
C	x	140.35	32.25030891	31.7267	39.04713332	37.19997879
C		46.05	43.53462943	40.7706	132.8970477	133.0248337
C		15.7	174.1225533	173.4986	172.1558072	172.7500895
C	x	208.73	-26.9822495	-23.1974	-37.7310494	-40.1099887
C		49.06	142.0303084	134.1949	136.0148395	137.6740179
C		63.71	120.4383047	119.6616	115.5202273	118.3139568
C		41.65	144.8987021	142.3582	147.1206494	145.3447179
C		26.64	157.0578054	156.4273	157.3747557	157.447891
C		38	148.3431981	148.9797	149.0064034	149.3721678
C	x	173.3	7.169654909	7.1917	5.121748154	7.48418132
C	x	173.39	7.218771158	7.3158	6.440240586	7.236149573
C		36.84	145.8348635	146.0005	144.461187	146.1549197
H		3.677	28.02695731	28.00886667	28.011306	28.011306
H		1.86	30.45520092	30.27183333	29.76793936	29.67683857
H	x	6.573	23.99004664	24.376	25.19026426	24.9679349
H		3.632	28.05986338	27.9022	27.76969902	27.93289759
H		1.1	29.99640744	30.01366667	30.5900596	30.64285001
H		2.57	28.6419025	29.5373	29.65531996	29.16749268
H		2.536	29.26377249	28.5423	29.08403441	29.36698918
H		3.958	27.62906121	28.2017	28.57209643	27.91288773
H		1.34	30.3795499	30.27615	30.45909135	30.72380066
H		2.236	29.28149382	29.2387	29.46998874	29.34157809
H		2.553	29.00656041	29.1719	29.28269478	29.20757853
H		2.239	29.63126013	29.6944	29.01132568	29.43837273
H		2.239	29.5101827	29.4887	29.42337226	29.44690246
H		2.553	29.27543881	29.3767	29.14769161	29.2021562

Table S10. Accession numbers for halogenases.

Protein Name	Gene ID	Size (aa)	Putative Function	PFAM	Sequence Similarity (protein, origin, identity)
Cand1	ABNM25_RS00610	311	Non-haem Fe(II)/α-ketoglutarate (αKG)-dependent halogenase	pfam05721	WP_024692147.1 *Pseudomonas syringae* 100%
Cand2	ABNM25_RS23670	416	Flavin-dependent halogenase	pfam04820	RAQ67558.1 *Aspergillus flavus* 30.15%
Cand3	ABNM25_RS01535	338	Dechloroacutumine halogenase	pfam14226 pfam03171	QJD15032.1 *Sinomenium acutum* 25%
Cand4	ABNM25_RS24915	322	Dechloroacutumine halogenase	pfam14226 pfam03171	QJD15032.1 *Sinomenium acutum* 23.81%
Cand5 (SecA)	ABNM25_RS04855	338	TauD/TfdA family dioxygenase	pfam02668	WP_010418193.1 *Pseudomonas syringae* 100%

Table S11. Amoebicidal activity of syrilipamides and secimides.

Samples	IC50 (μg mL^{-1})
**1**	13.46 ± 3.79
**2**	5.48 ± 0.64
**3**	2.14 ± 0.63
**4**	49.17 ± 11.61
**5**	> 100
**1**+**2**+**3** (1:1:1)	1.10 ± 0.35
**4**+**5** (1:4)	27.08 ± 7.79
**1**+**2**+**3**+**4**+**5** (1:1:1:1:4)	5.51 ± 2.98

Note: all the mixtures are given as w/w in different ratios. n = 3 with ± SD.

Table S12. Antimicrobial activity assay of syrilipamides and secimides. Ciprofloxacin (antibiotic) and amphotericin B (antifungal) served as controls.

Organism	Solvent (methanol)	Ciprofloxacin (5 μg/mL in water)	Amphotericin B (10 μg/mL in DMSO/MeOH)	**1** (1 mg/mL in MeOH)	**2** (1 mg/mL in MeOH)	**3** (1 mg/mL in MeOH)	**4** (1 mg/mL in MeOH)	**5** (1 mg/mL in MeOH)
Bacillus subtilis 6633	0	29	-	0	12	13	0	0
Staphylococcus aureus 511	0	19	-	0	10	10	0	0
Pseudomonas aeruginosa SG 137	0	25	-	0	0	0	0	0
Enterococcus faecalis (VRSA) 1528	0	16	-	0	10	14	0	0
Mycobacterium vaccae 10670	0	23	-	0	17	18	0	0
Sporobolomyces salmonicolor 549	10	-	18	0	0	0	**33**	**25**
Candida albicans	0	-	20	0	0	0	0	0

Table S13. MIC value of secimides against *Sporobolomyces salmonicolor* 549 (μg mL^{-1}). Amphotericin B served as controls.

Organism	Solvent (methanol)	Amphotericin B (100 μg/mL in DMSO/MeOH)	**4** (100 μg/mL in MeOH)	**5** (100 μg/mL in MeOH)
Sporobolomyces salmonicolor 549	100	0.2	**25**	**25**

Table S14. Results of the broth dilution method assay for different human pathogenic fungi. The tested fungi included clinical isolates and strains obtained from the American Type Culture Collection (ATCC).

Species	Strain number	Voriconazol MIC in mg/L	**1** MIC in mg/L	**2** MIC in mg/L	**3** MIC in mg/L	**4** MIC in mg/L	**5** MIC in mg/L
Candida albicans	2016-303	≤0.016	>8	>8	>8	>8	>8
Candida auris	2019-731	8	>8	>8	>8	>8	>8
Candida glabrata	2018-606	0.06	>8	>8	>8	>8	>8
Candida guilliermondii	2020-194	0.125	>8	>8	>8	>8	>8
Candida krusei	2017-144	0.25	>8	>8	>8	>8	>8
Candida parapsilosis	ATCC22019	≤0.016	>8	>8	>8	>8	>8
Aspergillus fumigatus	2016-364	>8	>8	>8	>8	>8	>8
Aspergillus fumigatus	ATCC204305	0.25	>8	>8	>8	>8	>8
Fusarium petrolifilum	2020-183	>8	>8	>8	>8	>8	>8
Lomentospora prolificans	2020-066	>8	>8	>8	>8	>8	>8
Rhizopus arrhizus	2023-1175	4	>8	>8	>8	>8	>8
Scedosporium apiospermum	2020-135	0.125	>8	>8	>8	>8	>8

Table S15. List of primers used for amplification and cloning.

No.	Primer Name	Primer Sequence 5' to 3'
1	LA_sym_Fwd	GGAAGCATAAATGTAAAGCAATCACCTGGTACGCCGCG
2	LA_sym_Rev	TCGAGCAGGGTTGATGCGACGACGTCATGC
3	RA_sym_Fwd	GTCGCATCAACCCTGCTCGAATTGATGGG
4	RA_sym_Rev	GGAAATTAATTAAGGTACCGCCTCCAGCATGTAGCGCAAC
5	LA_secD_Fwd	GGAAGCATAAATGTAAAGCAAGCCTTTGCTTGGCTGCC
6	LA_secD_Rev	CCCAGGTCGCCGTTGGTGGCGTATGCCC
7	RA_secD_Fwd	GCCACCAACGGCGACCTGGGTGCTATCTAG
8	RA_secD_Rev	GGAAATTAATTAAGGTACCGCTGGGTTGTTCTCCCACG
9	LA_cand1_Fwd	GGAAGCATAAATGTAAAGCATGCCGGTGTGGCTCAGGG
10	LA_cand1_Rev	CTCAGGTGGGGAGCTTTTCCTTTTCGGTCAATGGGC
11	RA_cand1_Fwd	GGAAAAGCTCCCCACCTGAGTCAGACGAC
12	RA_cand1_Rev	GGAAATTAATTAAGGTACCGCGCCGCAGATGCCCATCA
13	LA_cand2_Fwd	GGAAGCATAAATGTAAAGCACTGGCCCAATTGCAGGCAC
14	LA_cand2_Rev	AACGGTCGATAGGTATTCCTTTTCGGTTGGGC
15	RA_cand2_Fwd	AGGAATACCTATCGACCGTTCCTGAGTG
16	RA_cand2_Rev	GGAAATTAATTAAGGTACCGGCTAAAAACAGTACGCGAATC
17	LA_cand3_Fwd	GGAAGCATAAATGTAAAGCAGGACATCATCCAGCCTATCC
18	LA_cand3_Rev	TGAGTTCATAACGGCTGTTTCCTCTCGC
19	RA_cand3_Fwd	AAACAGCCGTTATGAACTCATTGTCTTACGTCAACG
20	RA_cand3_Rev	GGAAATTAATTAAGGTACCGAGGCTCATCGATGCCAGG
21	LA_cand4_Fwd	GGAAGCATAAATGTAAAGCATGAAGGTCGCTACGTAAG
22	LA_cand4_Rev	GACCGAACGTTAACCGGTCCTGAAAGGG
23	RA_cand4_Fwd	GGACCGGTTAACGTTCGGTCAGGTTTTACCCTG
24	RA_cand4_Rev	GGAAATTAATTAAGGTACCGTTCGATGGCGCGCACACC
25	LA_secA_Fwd	GGAAGCATAAATGTAAAGCAATTTTGTCTCTCTGCTTAACGCTCAC
26	LA_secA_Rev	AACGAATAGAGACACCCCCCCAGAACT
27	RA_secA_Fwd	GGGGGGTGTCTCTATTCGTTGAGGGTGC
28	RA_secA_Rev	GGAAATTAATTAAGGTACCGTCGCTGAGGGGTTGAATAC
29	pET28a_cand1_Fwd	CGAAAACCTGTATTTTCAGGGATCCATGAGCAAAAAATTCGCTTTAAC
30	pET28a_cand1_Rev	GGTGCTCGAGTGCGGCCGCAAGCTTTCAGACCGCTTCGAATTTTTTG
31	pET28a_cand3_Fwd	CGAAAACCTGTATTTTCAGGGATCCATGACTCTGCACTATGTGCCC
32	pET28a_cand3_Rev	GGTGCTCGAGTGCGGCCGCAAGCTTTCAGGCCACCTTGCTGCC
33	pET28a_secA_Fwd	ACGACCGAAAACCTGTATTTTCAGGGATCCATGGGTTTAAAATTATCAAG
34	pET28a_secA_Rev	TGGTGGTGCTCGAGTGCGGCCGCAAGCTTTCAAGCCGACCCGGCCGACTC
35	KOC_sym_Fwd	CGGGACAAGCCCTCATTTCT
36	KOC_sym_Rev	GTCGATTCATGAGCGGCAAC
37	KOC_secD_Fwd	TATTGGCGGCTGTTGATGGT
38	KOC_secD_Rev	GGACGCCATCGAGCTGATTA
39	KOC_cand1_Fwd	CAGTGGCTTACCCGAGGTTT
40	KOC_cand1_Rev	ACTTCGTGCTCAAGGTCTGG
41	KOC_cand2_Fwd	GACTTTGTAGCTCAGCCGGT
42	KOC_cand2_Rev	TACTGAAGGGCAACACGCAT
43	KOC_cand3_Fwd	GGCGACGATCCAGACAAGAA
44	KOC_cand3_Rev	CCATCGAGCGTATTGCAAGC
45	KOC_cand4_Fwd	GGGTCGTCTGAATTGACGGT
46	KOC_cand4_Rev	GATCAAGGACCAGAACGGCA
47	KOC_secA_Fwd	TCCCAAGACCTGCAATCGAC
48	KOC_secA_Rev	CCAGGGCGATGTTCTTGACT
49	pET28a_check_Fwd	TAATACGACTCACTATAGG
50	pET28a_check_Rev	GCTAGTTATTGCTCAGCGG

Table S16. Plasmids used in this study.

plasmid	Primers for the construction	Primers for mutant verification	Reference
pEXG2			[13]
pET28a_H6TEV			[35]
pEXG2Δ*sym*	Left Arm [1]/[2] and Right Arm[3]/[4]	[35]/[36]	This study
pEXG2Δ*secD*	Left Arm [5]/[6] and Right Arm[7]/[8]	[37]/[38]	This study
pEXG2Δ*secD*Δ*sym*	Knock-out is based on the DSM1242Δ*secD* mutant	[37]/[38]	This study
pEXG2Δ*cand1*	Left Arm [9]/[10] and Right Arm[11]/[12]	[39]/[40]	This study
pEXG2Δ*cand2*	Left Arm [13]/[14] and Right Arm[15]/[16]	[41]/[42]	This study
pEXG2Δ*cand3*	Left Arm [17]/[18] and Right Arm[19]/[20]	[43]/[44]	This study
pEXG2Δ*cand4*	Left Arm [21]/[22] and Right Arm[23]/[24]	[45]/[46]	This study
pEXG2Δ*secA*	Left Arm [25]/[26] and Right Arm[27]/[28]	[47]/[48]	This study
pEXG2Δ*cand3*Δ*cand4*	Knock-out is based on the DSM1242Δ*cand3* mutant	[45]/[46]	This study
pEXG2Δ*cand3*Δ*cand4*Δ*cand1*	Knock-out is based on the DSM1242Δ*cand3*Δ*cand4* mutant	[39]/[40]	This study
pET28a-cand1	[29]/[30]	[49]/[50]	This study
pET28a-cand3	[31]/[32]	[49]/[50]	This study
pET28a-secA	[33]/[34]	[49]/[50]	This study

Table S17. Accession numbers for *sym* and *sec* coding genes in DSM 1242.

Gene ID	Size (aa)	Putative Function	Accession numbers	New function	BGC is responsible to
ABNM25_RS11975	251	helix-turn-helix domain-containing protein	WP_198712151.1		syrilipamides
ABNM25_RS11970	150	potassium channel family protein	WP_058444674.1		
ABNM25_RS11965	216	BON domain-containing protein	WP_346394666.1		
ABNM25_RS11960	190	GNAT family N-acetyltransferase	WP_198701207.1		
ABNM25_RS11955	416	ribonucleotide-diphosphate reductase subunit beta	WP_002554639.1		
ABNM25_RS11950	6672	non-ribosomal peptide synthase/polyketide synthase	WP_349341507.1	Core NRPS gene	
ABNM25_RS11945	199	cytochrome b	WP_024661715.1		
ABNM25_RS11940	199	TetR/AcrR family transcriptional regulator	WP_003434343.1		
ABNM25_RS11935	404	multidrug effflux MFS transporter	WP_349341506.1		
ABNM25_RS11930	79	hypothetical protein	WP_065088998.1		
ABNM25_RS11925	228	response regulator transcription factor	WP_349341505.1		
ABNM25_RS04870	329	LysR substrate-binding domain-containing protein	WP_346394924.1		secimides
ABNM25_RS04860	59	hypothetical protein	WP_162889022.1		
ABNM25_RS04855	338	TauD/TfdA family dioxygenase	WP_010418193.1	Halogenase	
ABNM25_RS04850	82	acyl carrier protein	WP_003399878.1		
ABNM25_RS04845	672	asparagine synthase (glutamine-hydrolyzing)	WP_346394925.1		

ABNM25_RS04840	3232	SDR family NAD(P)-dependent oxidoreductase	WP_346394926.1	
ABNM25_RS04835	2394	SDR family NAD(P)-dependent oxidoreductase	WP_346394927.1	Core PKS genes
ABNM25_RS04830	2261	SDR family NAD(P)-dependent oxidoreductase	WP_346394929.1	
ABNM25_RS04825	1056	ACP S-malonyltransferase	WP_198700444.1	
ABNM25_RS04820	306	hypothetical protein	WP_003392075.1	
ABNM25_RS04815	200	hypothetical protein	WP_346394930.1	
ABNM25_RS04810	519	bifunctional aminoglycoside phosphotransferase/ATP-binding protein	WP_074909835.1	
ABNM25_RS04800	293	class I SAM-dependent methyltransferase	WP_232913843.1	
ABNM25_RS04795	66	hypothetical protein	WP_116817992.1	
ABNM25_RS04790	86	hypothetical protein	WP_004416042.1	
ABNM25_RS04780	381	endonuclease/exonuclease/phosphatase family protein	WP_349341283.1	
ABNM25_RS04775	221	YciC family protein	WP_003368738.1	
ABNM25_RS04770	235	DUF2076 domain-containing protein	WP_016569021.1	
ABNM25_RS04765	159	NYN domain-containing protein	WP_003313837.1	
ABNM25_RS04760	204	3'-5' exonuclease	WP_003392086.1	
ABNM25_RS04755	156	Lrp/AsnC family transcriptional regulator	WP_004411882.1	
ABNM25_RS04750	305	drug/metabolite exporter YedA	WP_003313834.1	
ABNM25_RS04745	443	DEAD/DEAH box helicase	WP_003340788.1	
ABNM25_RS04740	413	TIGR03862 family flavoprotein	WP_183145851.1	
ABNM25_RS04735	306	histone deacetylase	WP_349341282.1	
ABNM25_RS04730	190	GNAT family N-acetyltransferase	WP_003313831.1	
ABNM25_RS04725	290	acyl-CoA thioesterase II	WP_346394933.1	
ABNM25_RS04720	197	HAD family hydrolase	WP_024693196.1	
ABNM25_RS04715	330	hypothetical protein	WP_003313828.1	
ABNM25_RS04710	298	neutral zinc metallopeptidase	WP_003313827.1	
ABNM25_RS04705	429	MFS transporter	WP_024691904.1	

Table S18. Energy analysis for conformers of **4a–4h** at B3LYP/6-31G(d) level in the gas phase

Name	Gibbs free energy (hatree)	ΔG (kcal/mol)	Population	Optimized conformers
**4a1**	-1588.991208	1.406876075	3.70%	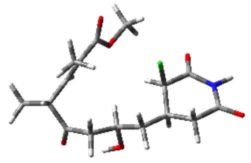
**4a2**	-1588.99345	0	39.89%	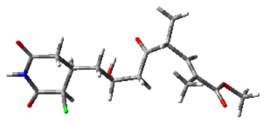
**4a3**	-1588.990871	1.618346743	2.59%	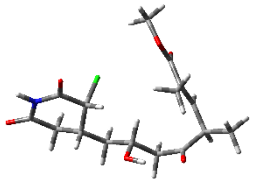
**4a4**	-1588.993215	0.147464709	31.09%	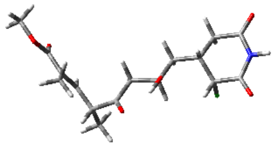

**4a5**	-1588.992857	0.372113074	21.27%	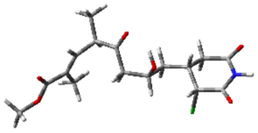
**4a6**	-1588.990324	1.961594384	1.45%	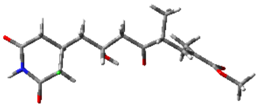
**4b1**	-1588.991054	1.22364333	8.82%	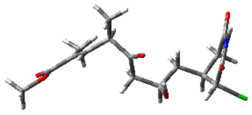
**4b2**	-1588.991427	0.989582324	13.10%	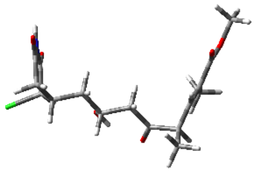
**4b3**	-1588.990374	1.650349722	4.29%	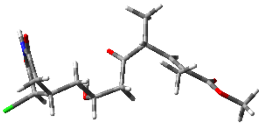
**4b4**	-1588.993004	0	69.76%	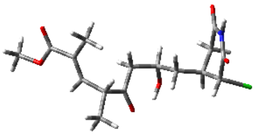
**4b5**	-1588.990312	1.689255305	4.02%	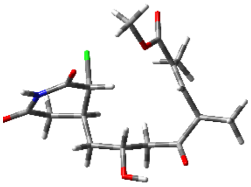
**4c1**	-1588.991837	1.482804712	4.07%	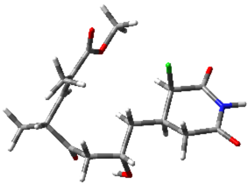
**4c2**	-1588.991366	1.77836164	2.47%	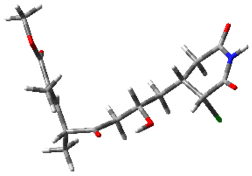

**4c3**	-1588.9942	0	49.84%	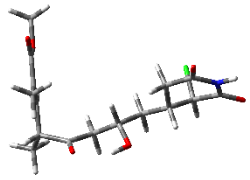
**4c4**	-1588.992396	1.132026958	7.36%	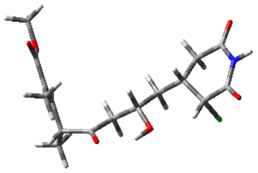
**4c5**	-1588.992484	1.07680613	8.08%	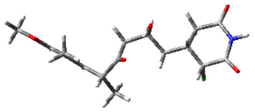
**4c6**	-1588.990975	2.023717815	1.63%	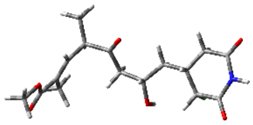
**4c7**	-1588.993445	0.473769597	22.38%	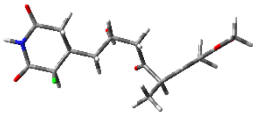
**4c8**	-1588.99186	1.468371996	4.17%	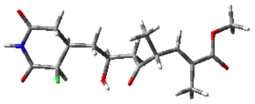
**4d1**	-1588.99521	0	45.52%	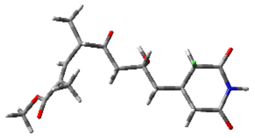
**4d2**	-1588.994621	0.369603037	24.38%	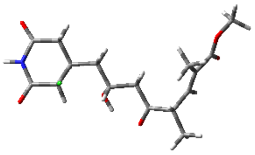
**4d3**	-1588.99403	0.740461092	13.03%	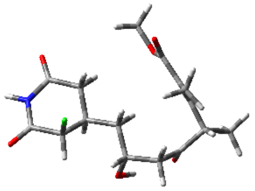

**4d4**	-1588.991924	2.061995888	1.40%	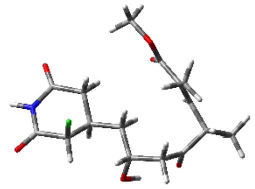
**4d5**	-1588.992963	1.410013622	4.20%	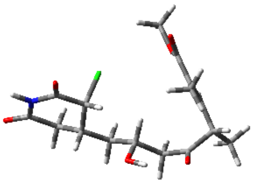
**4d6**	-1588.992008	2.009285099	1.53%	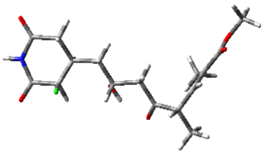
**4d7**	-1588.992449	1.732553453	2.44%	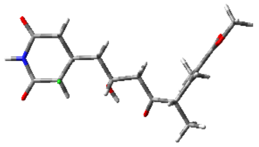
**4d8**	-1588.992108	1.946534159	1.70%	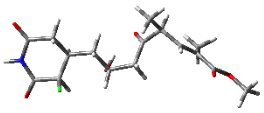
**4d9**	-1588.992256	1.853662768	1.99%	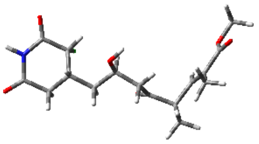
**4d10**	-1588.992875	1.465234449	3.83%	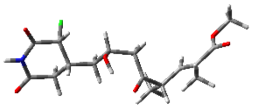
**4e1**	-1588.992887	0	33.10%	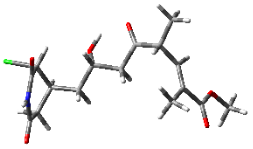
**4e2**	-1588.992377	0.320029794	19.27%	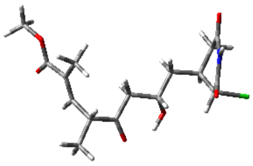

**4e3**	-1588.992227	0.414156204	16.44%	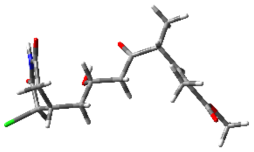
**4e4**	-1588.989708	1.994852383	1.14%	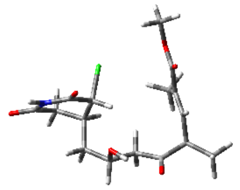
**4e5**	-1588.989677	2.014305174	1.10%	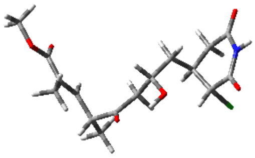
**4e6**	-1588.99135	0.964481948	6.49%	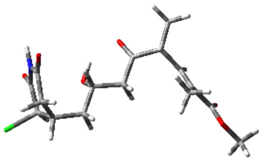
**4e7**	-1588.989754	1.96598695	1.19%	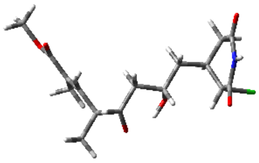
**4e8**	-1588.990434	1.539280558	2.46%	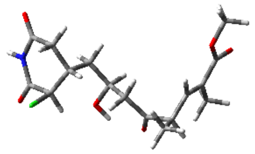
**4e9**	-1588.990363	1.583833726	2.28%	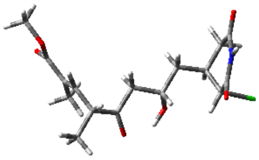
**4e10**	-1588.991961	0.581073705	12.40%	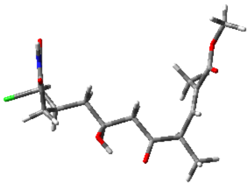
**4e11**	-1588.990201	1.685490248	1.92%	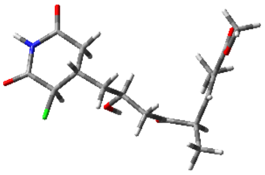

**4e12**	-1588.990342	1.597011423	2.23%	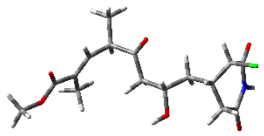
**4f1**	-1588.99544	0	55.08%	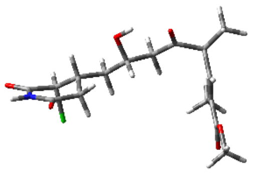
**4f2**	-1588.994654	0.493222388	23.94%	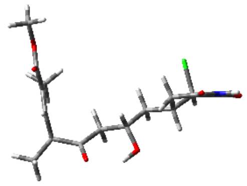
**4f3**	-1588.994332	0.695280415	17.01%	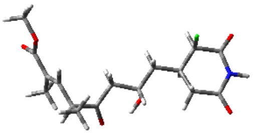
**4f4**	-1588.992329	1.952181743	2.03%	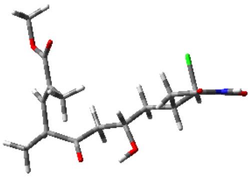
**4f5**	-1588.99228	1.982929704	1.93%	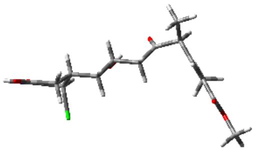
**4g1**	-1588.993418	0	56.69%	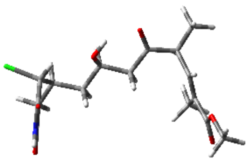
**4g2**	-1588.992753	0.417293751	28.01%	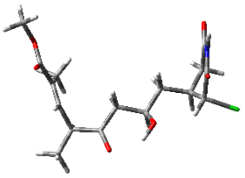
**4g3**	-1588.990436	1.871233031	2.40%	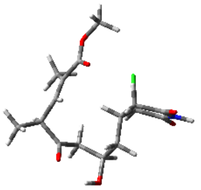

**4g4**	-1588.991929	0.934361497	11.69%	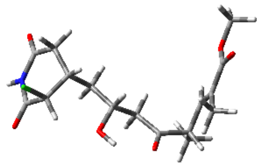
**4g5**	-1588.989795	2.273466556	1.22%	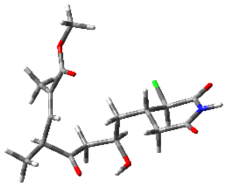
**4h1**	-1588.99195	0	30.10%	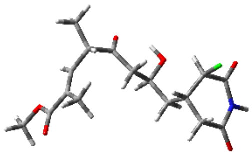
**4h2**	-1588.991497	0.284261758	18.62%	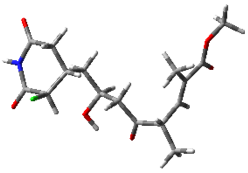
**4h3**	-1588.990278	1.049195717	5.11%	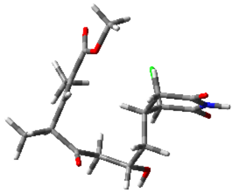
**4h4**	-1588.991946	0.002510038	29.97%	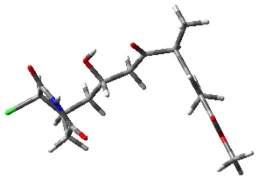
**4h5**	-1588.991366	0.36646549	16.20%	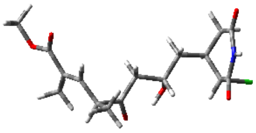

References

[1] S. Nurk, A. Bankevich, D. Antipov, A. A. Gurevich, A. Korobeynikov, A. Lapidus, A. D. Prjibelski, A. Pyshkin, A. Sirotkin, Y. Sirotkin, R. Stepanauskas, S. R. Clingenpeel, T. Woyke, J. S. McLean, R. Lasken, G. Tesler, M. A. Alekseyev, P. A. Pevzner,Assembling single-cell genomes and mini-metagenomes from chimeric MDA products *J. Comput. Biol.* **2013**, *20*, 714-737.

[2] T. Seemann,Prokka: rapid prokaryotic genome annotation *Bioinformatics* **2014**, *30*, 2068-2069.

[3] K. Blin, S. Shaw, H. E. Augustijn, Z. L. Reitz, F. Biermann, M. Alanjary, A. Fetter, B. R. Terlouw, W. W. Metcalf, E. J. N. Helfrich, G. P. van Wezel, M. H. Medema, T. Weber,antiSMASH 7.0: new and improved predictions for detection, regulation, chemical structures and visualisation *Nucleic Acids Res.* **2023**, *51*, W46-W50.

[4] T. O. Delmont, A. M. Eren,Linking pangenomes and metagenomes: the Prochlorococcus metapangenome *PeerJ* **2018**, *6*, e4320.

[5] A. M. Eren, E. Kiefl, A. Shaiber, I. Veseli, S. E. Miller, M. S. Schechter, I. Fink, J. N. Pan, M. Yousef, E. C. Fogarty,Community-led, integrated, reproducible multi-omics with anvi'o *Nat. Microbiol.* **2021**, *6*, 3-6.
[6] R. C. Edgar,MUSCLE: multiple sequence alignment with high accuracy and high throughput *Nucleic Acids Res.* **2004**, *32*, 1792-1797.
[7] C. L. M. Gilchrist, Y. H. Chooi,clinker & clustermap.js: automatic generation of gene cluster comparison figures *Bioinformatics* **2021**, *37*, 2473-2475.
[8] M. R. Olm, C. T. Brown, B. Brooks, J. F. Banfield,dRep: a tool for fast and accurate genomic comparisons that enables improved genome recovery from metagenomes through de-replication *The ISME journal* **2017**, *11*, 2864-2868.
[9] M. D. Lee,GToTree: a user-friendly workflow for phylogenomics *Bioinformatics* **2019**, *35*, 4162-4164.
[10] S. Capella-Gutierrez, J. M. Silla-Martinez, T. Gabaldon,trimAl: a tool for automated alignment trimming in large-scale phylogenetic analyses *Bioinformatics* **2009**, *25*, 1972-1973.
[11] M. N. Price, P. S. Dehal, A. P. Arkin,FastTree 2--approximately maximum-likelihood trees for large alignments *PLoS One* **2010**, *5*, e9490.
[12] I. Letunic, P. Bork,Interactive Tree Of Life (iTOL) v5: an online tool for phylogenetic tree display and annotation *Nucleic Acids Res.* **2021**, *49*, W293-W296.
[13] L. R. Hmelo, B. R. Borlee, H. Almblad, M. E. Love, T. E. Randall, B. S. Tseng, C. Lin, Y. Irie, K. M. Storek, J. J. Yang, R. J. Siehnel, P. L. Howell, P. K. Singh, T. Tolker-Nielsen, M. R. Parsek, H. P. Schweizer, J. J. Harrison,Precision-engineering the Pseudomonas aeruginosa genome with two-step allelic exchange *Nat. Protoc.* **2015**, *10*, 1820-1841.
[14] M. Arendrup, J. Meletiadis, J. Mouton, K. Lagrou, P. Hamal, J. Guinea, *EUCAST Definitive Document E.Def 7.3.2*; European Committee on Antimicrobial Susceptibility Testing: **2020**.
[15] M. P. Weinstein, *Methods for Dilution Antimicrobial Susceptibility Tests for Bacteria That Grow Aerobically*, 11th ed.; Clinical and Laboratory Standards Institute: Wayne, PA, **2018**.
[16] W. Li, A. Godzik,Cd-hit: a fast program for clustering and comparing large sets of protein or nucleotide sequences *Bioinformatics* **2006**, *22*, 1658-1659.
[17] L. Fu, B. Niu, Z. Zhu, S. Wu, W. Li,CD-HIT: accelerated for clustering the next-generation sequencing data *Bioinformatics* **2012**, *28*, 3150-3152.
[18] R. C. Edgar,Search and clustering orders of magnitude faster than BLAST *Bioinformatics* **2010**, *26*, 2460-2461.
[19] I. Letunic, P. Bork,Interactive Tree Of Life (iTOL) v4: recent updates and new developments *Nucleic Acids Res.* **2019**, *47*, W256-W259.
[20] Q. Zhu, M. Kosoy, K. Dittmar,HGTector: an automated method facilitating genome-wide discovery of putative horizontal gene transfers *BMC Genomics* **2014**, *15*, 717.
[21] B. Buchfink, C. Xie, D. H. Huson,Fast and sensitive protein alignment using DIAMOND *Nat. Methods* **2015**, *12*, 59-60.
[22] J. Arp, S. Gotze, R. Mukherji, D. J. Mattern, M. Garcia-Altares, M. Klapper, D. A. Brock, A. A. Brakhage, J. E. Strassmann, D. C. Queller, B. Bardl, K. Willing, G. Peschel, P. Stallforth,Synergistic activity of cosecreted natural products from amoebae-associated bacteria *Proc. Natl. Acad. Sci. U S A* **2018**, *115*, 3758-3763.
[23] J. Ni, Y. Sohma, M. Kanai,Scandium(iii) triflate-promoted serine/threonine-selective peptide bond cleavage *Chem. Commun. (Camb)* **2017**, *53*, 3311-3314.
[24] M. M. Zanardi, A. G. Suarez, A. M. Sarotti,Determination of the Relative Configuration of Terminal and Spiroepoxides by Computational Methods. Advantages of the Inclusion of Unscaled Data *J. Org. Chem.* **2017**, *82*, 1873-1879.
[25] N. Grimblat, M. M. Zanardi, A. M. Sarotti,Beyond DP4: an Improved Probability for the Stereochemical Assignment of Isomeric Compounds using Quantum Chemical Calculations of NMR Shifts *J. Org. Chem.* **2015**, *80*, 12526-12534.
[26] S. G. Smith, J. M. Goodman,Assigning stereochemistry to single diastereoisomers by GIAO NMR calculation: the DP4 probability *J. Am. Chem. Soc.* **2010**, *132*, 12946-12959.
[27] T. Yanai, D. P. Tew, N. C. Handy,A new hybrid exchange–correlation functional using the Coulomb-attenuating method (CAM-B3LYP) *Chem. Phys. Lett.* **2004**, *393*, 51-57.
[28] T. Bruhn, A. Schaumlöffel, Y. Hemberger, G. Pescitelli,SpecDis version 1.71 *Berlin, Germany* **2017**.
[29] G. M. Morris, R. Huey, W. Lindstrom, M. F. Sanner, R. K. Belew, D. S. Goodsell, A. J. Olson,AutoDock4 and AutoDockTools4: Automated docking with selective receptor flexibility *J. Comput. Chem.* **2009**, *30*, 2785-2791.
[30] O. Trott, A. J. Olson,AutoDock Vina: improving the speed and accuracy of docking with a new scoring function, efficient optimization, and multithreading *J. Comput. Chem.* **2010**, *31*, 455-461.

[31] J. Jumper, R. Evans, A. Pritzel, T. Green, M. Figurnov, O. Ronneberger, K. Tunyasuvunakool, R. Bates, A. Zidek, A. Potapenko, A. Bridgland, C. Meyer, S. A. A. Kohl, A. J. Ballard, A. Cowie, B. Romera-Paredes, S. Nikolov, R. Jain, J. Adler, T. Back, S. Petersen, D. Reiman, E. Clancy, M. Zielinski, M. Steinegger, M. Pacholska, T. Berghammer, S. Bodenstein, D. Silver, O. Vinyals, A. W. Senior, K. Kavukcuoglu, P. Kohli, D. Hassabis,Highly accurate protein structure prediction with AlphaFold *Nature* **2021**, *596*, 583-589.

[32] M. Frisch, G. Trucks, H. Schlegel, G. Scuseria, M. Robb, J. Cheeseman, G. Scalmani, V. Barone, G. Petersson, H. Nakatsuji,Gaussian 16, revision C. 02; Gaussian, Inc *Wallingford, CT* **2019**.

[33] S. Grimme, J. Antony, S. Ehrlich, H. Krieg,A consistent and accurate ab initio parametrization of density functional dispersion correction (DFT-D) for the 94 elements H-Pu *J. Chem. Phys.* **2010**, *132*.

[34] C. Notredame, D. G. Higgins, J. Heringa,T-Coffee: A novel method for fast and accurate multiple sequence alignment *J. Mol. Biol.* **2000**, *302*, 205-217.

[35] E. M. Huber, D. H. Scharf, P. Hortschansky, M. Groll, A. A. Brakhage,DNA minor groove sensing and widening by the CCAAT-binding complex *Structure* **2012**, *20*, 1757-1768.

NMR and HRMS spectra

^{1}H NMR spectrum in DMSO-d_6 for compound **1**

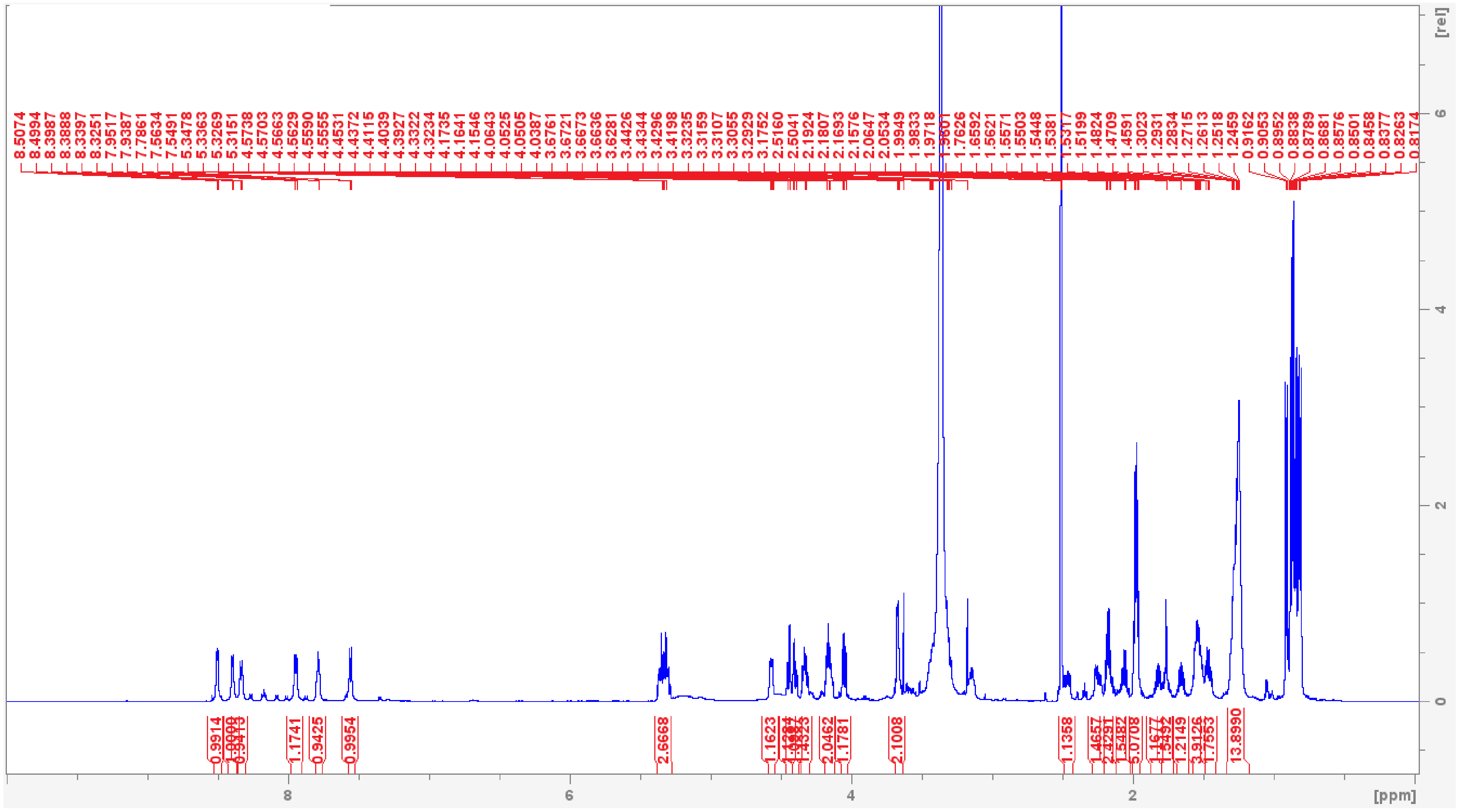

^{13}C NMR spectrum in DMSO-d_6 for compound **1**

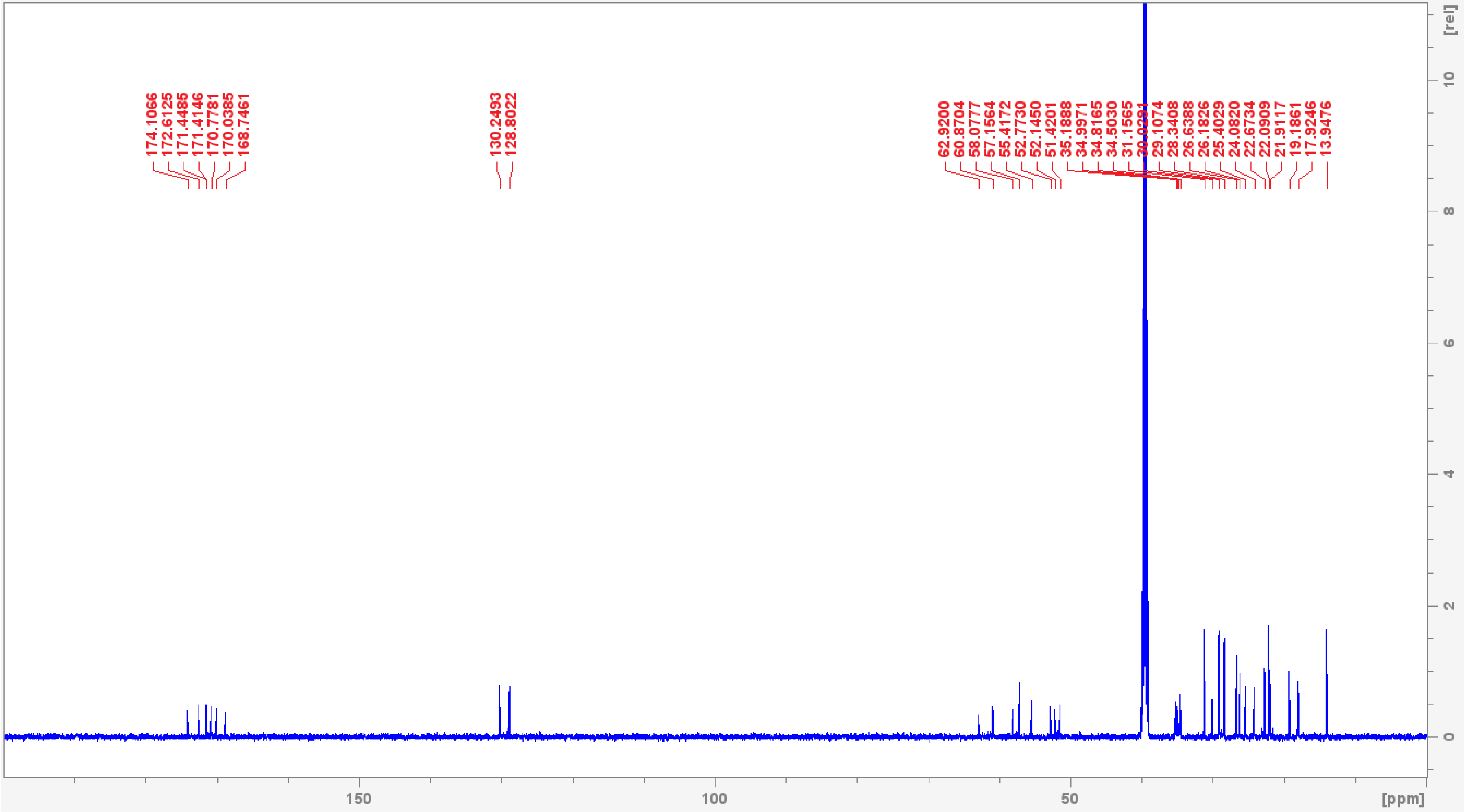

^{1}H-^{1}H COSY spectrum in DMSO-d_6 for compound **1**

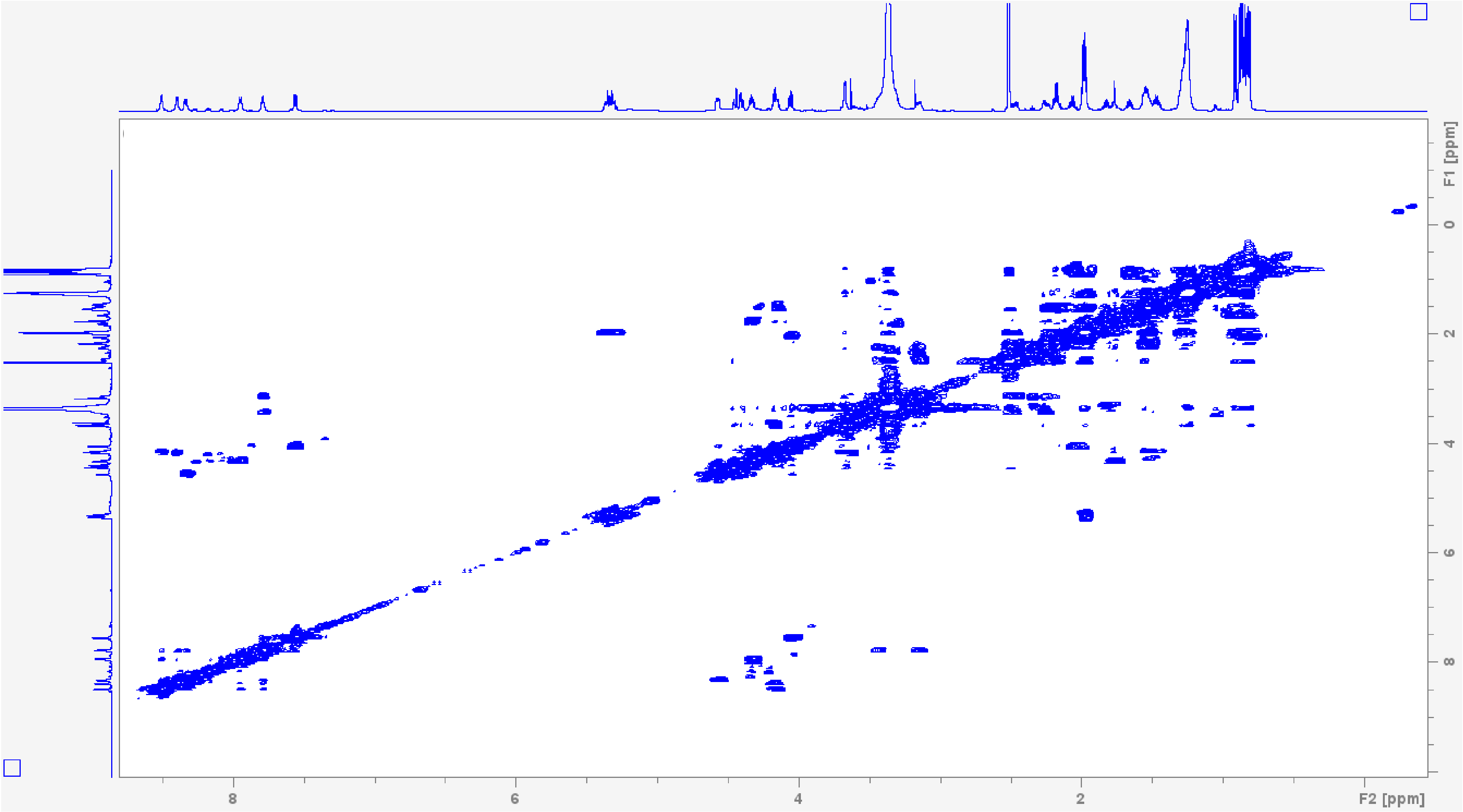

HSQC spectrum in DMSO-d_6 for compound **1**

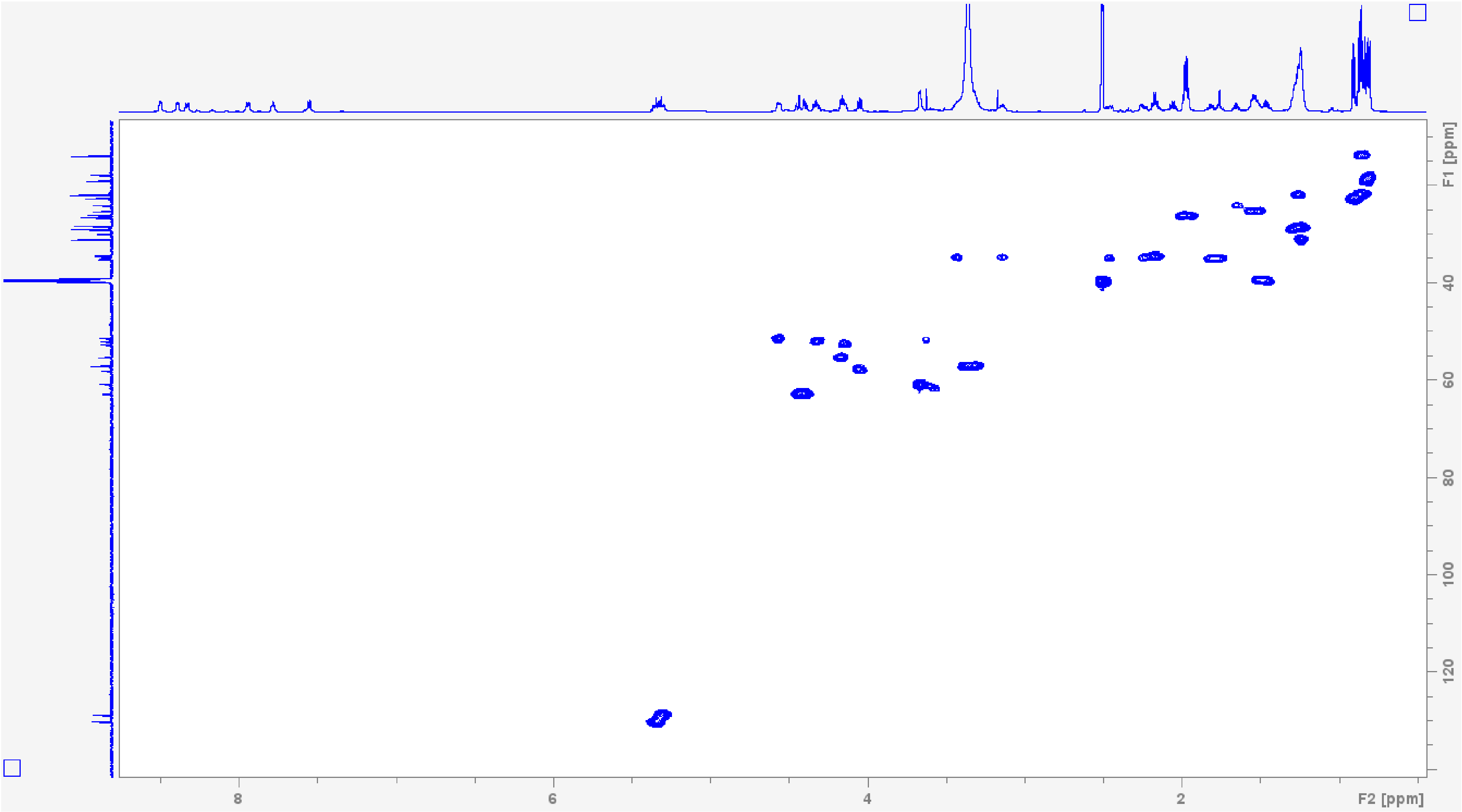

HMBC spectrum in DMSO-d_6 for compound **1**

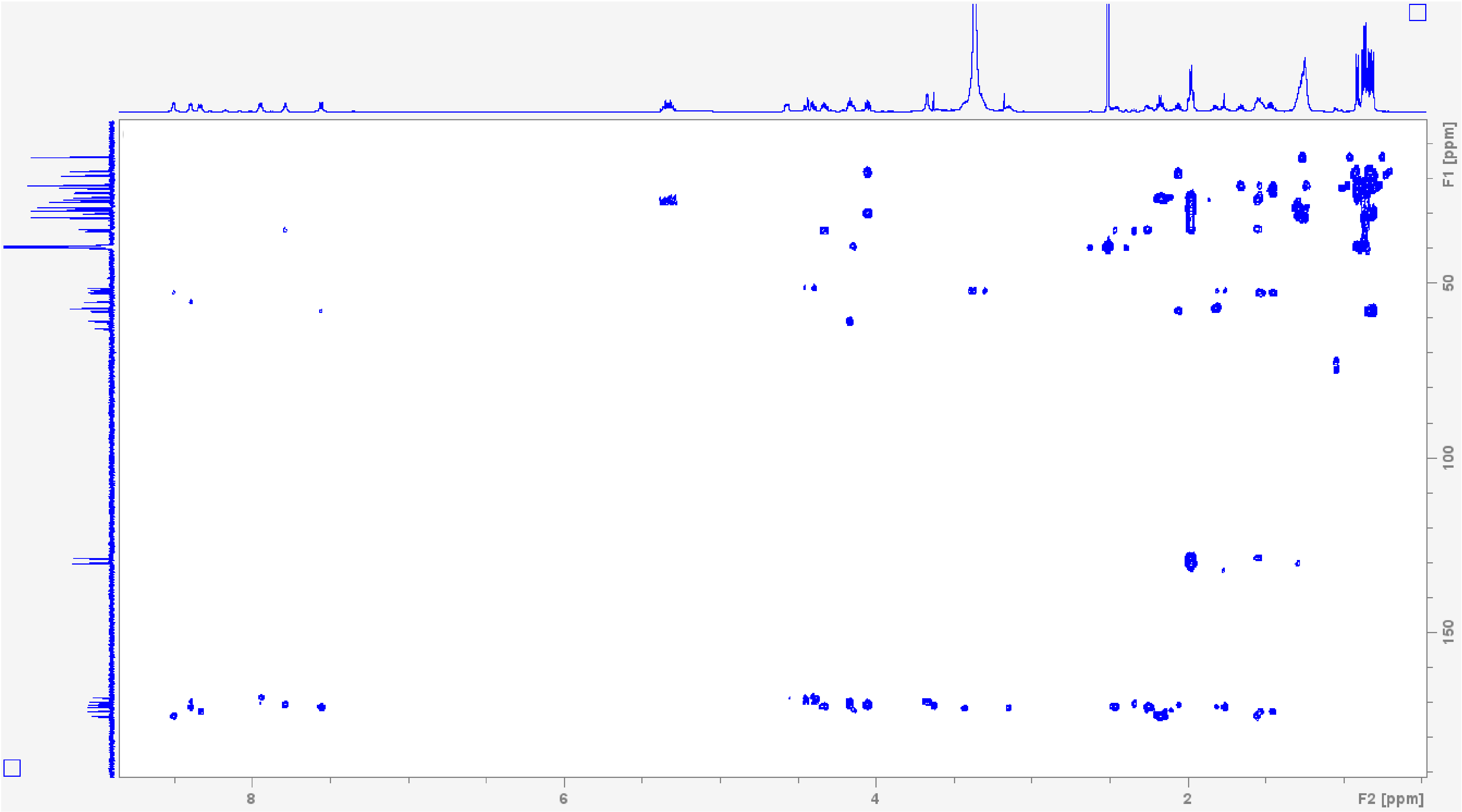

NOESY spectrum in DMSO-d_6 for compound **1**

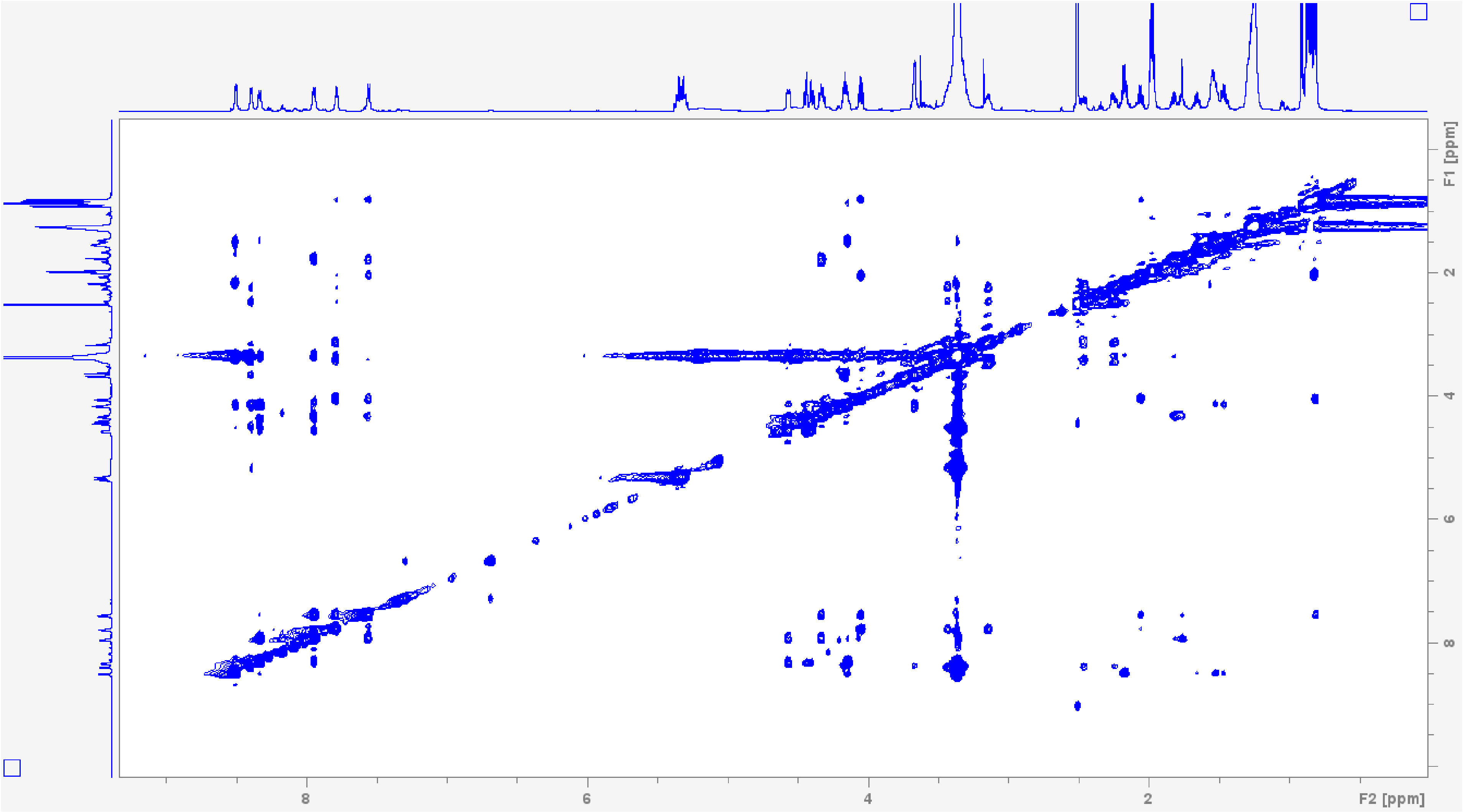

^{1}H NMR spectrum in chloroform-*d* for compound **1**

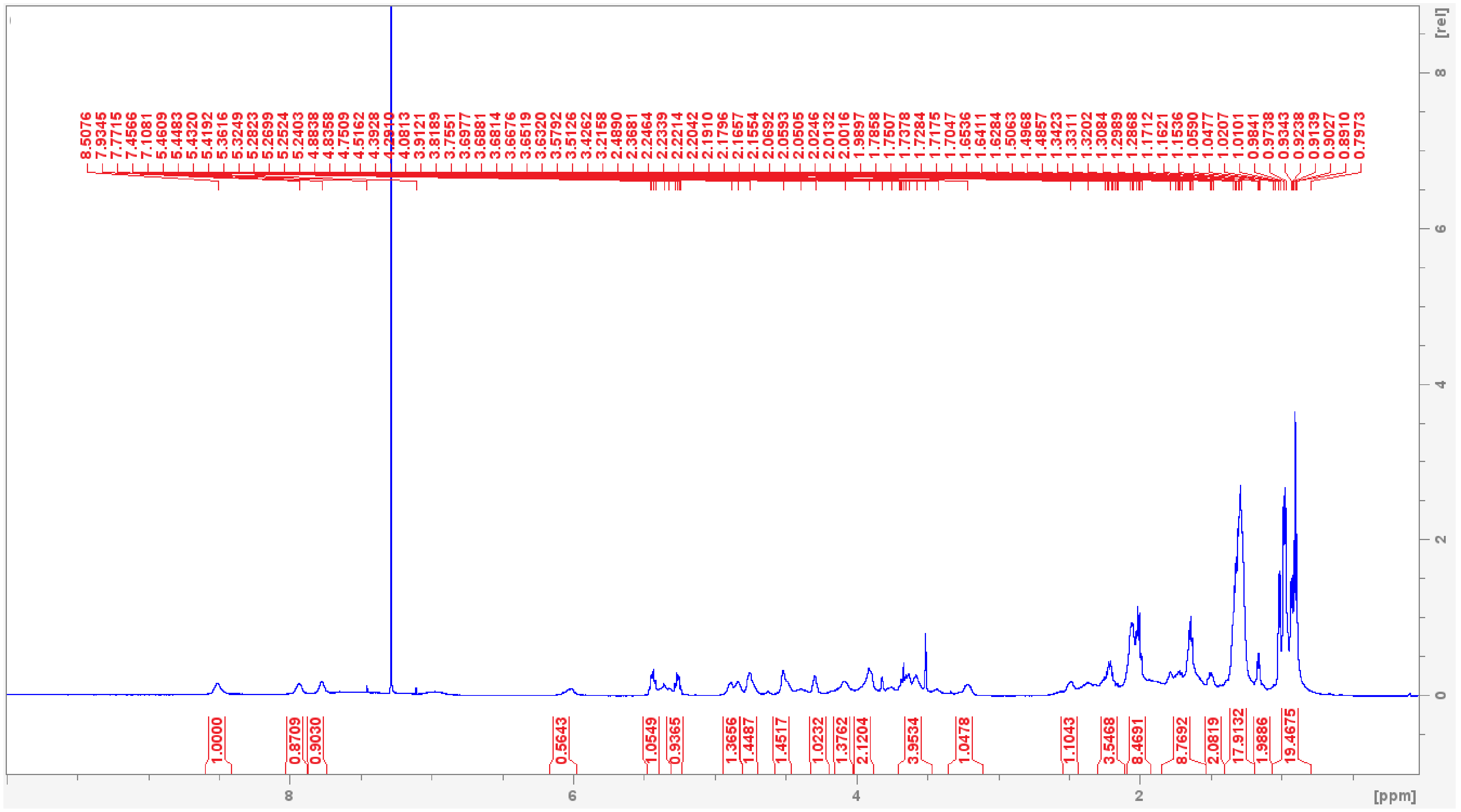

HRMS spectrum for compound **1**

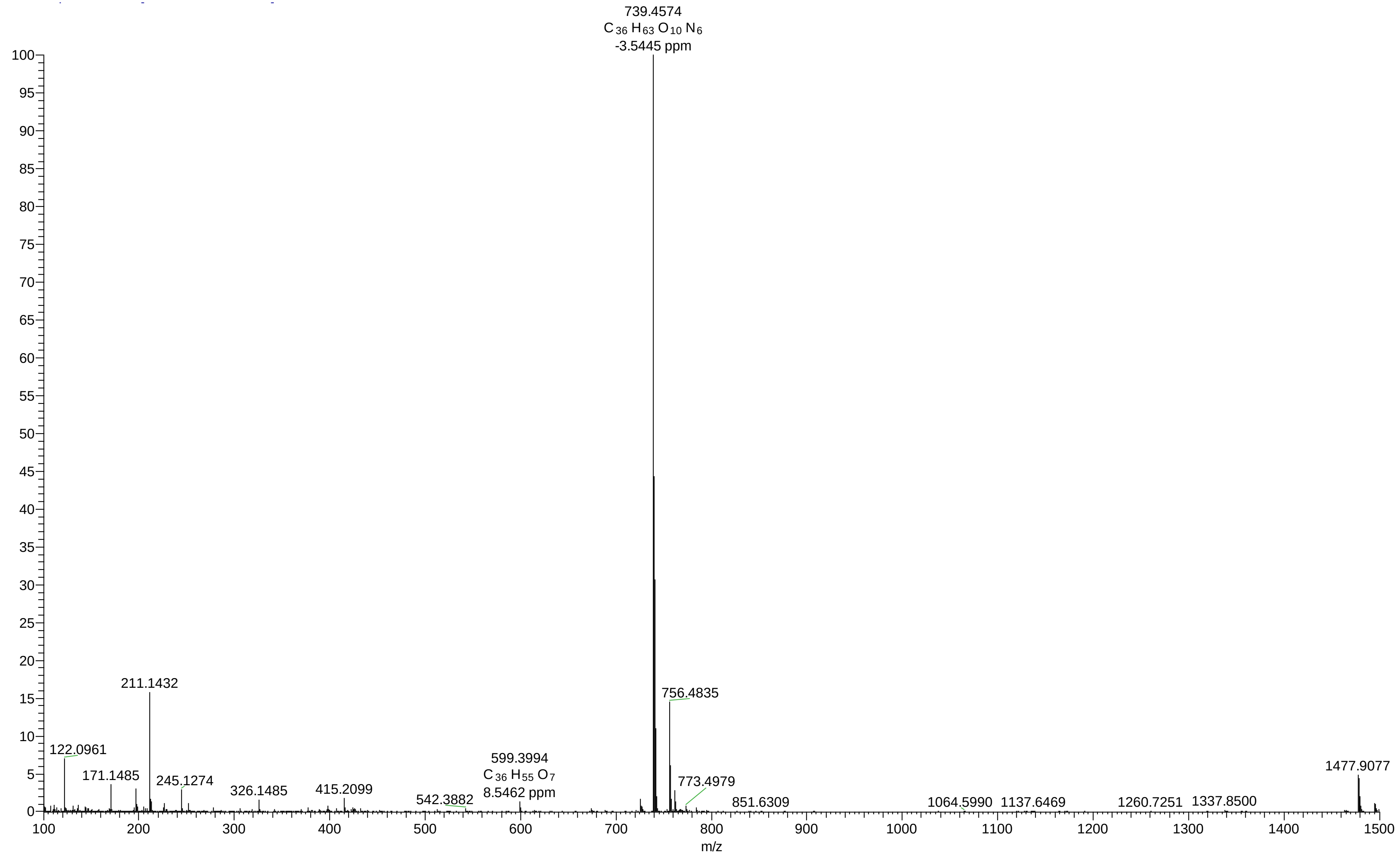

^{1}H NMR spectrum in DMSO-d_6 for compound **2**

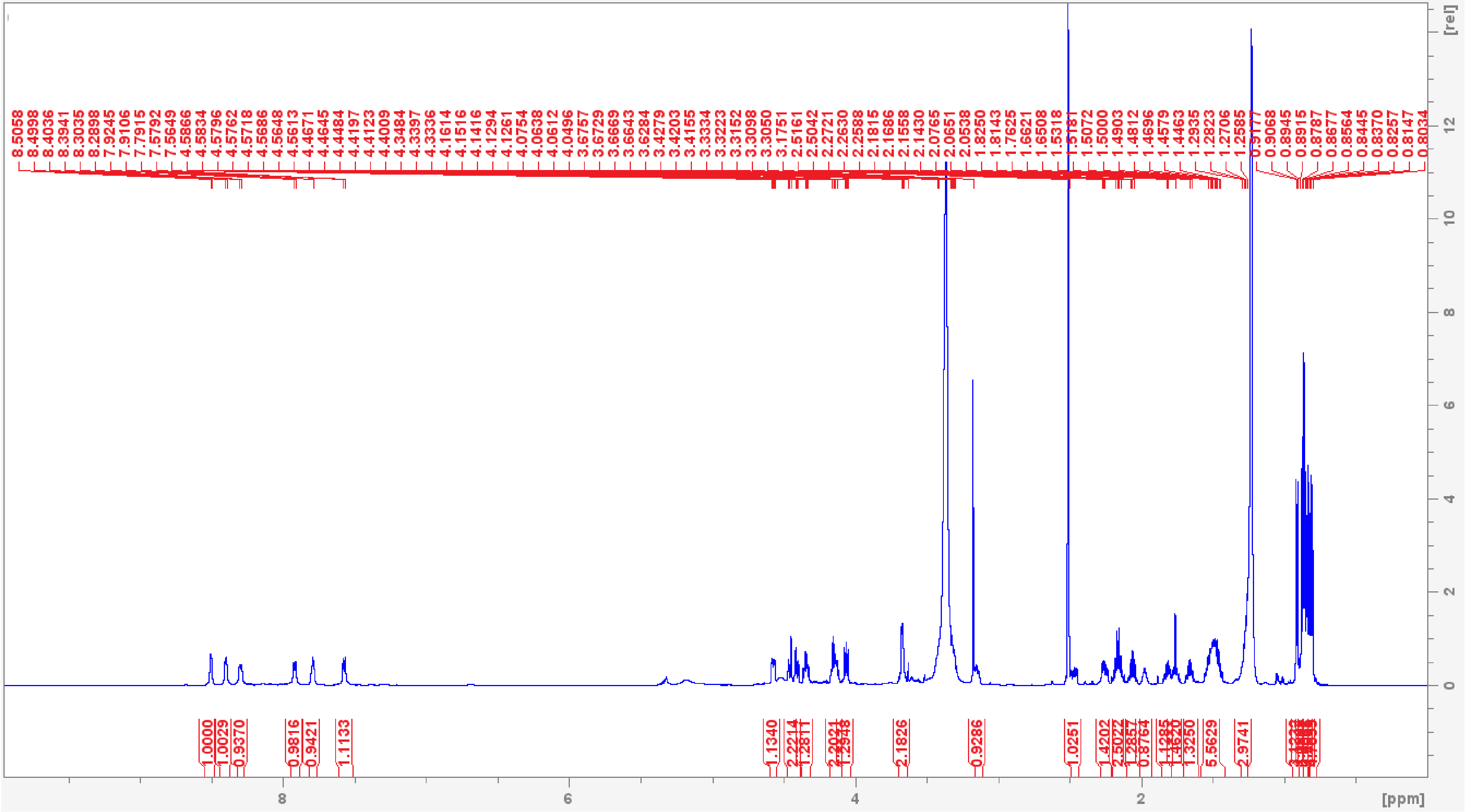

^{13}C NMR spectrum in DMSO-d_6 for compound **2**

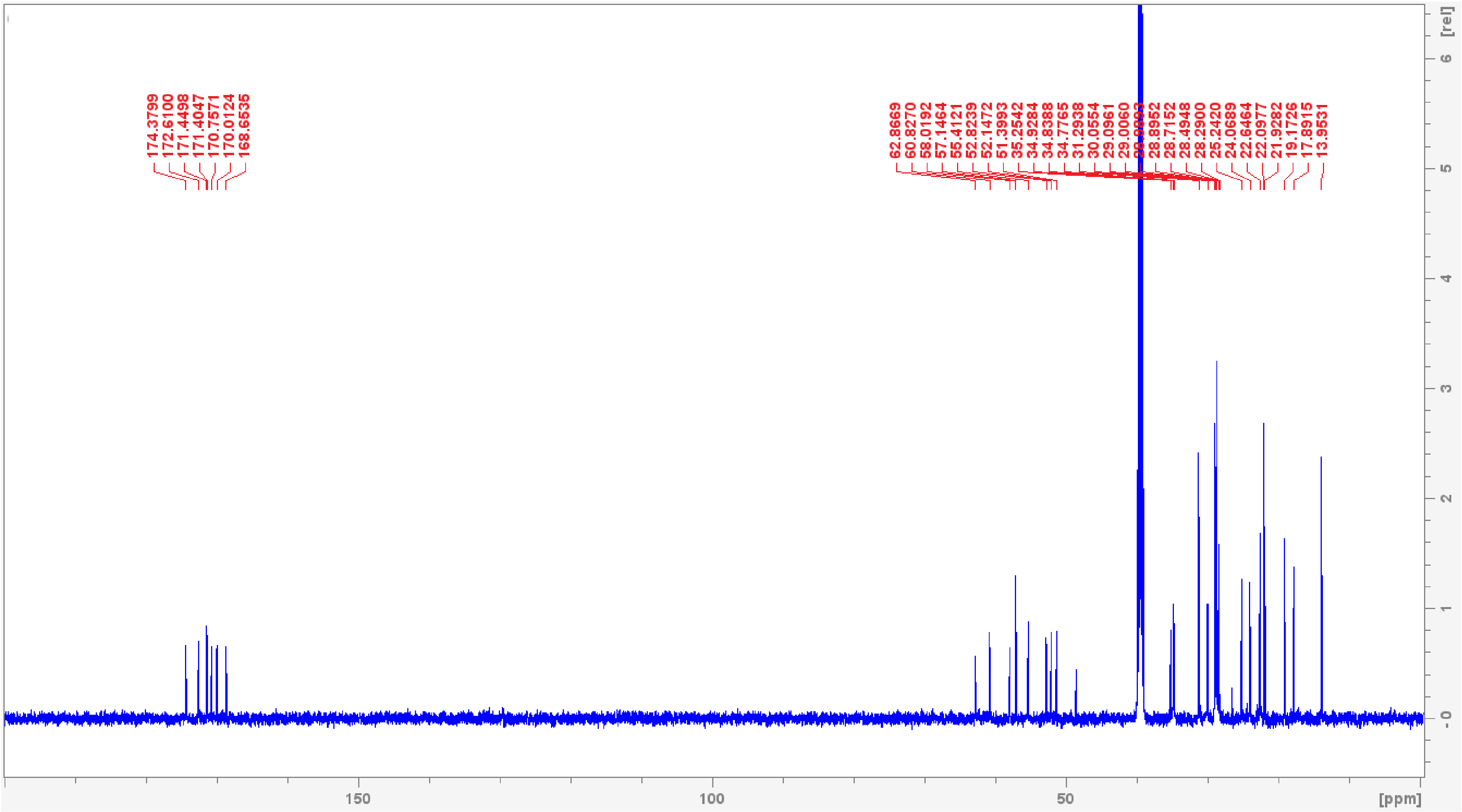

^{1}H-^{1}H COSY spectrum in DMSO-d_6 for compound **2**

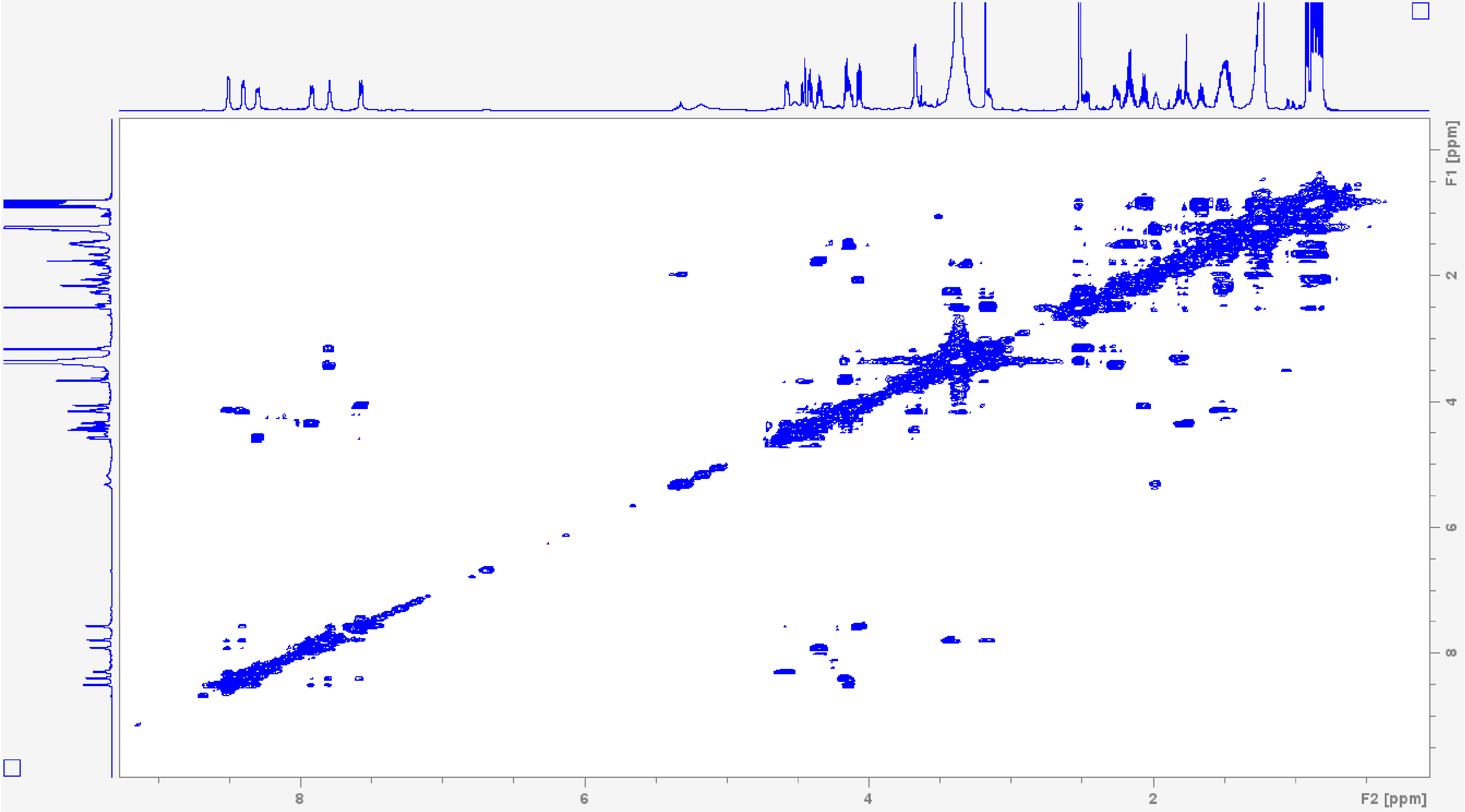

HSQC spectrum in DMSO-d_6 for compound **2**

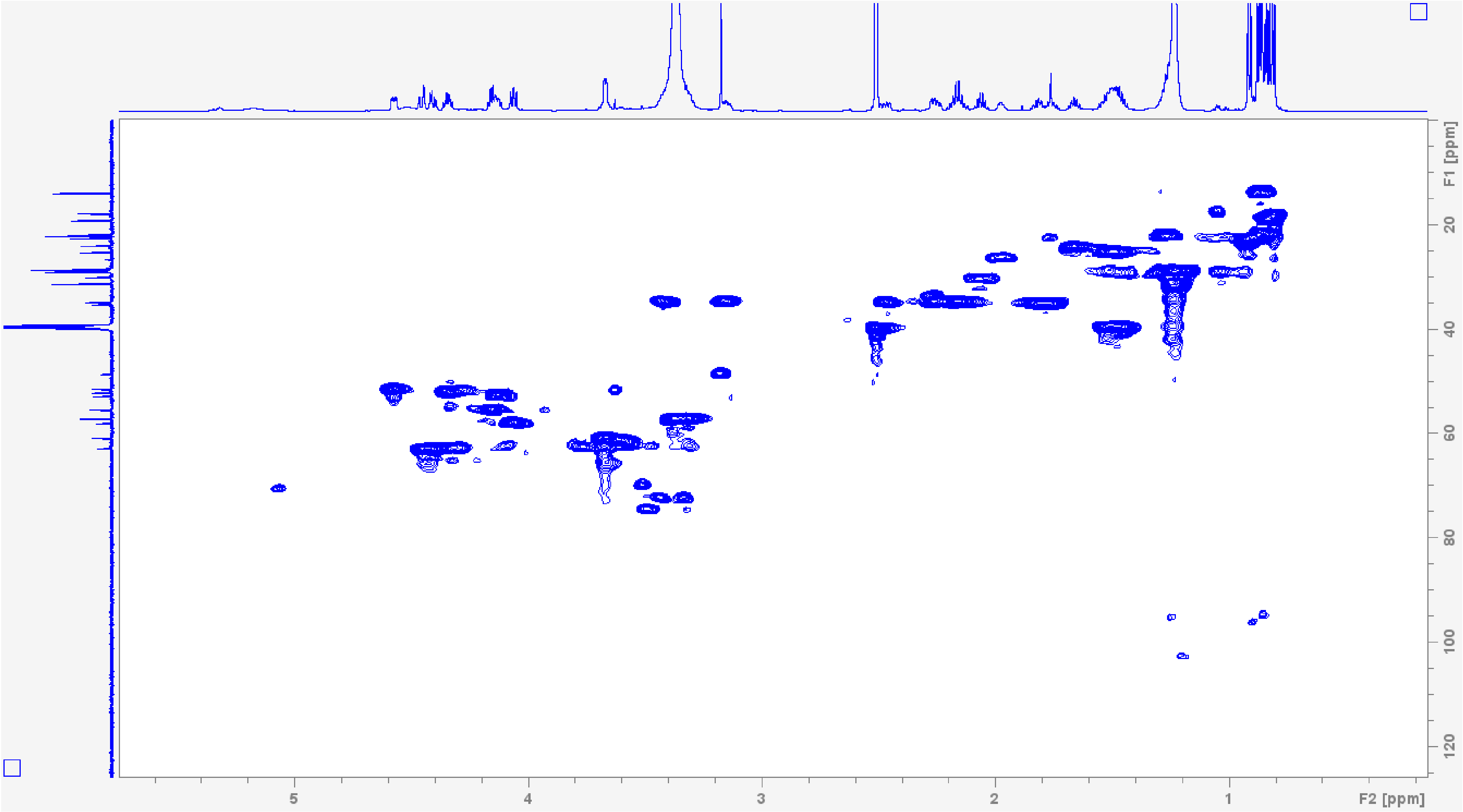

HMBC spectrum in DMSO-d_6 for compound **2**

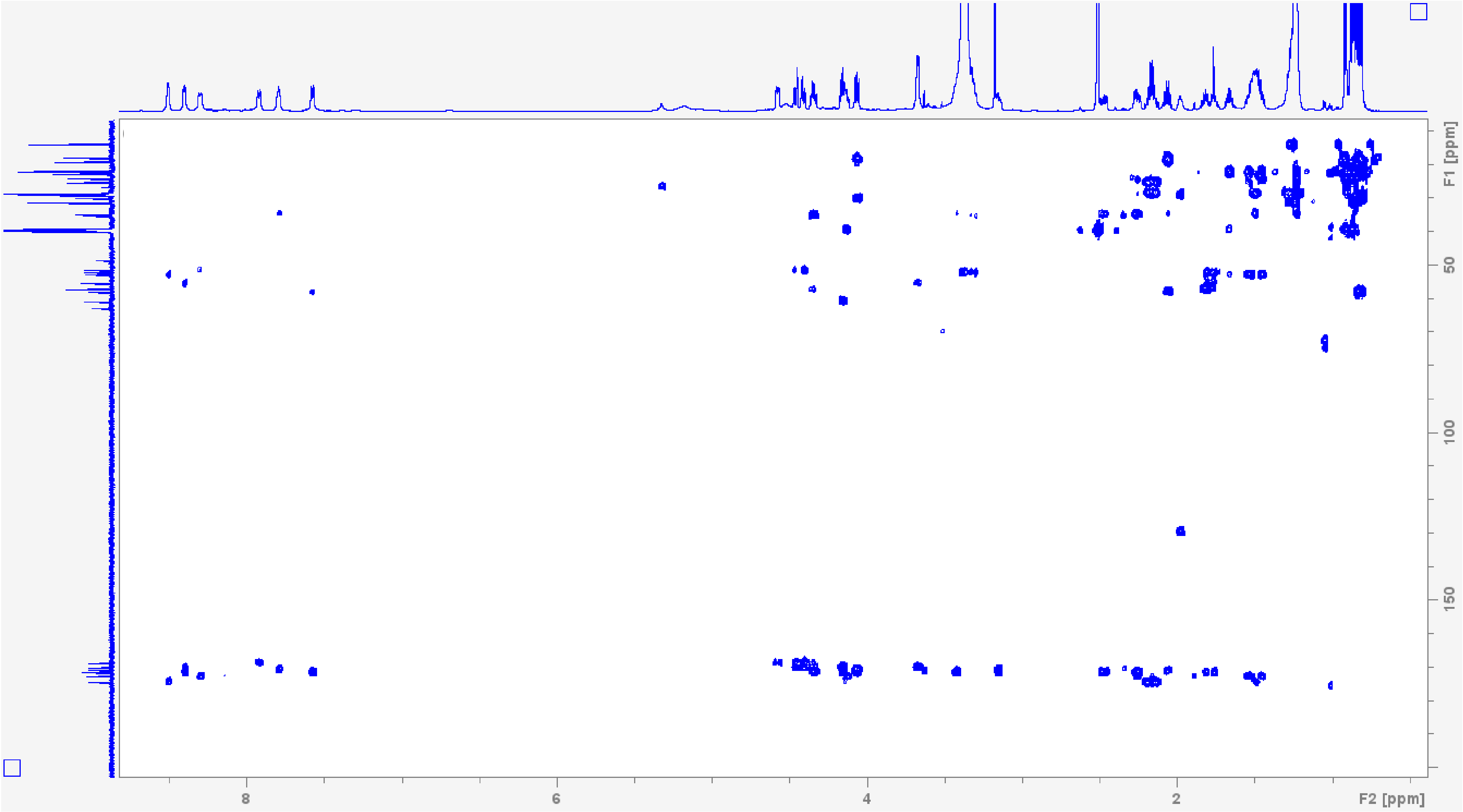

NOESY spectrum in DMSO-d_6 for compound **2**

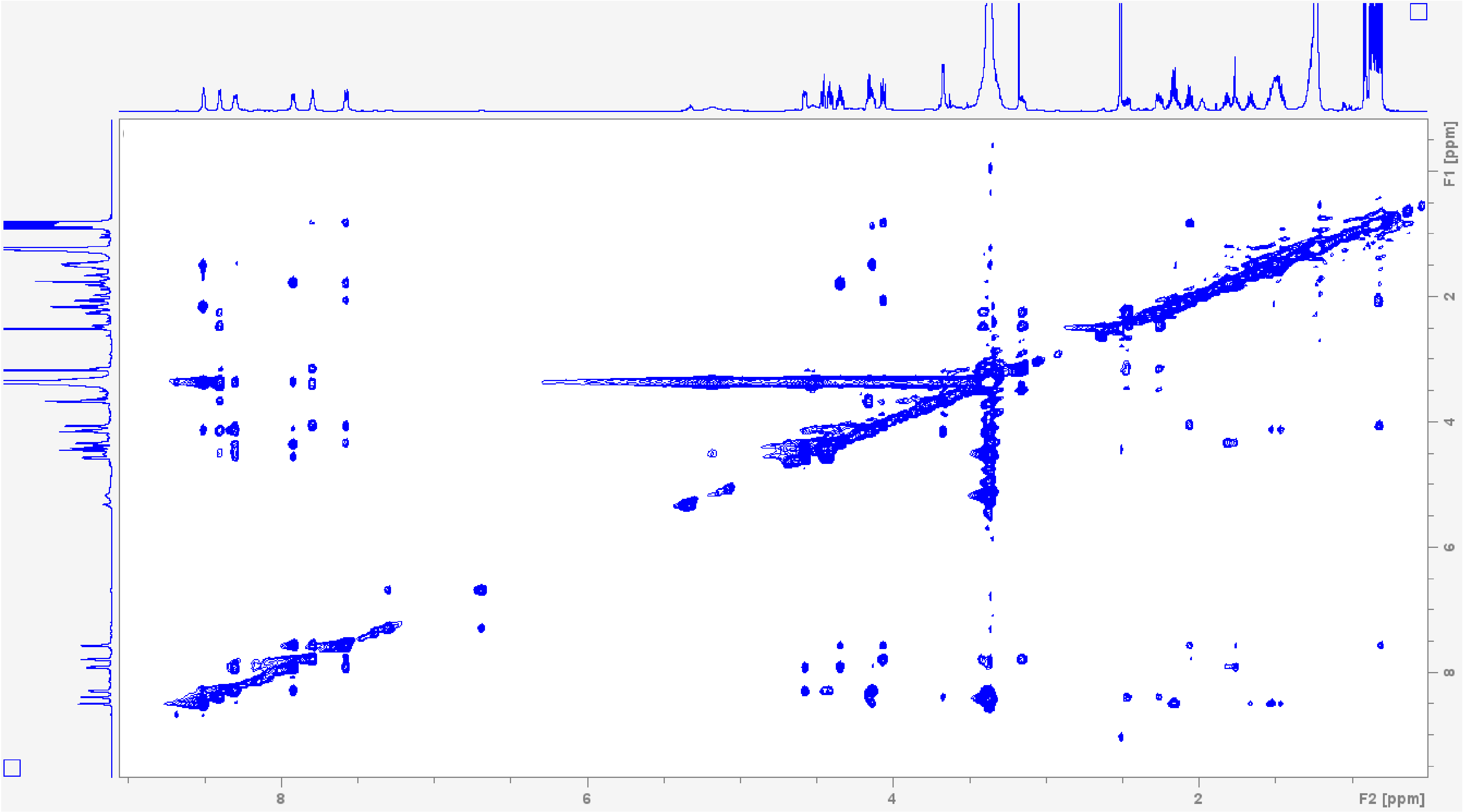

HRMS spectrum for compound **2**

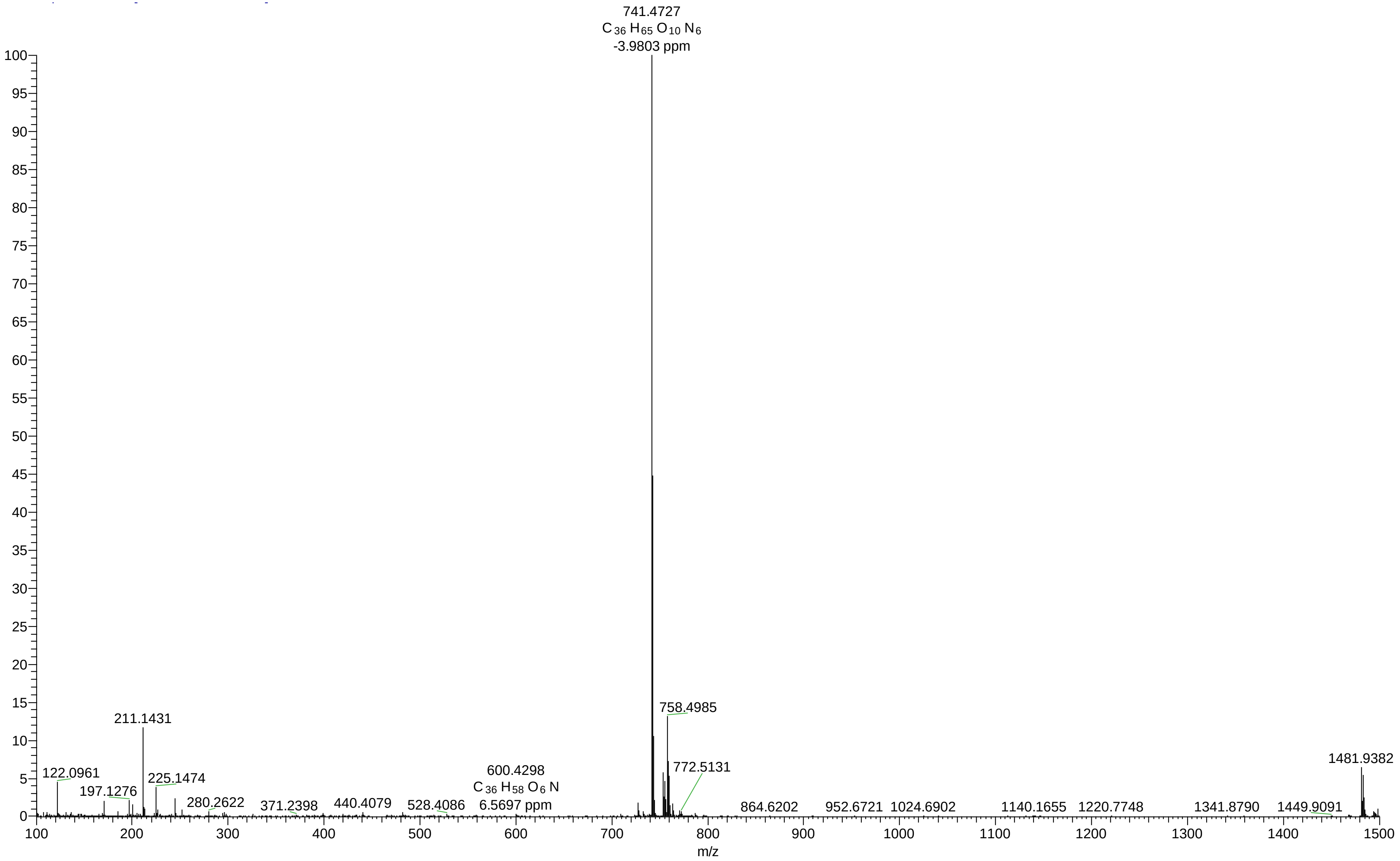

^{1}H NMR spectrum in DMSO-d_6 for compound **3**

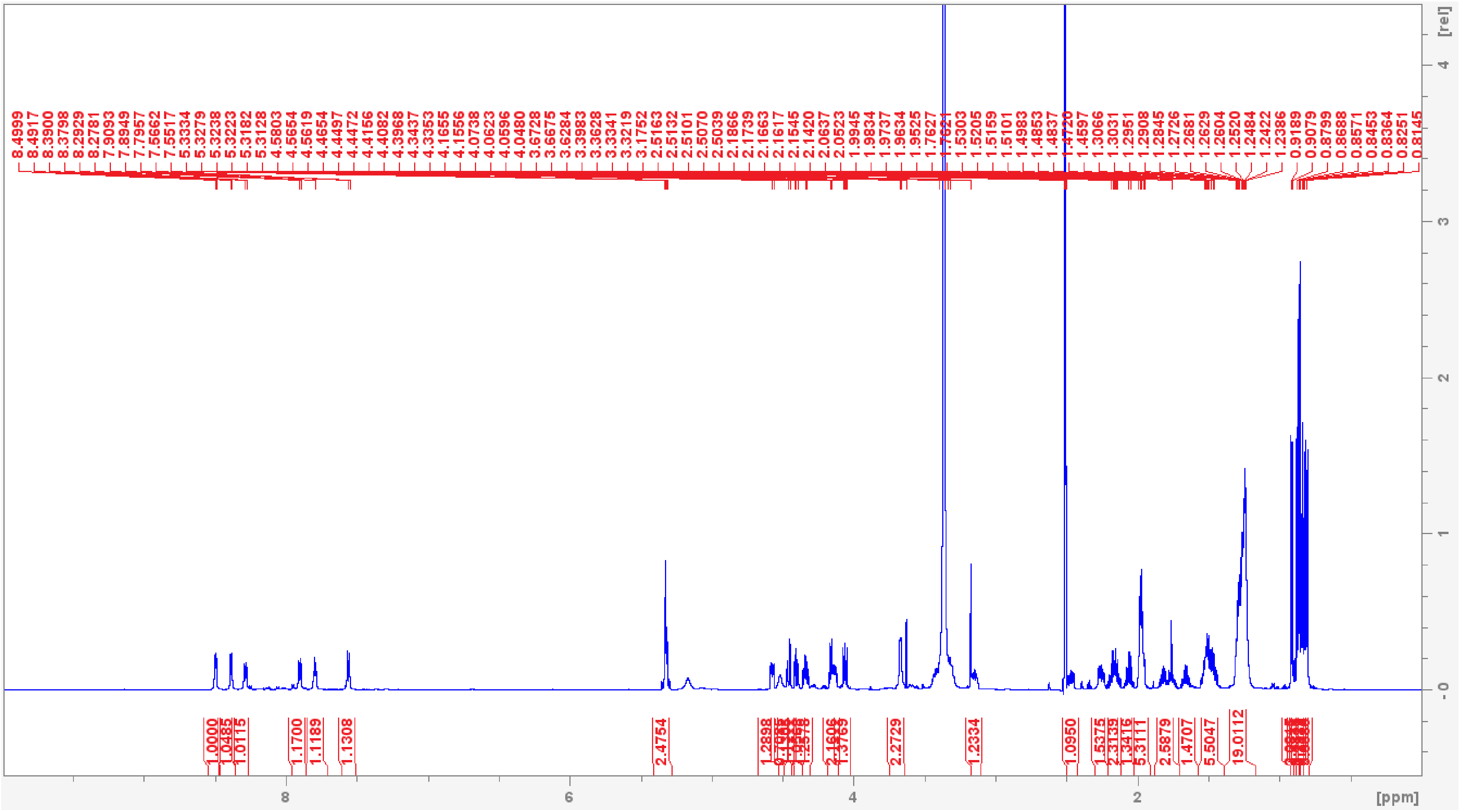

^{13}C NMR spectrum in DMSO-d_6 for compound **3**

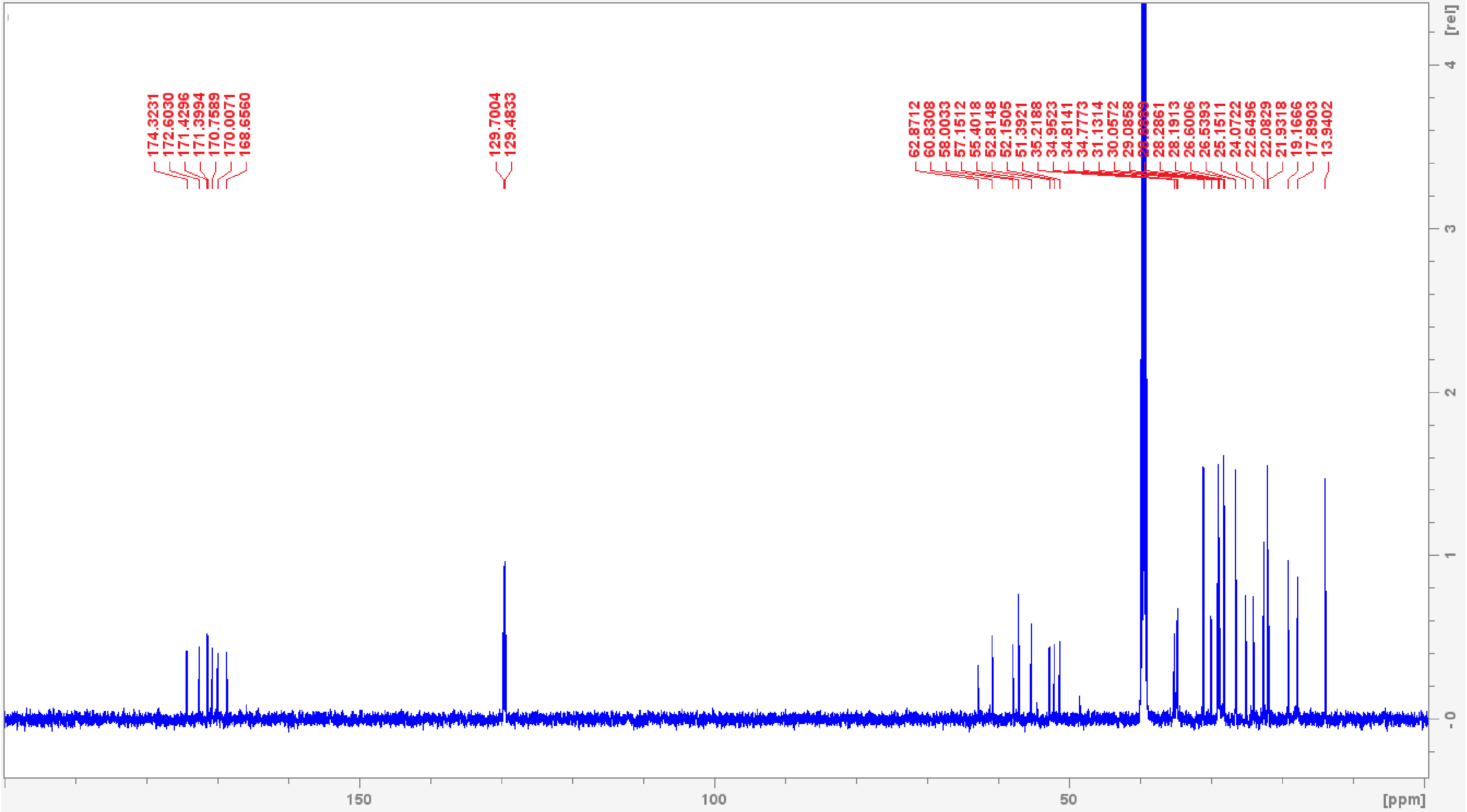

^{1}H-^{1}H COSY spectrum in DMSO-d_6 for compound **3**

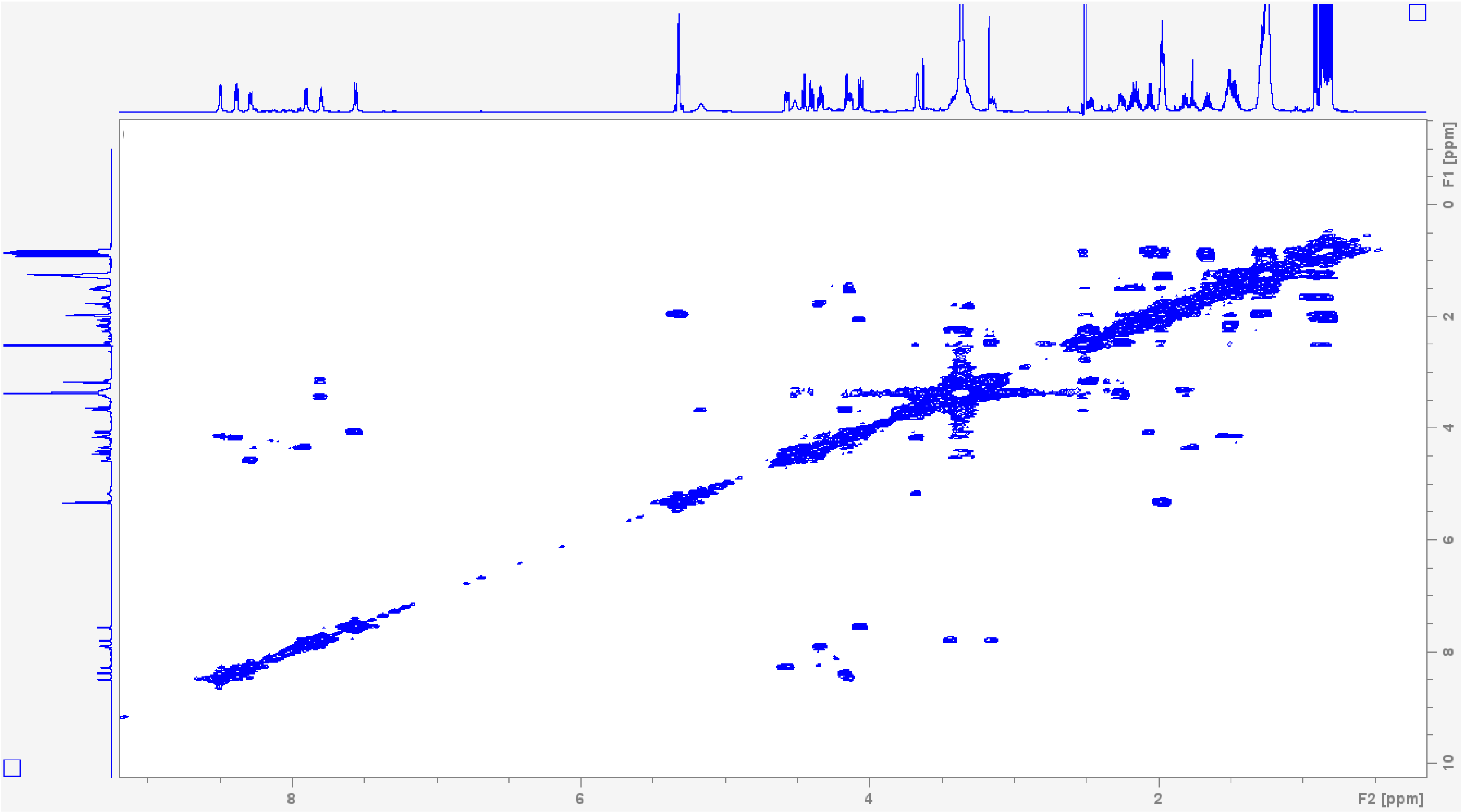

HSQC spectrum in DMSO-d_6 for compound **3**

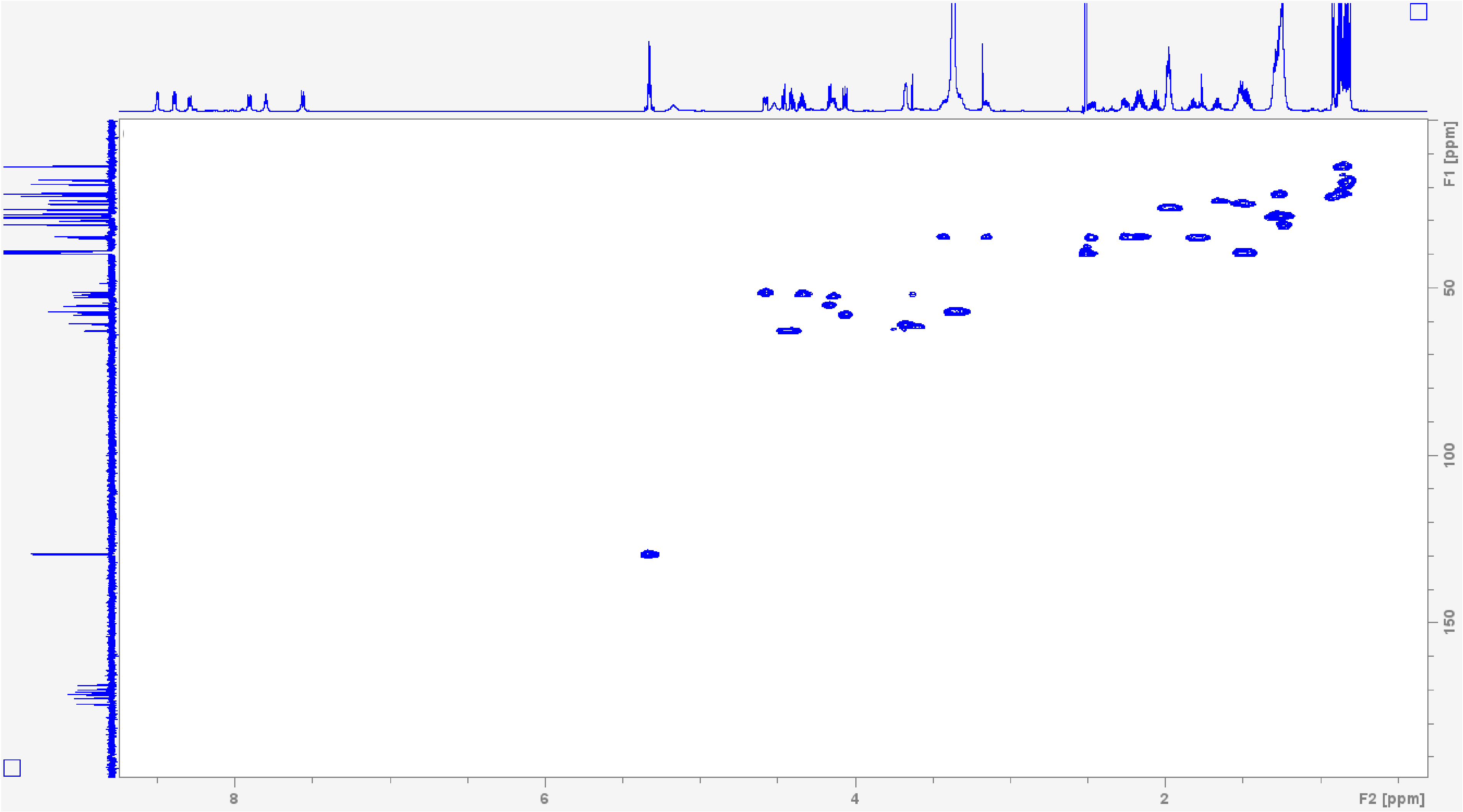

HMBC spectrum in DMSO-d_6 for compound **3**

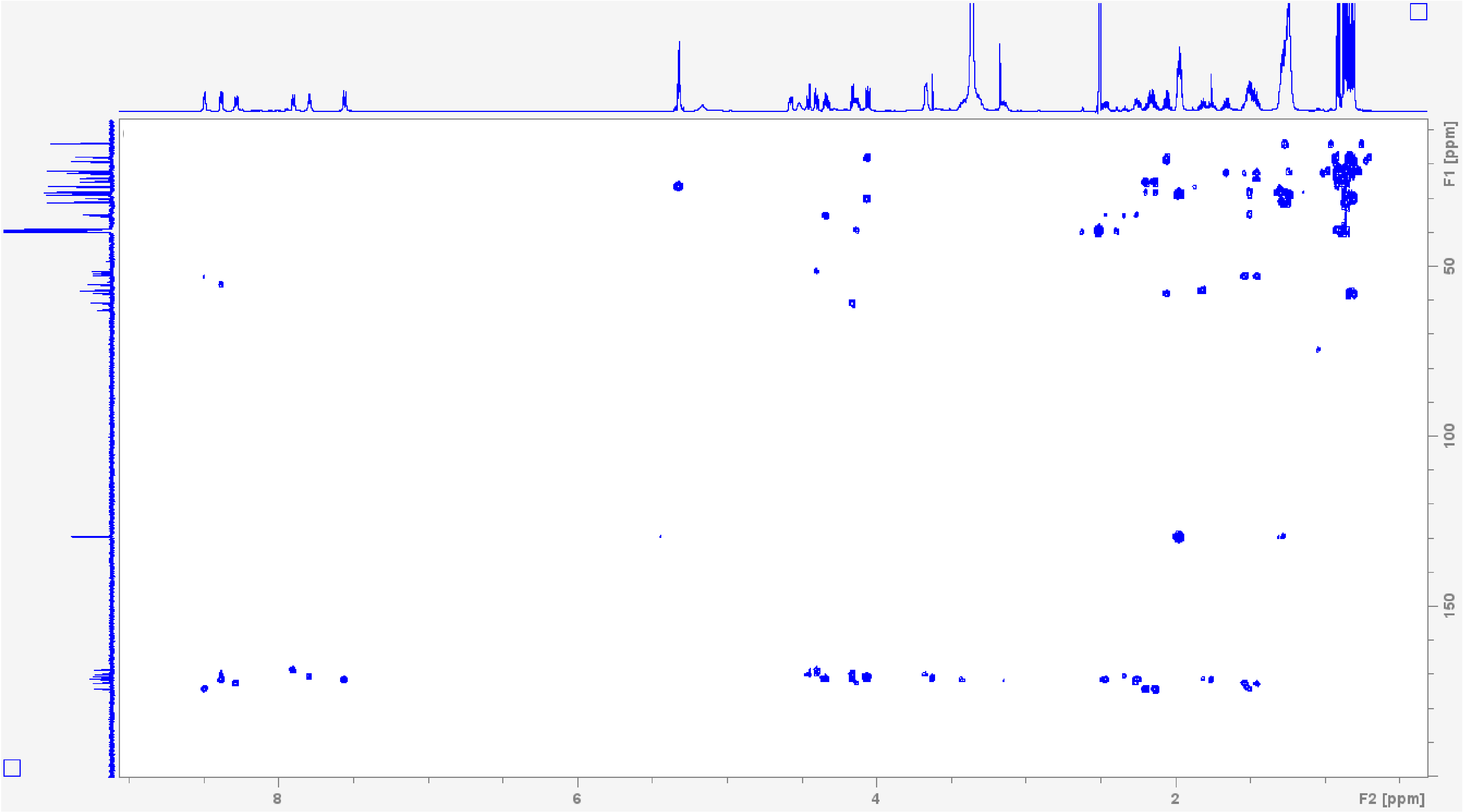

NOESY spectrum in DMSO-d_6 for compound **3**

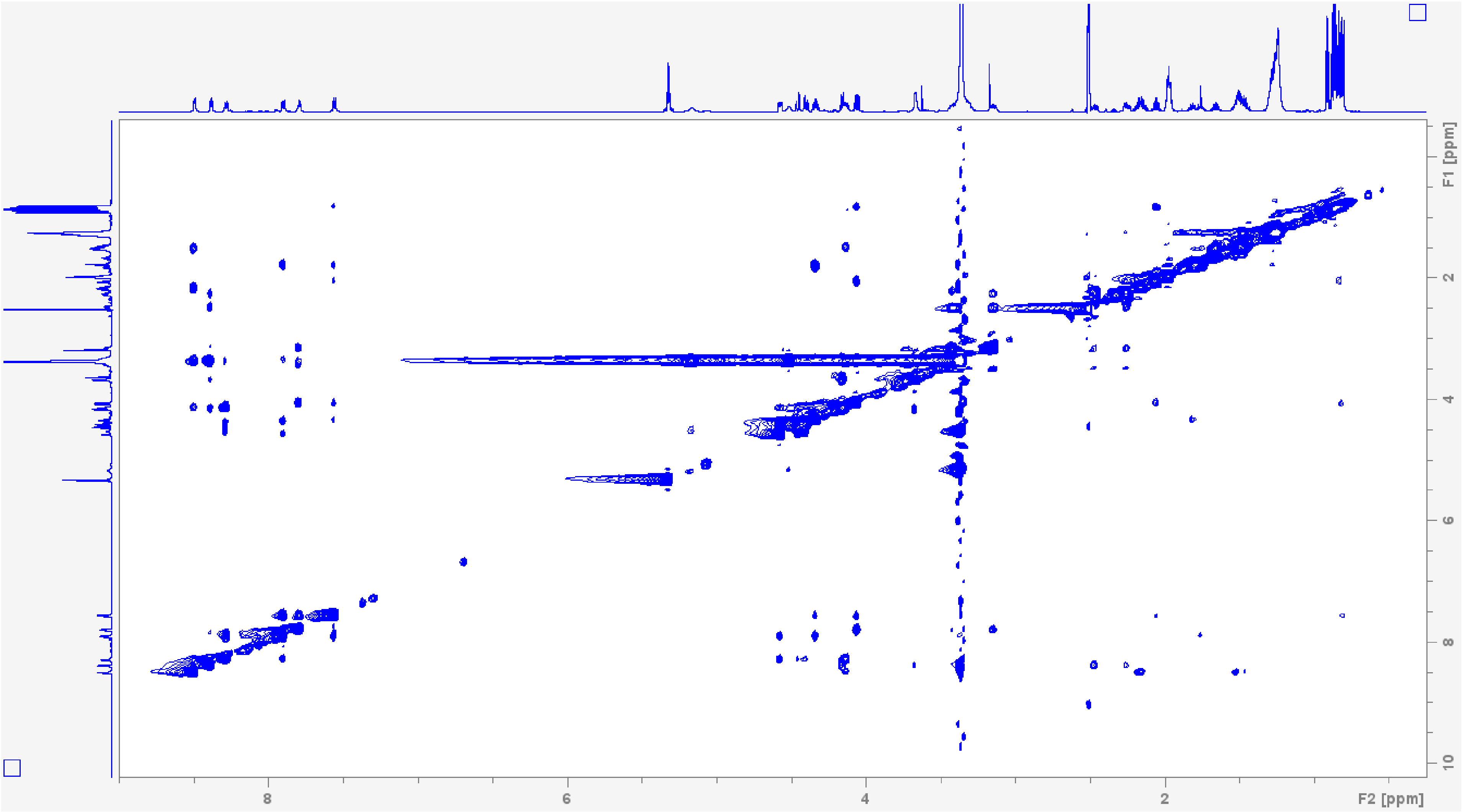

^{1}H NMR spectrum in chloroform-*d* for compound **3**

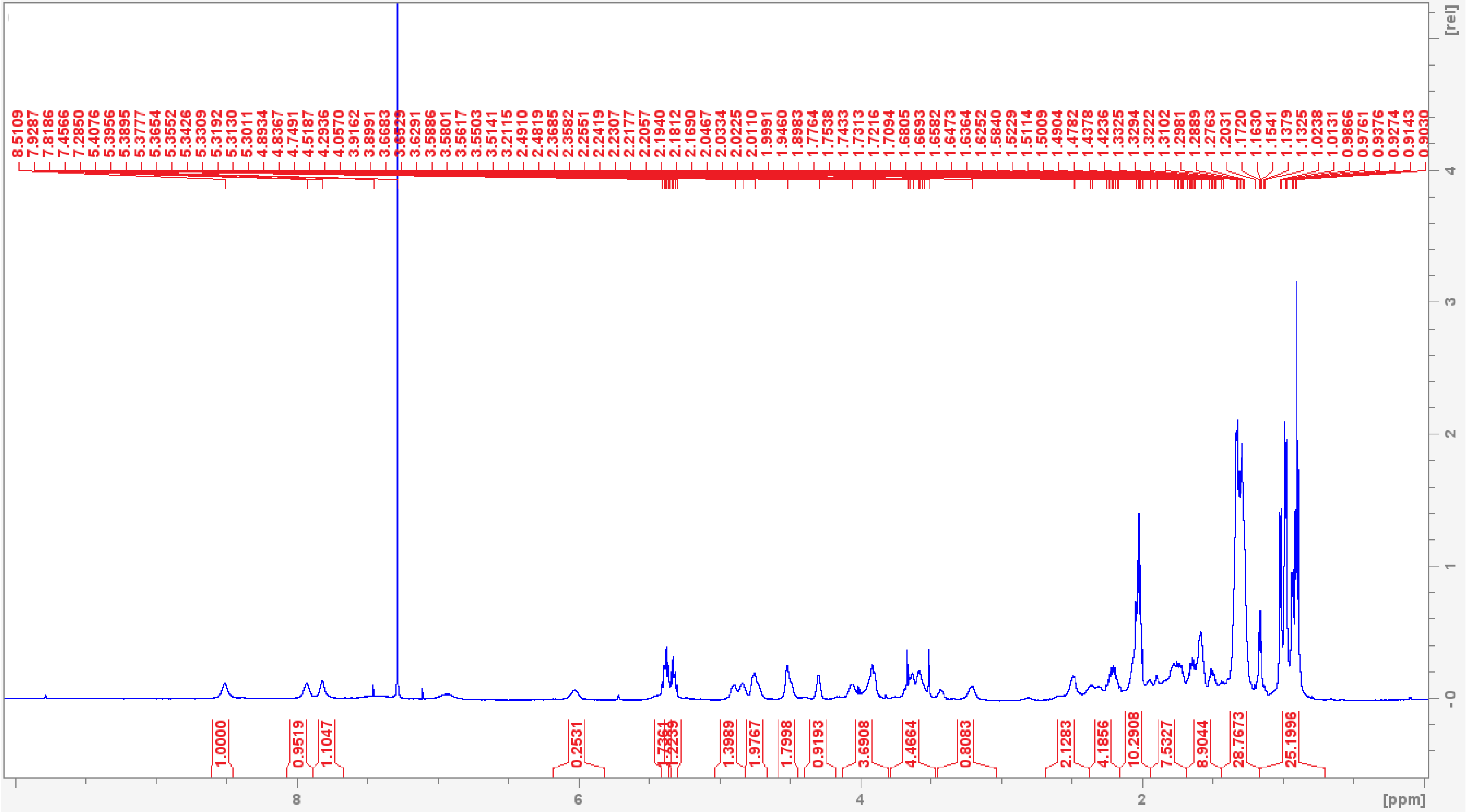

HRMS spectrum for compound **3**

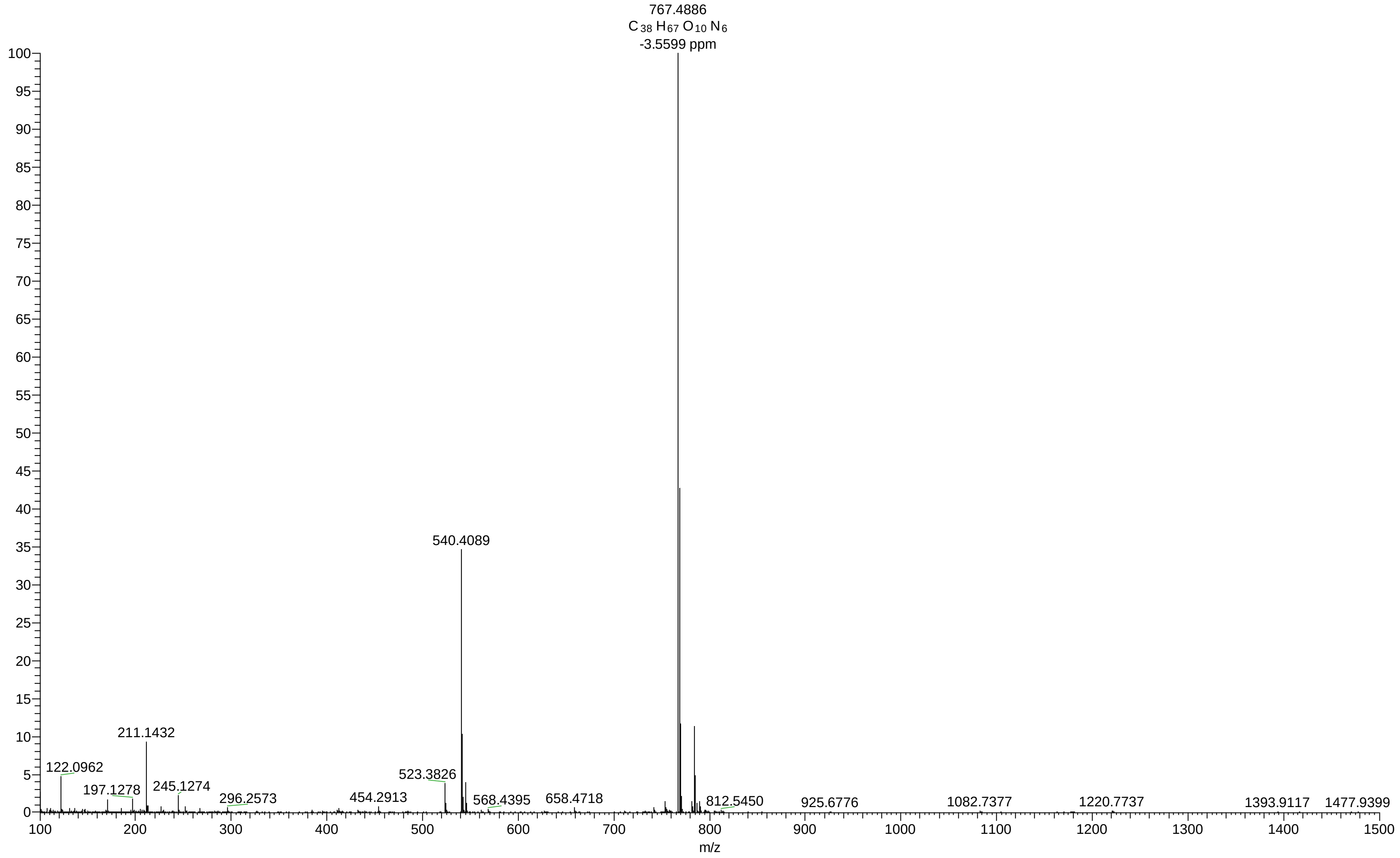

^{1}H NMR spectrum in DMSO-d_6 for compound **4**

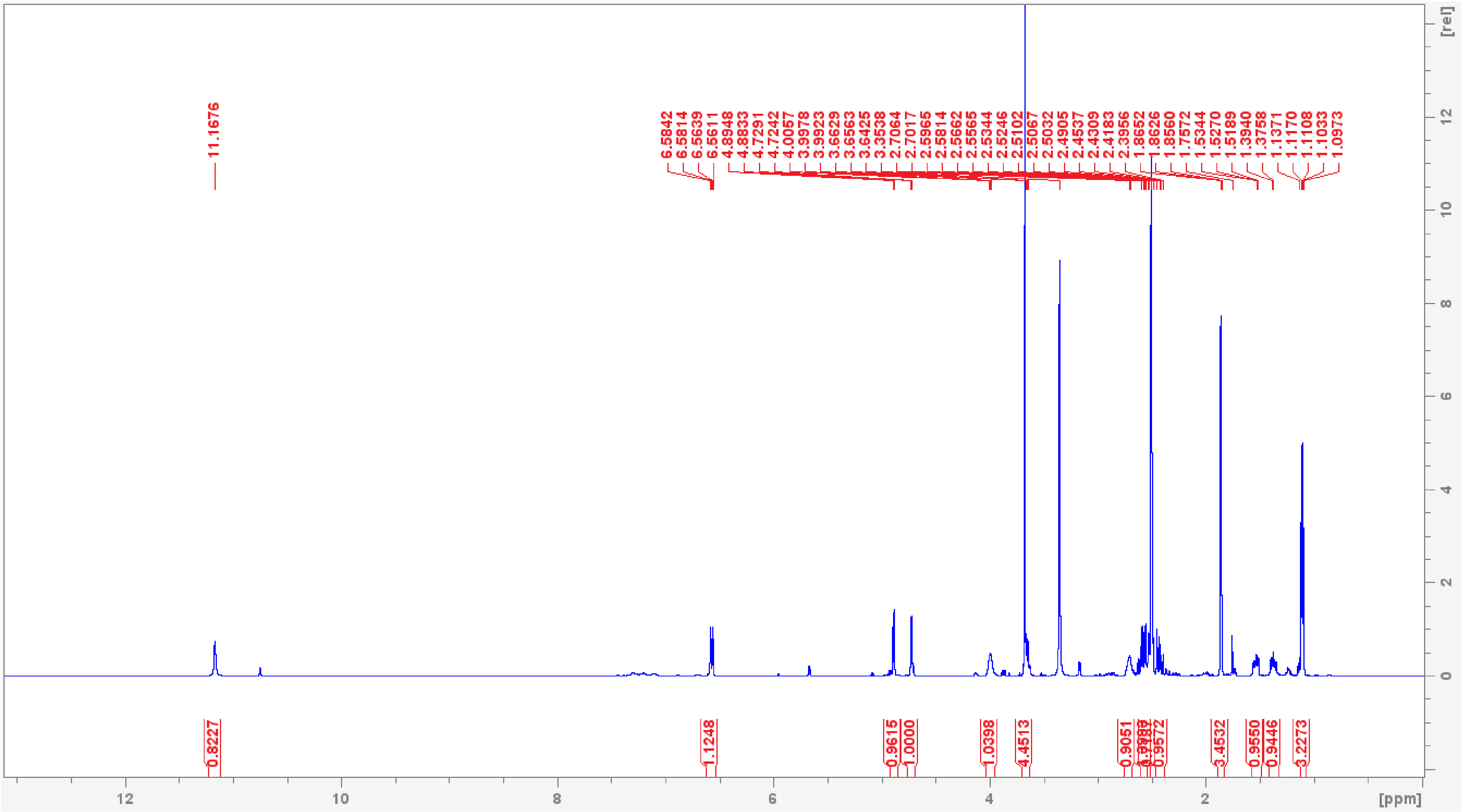

^{13}C NMR spectrum in DMSO-d_6 for compound **4**

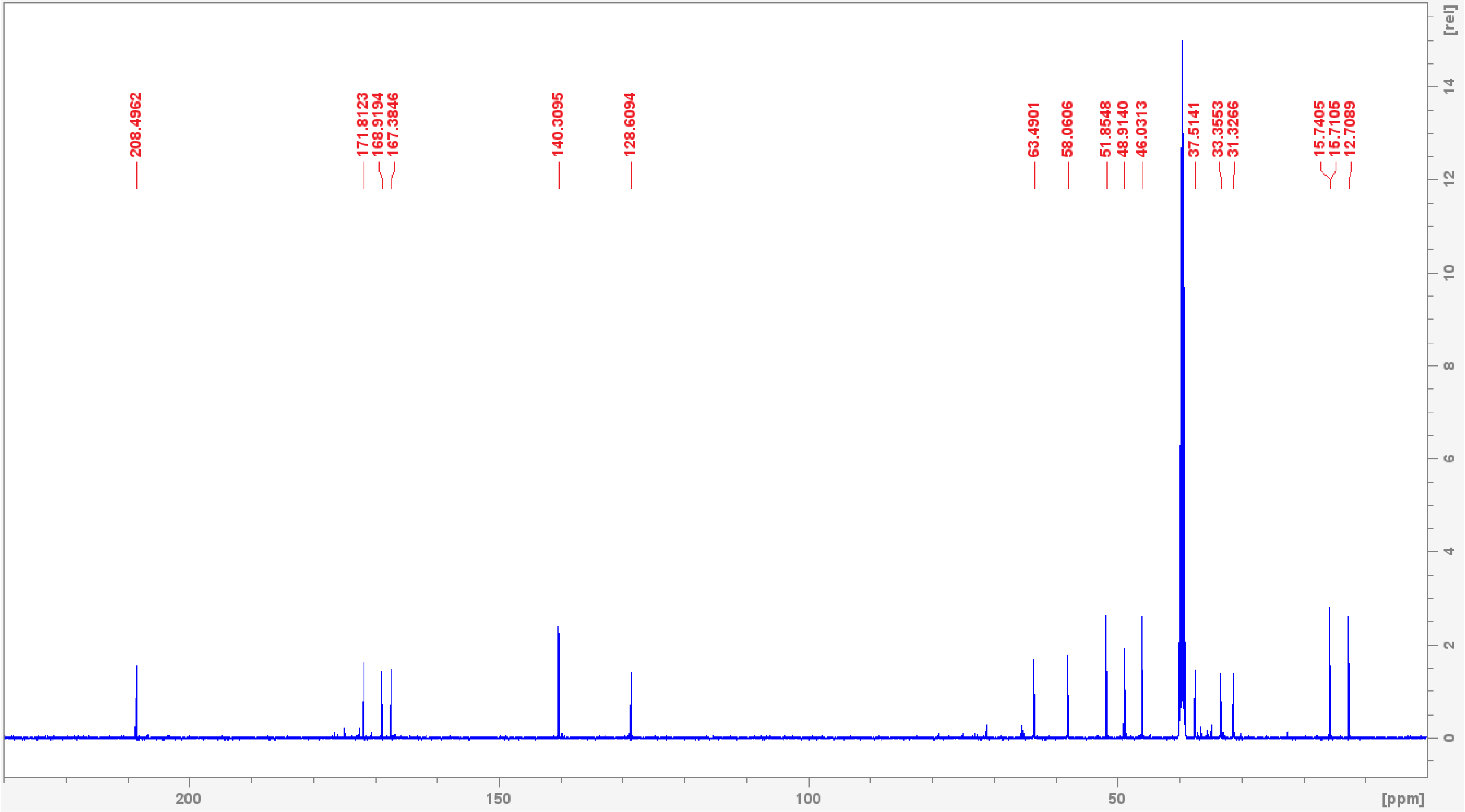

^{1}H-^{1}H COSY spectrum in DMSO-d_6 for compound **4**

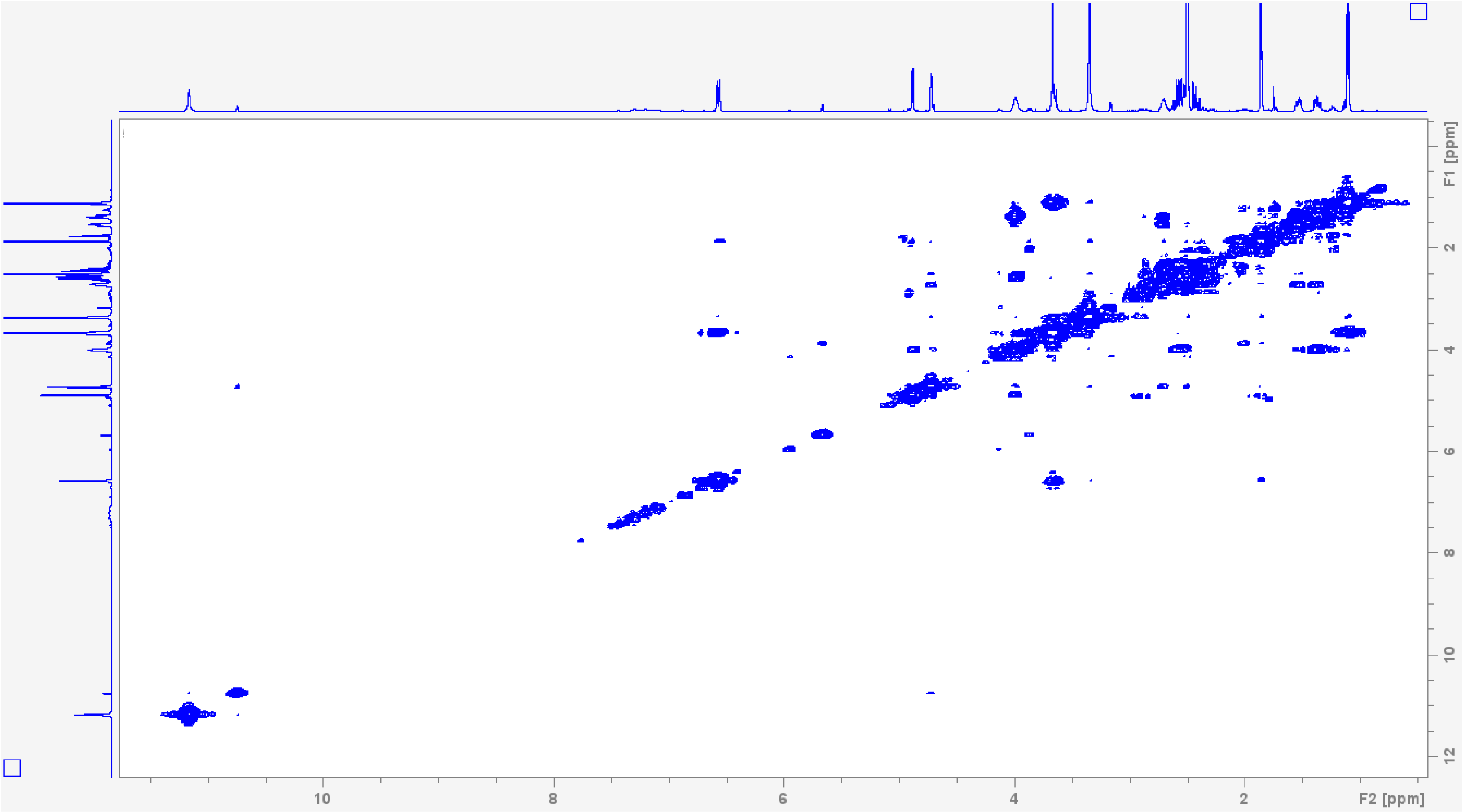

HSQC spectrum in DMSO-d_6 for compound **4**

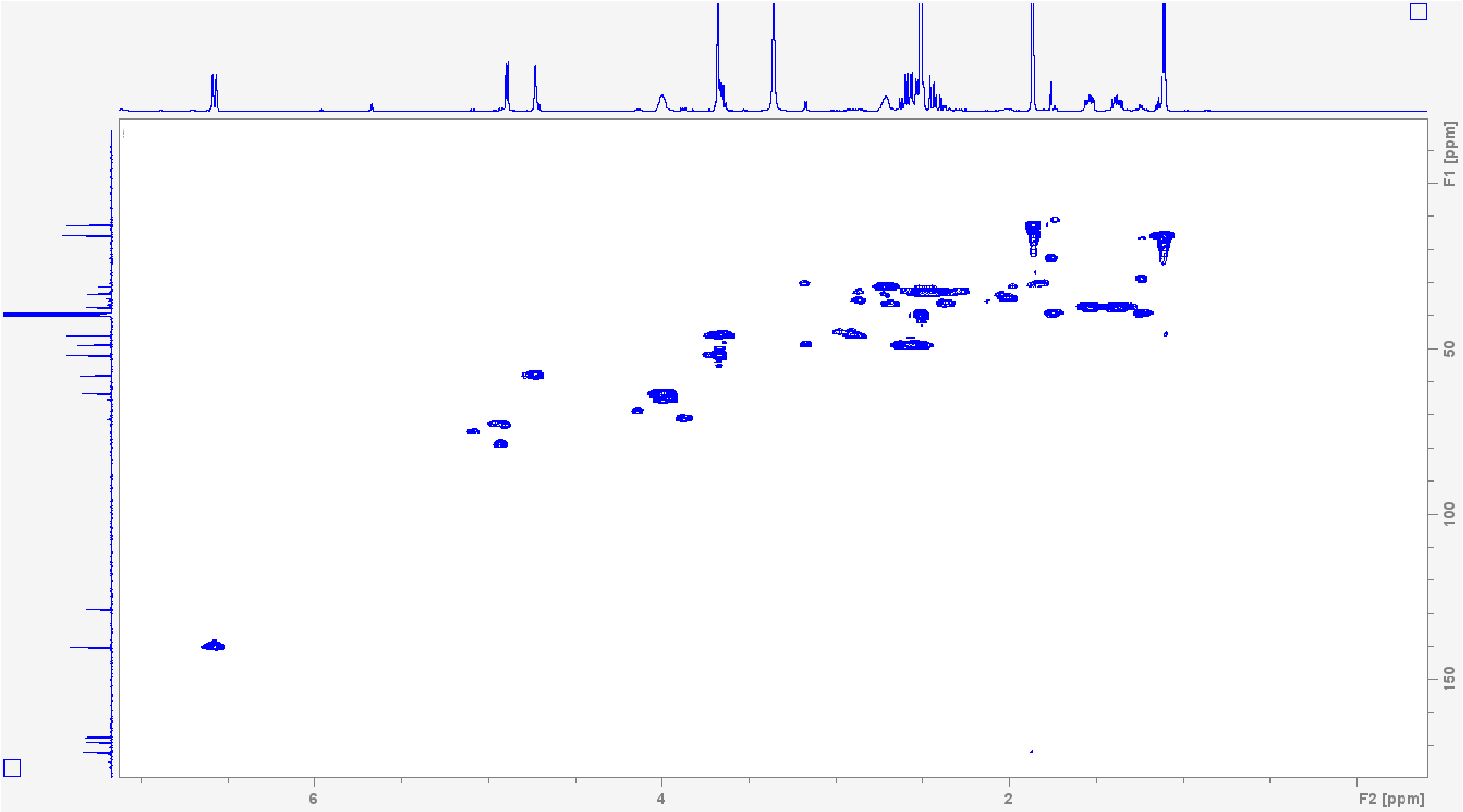

HMBC spectrum in DMSO-d_6 for compound **4**

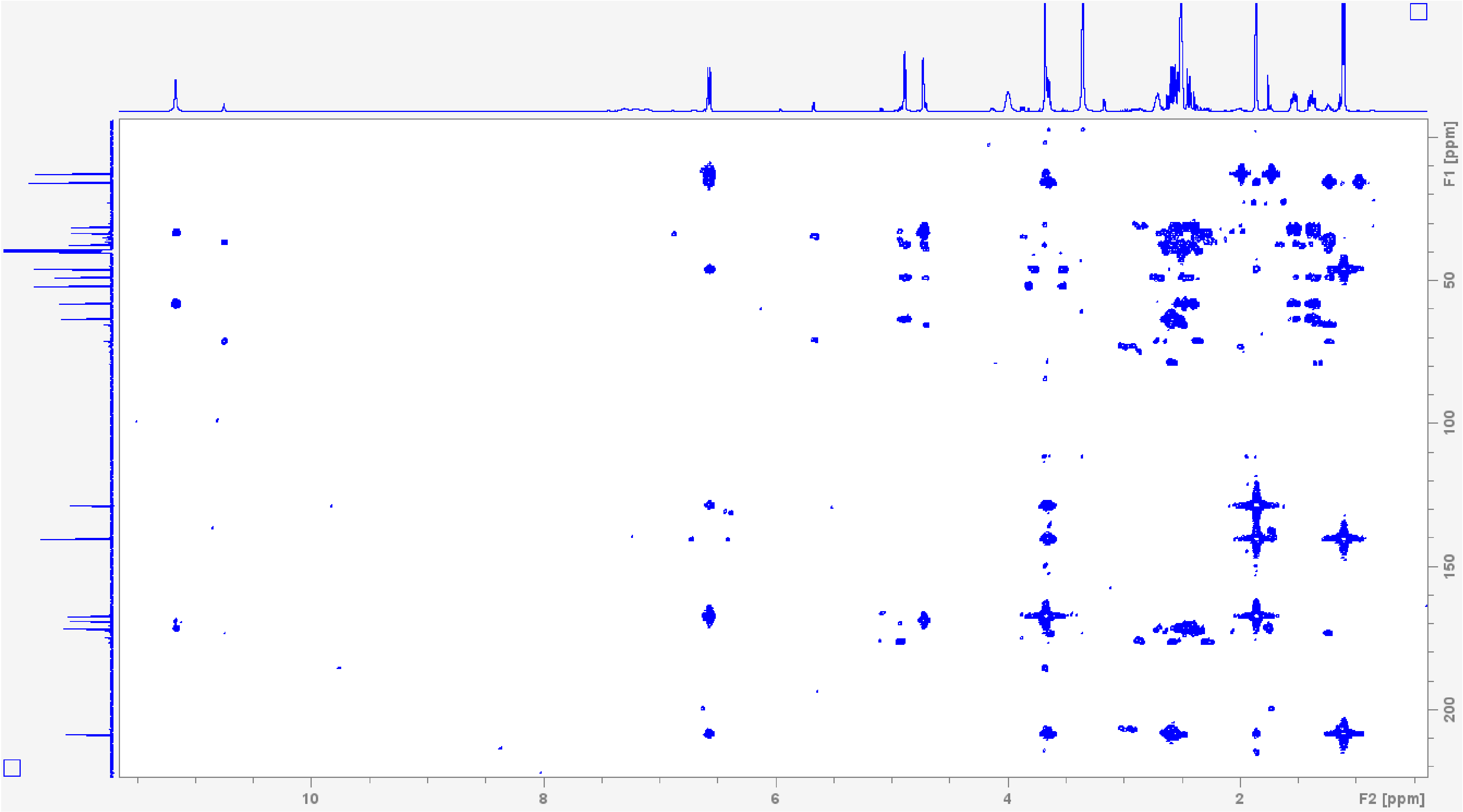

NOESY spectrum in DMSO-d_6 for compound **4**

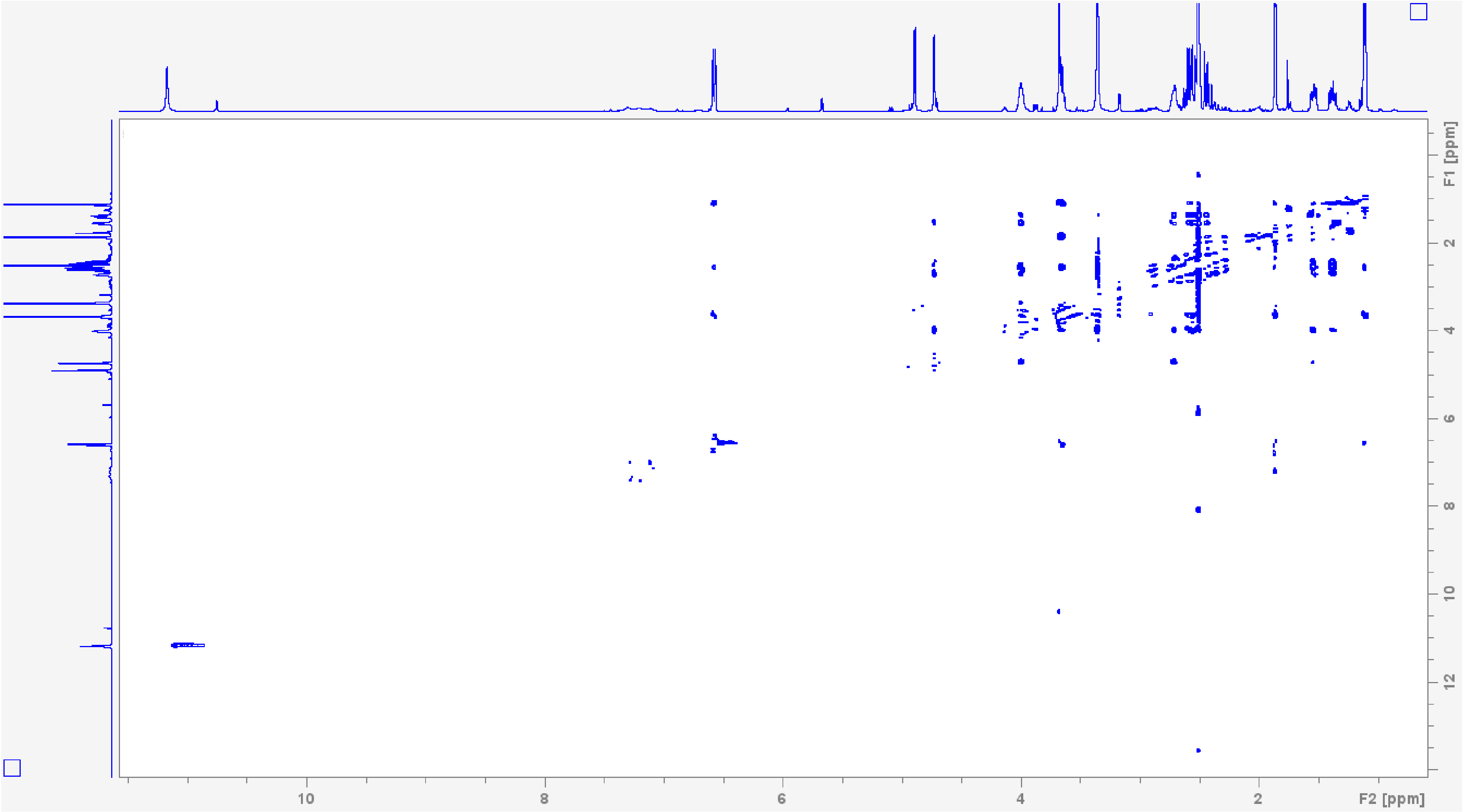

HRMS spectrum for compound **4**

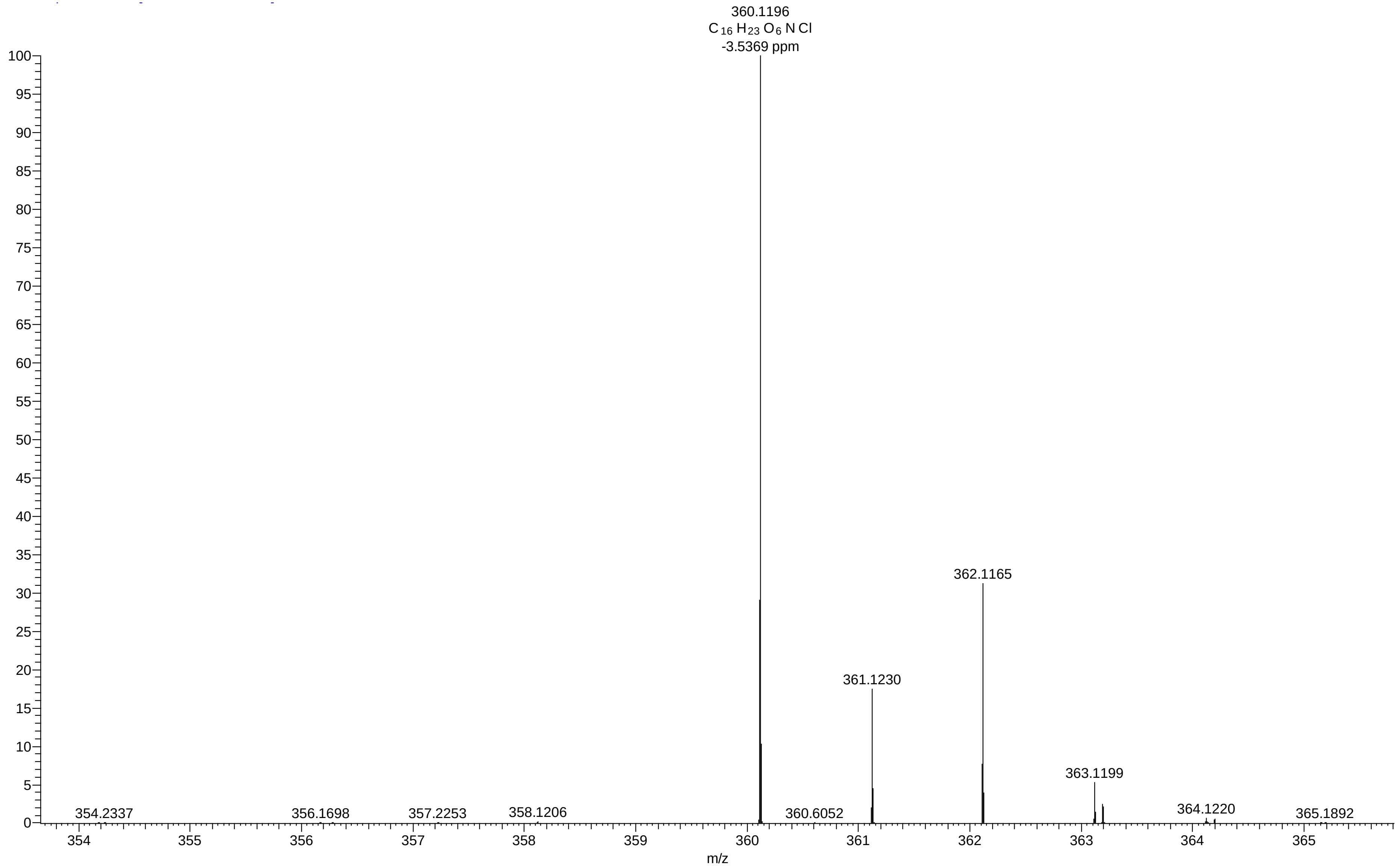

^{1}H NMR spectrum in DMSO-d_6 for compound **5**

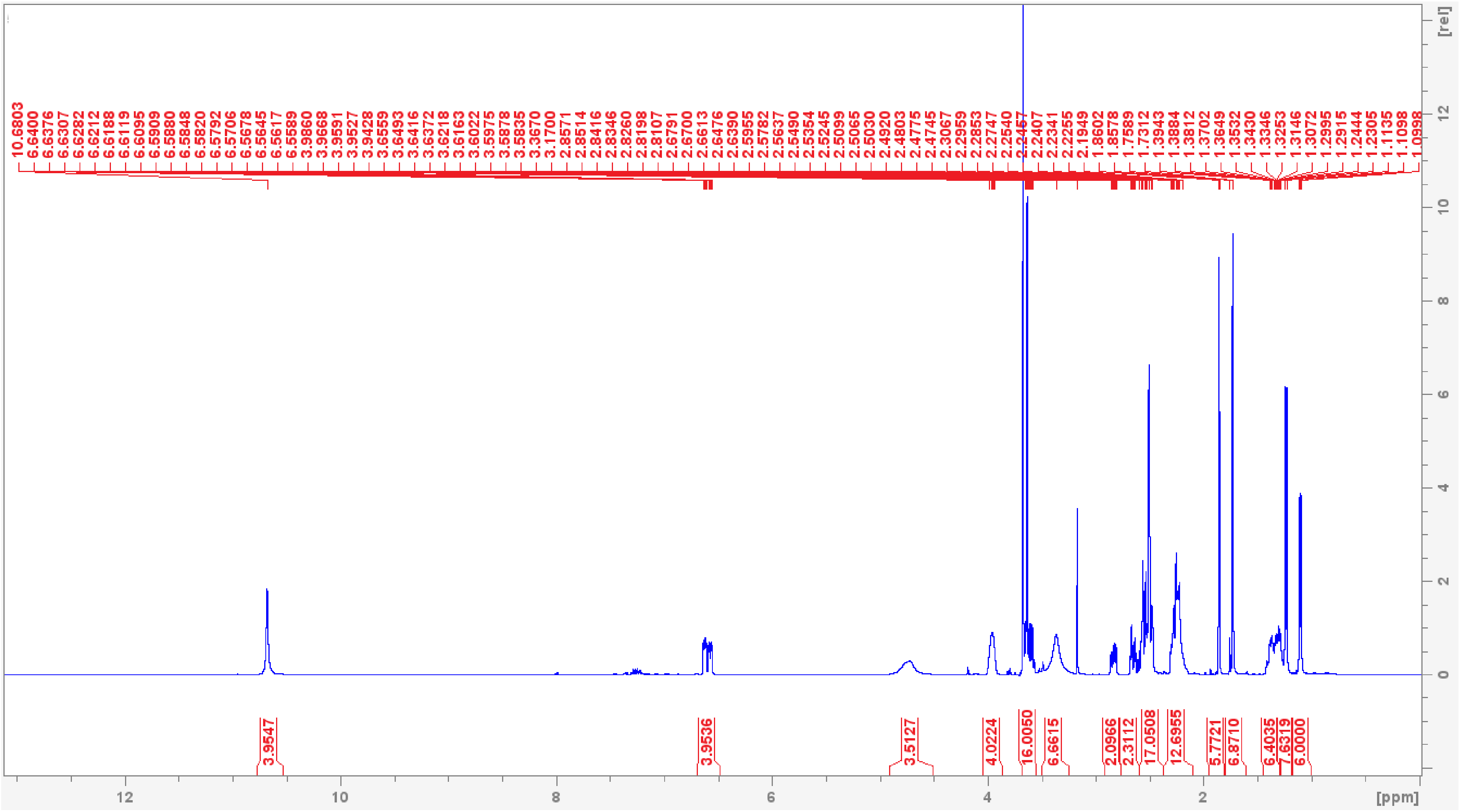

^{13}C NMR spectrum in DMSO-d_6 for compound **5**

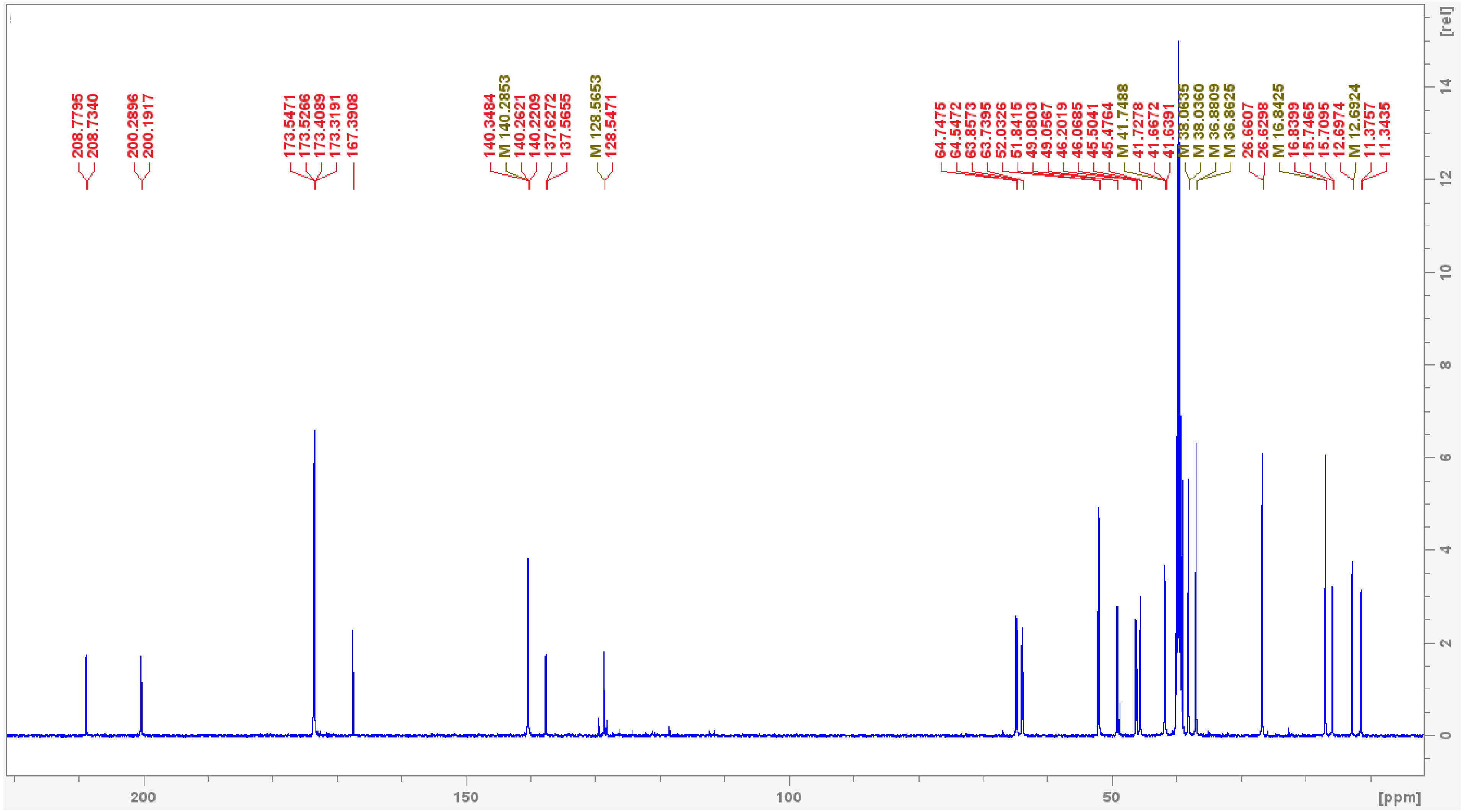

^{1}H-^{1}H COSY spectrum in DMSO-d_6 for compound **5**

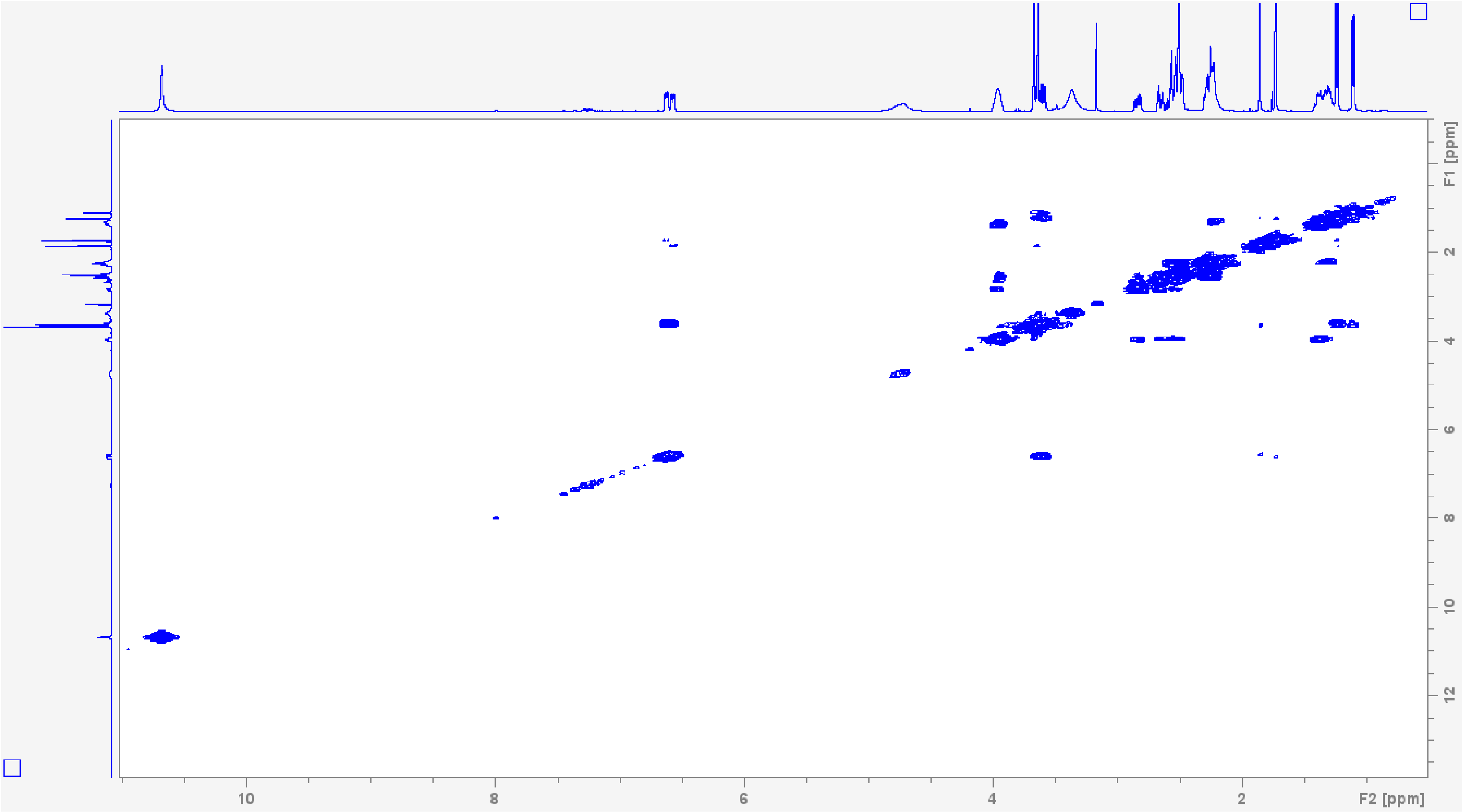

HSQC spectrum in DMSO-d_6 for compound **5**

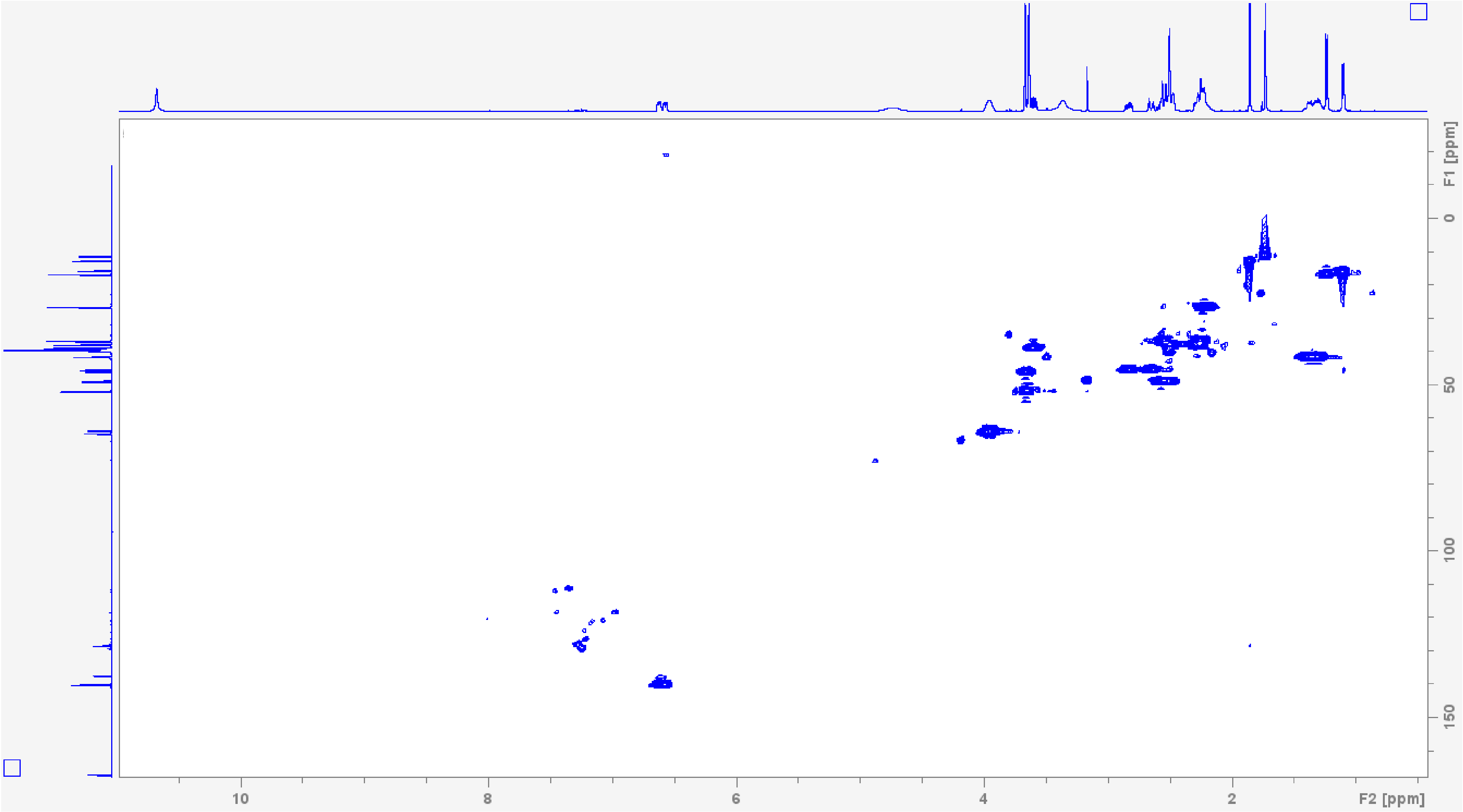

HMBC spectrum in DMSO-d_6 for compound **5**

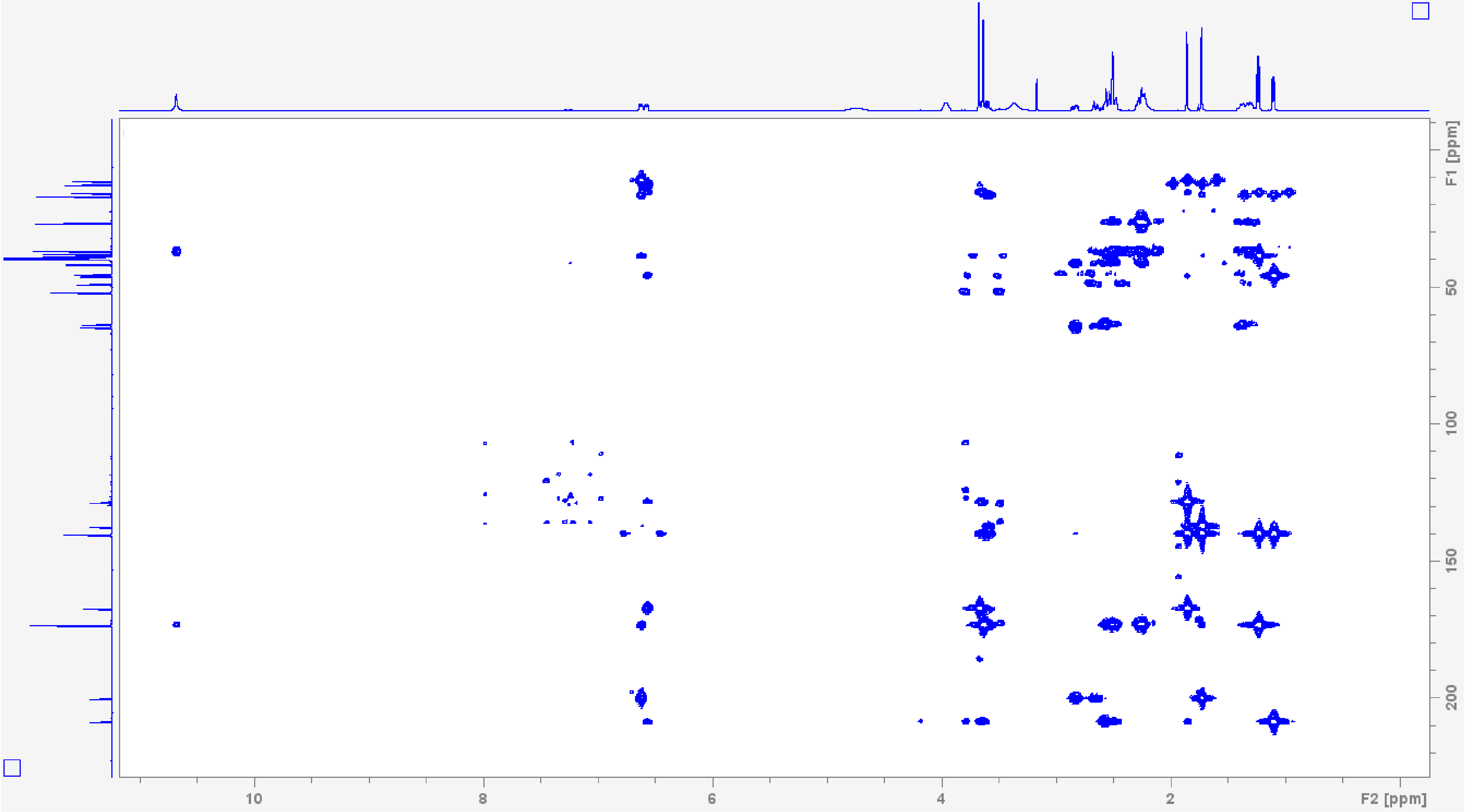

NOESY spectrum in DMSO-d_6 for compound **5**

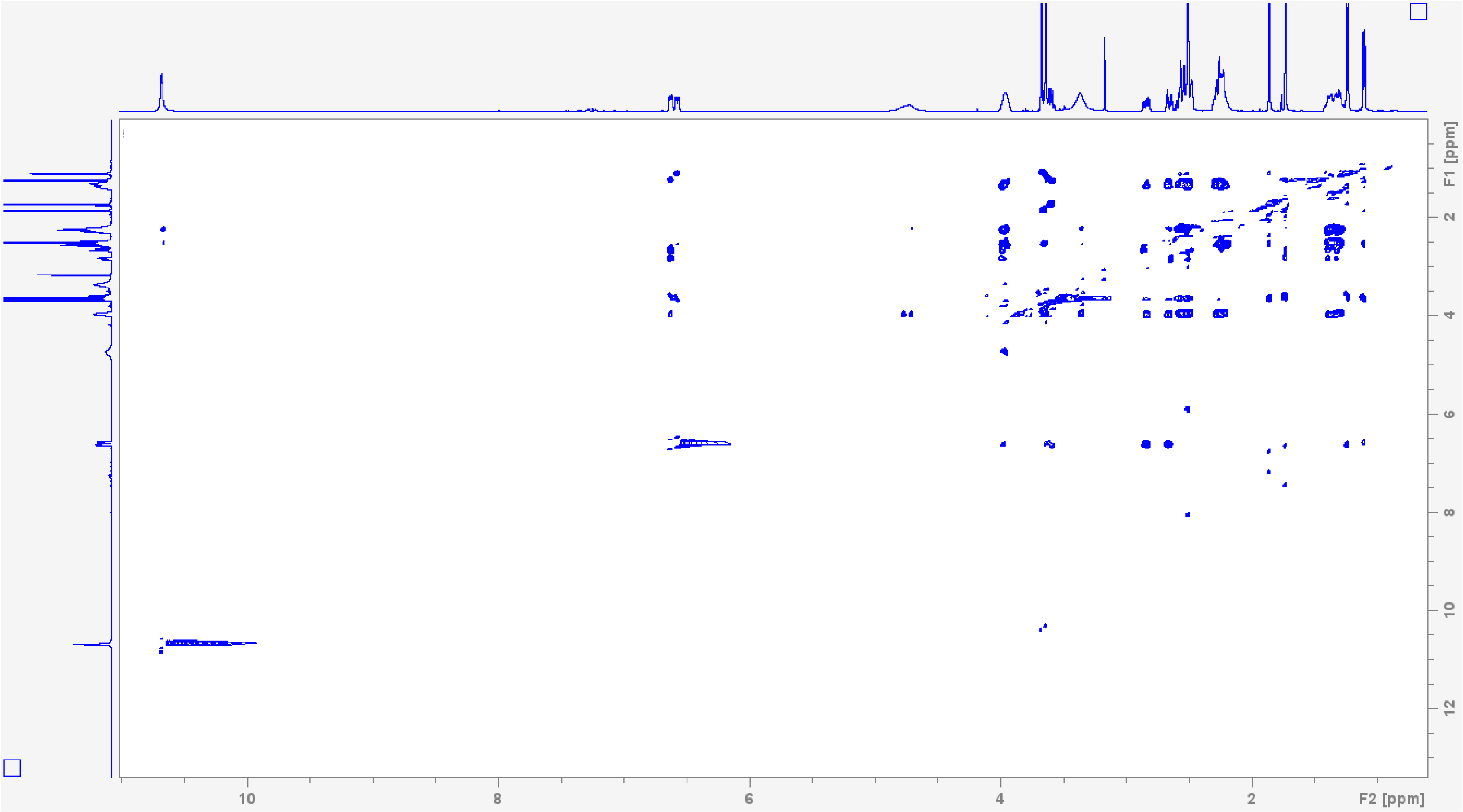

HRMS spectrum for compound **5**

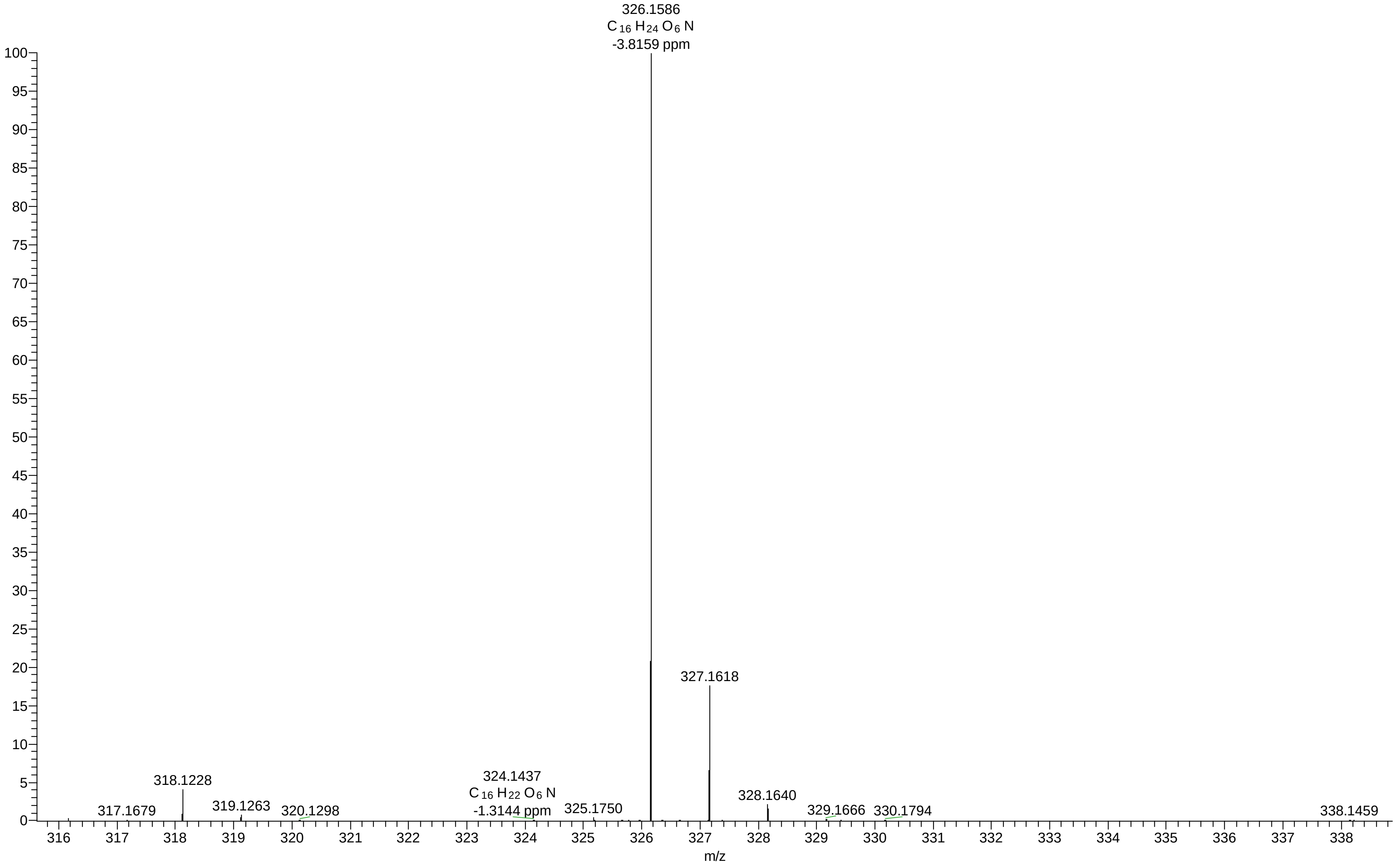

^{1}H NMR spectrum in $CDCl_3$ for compound **5**

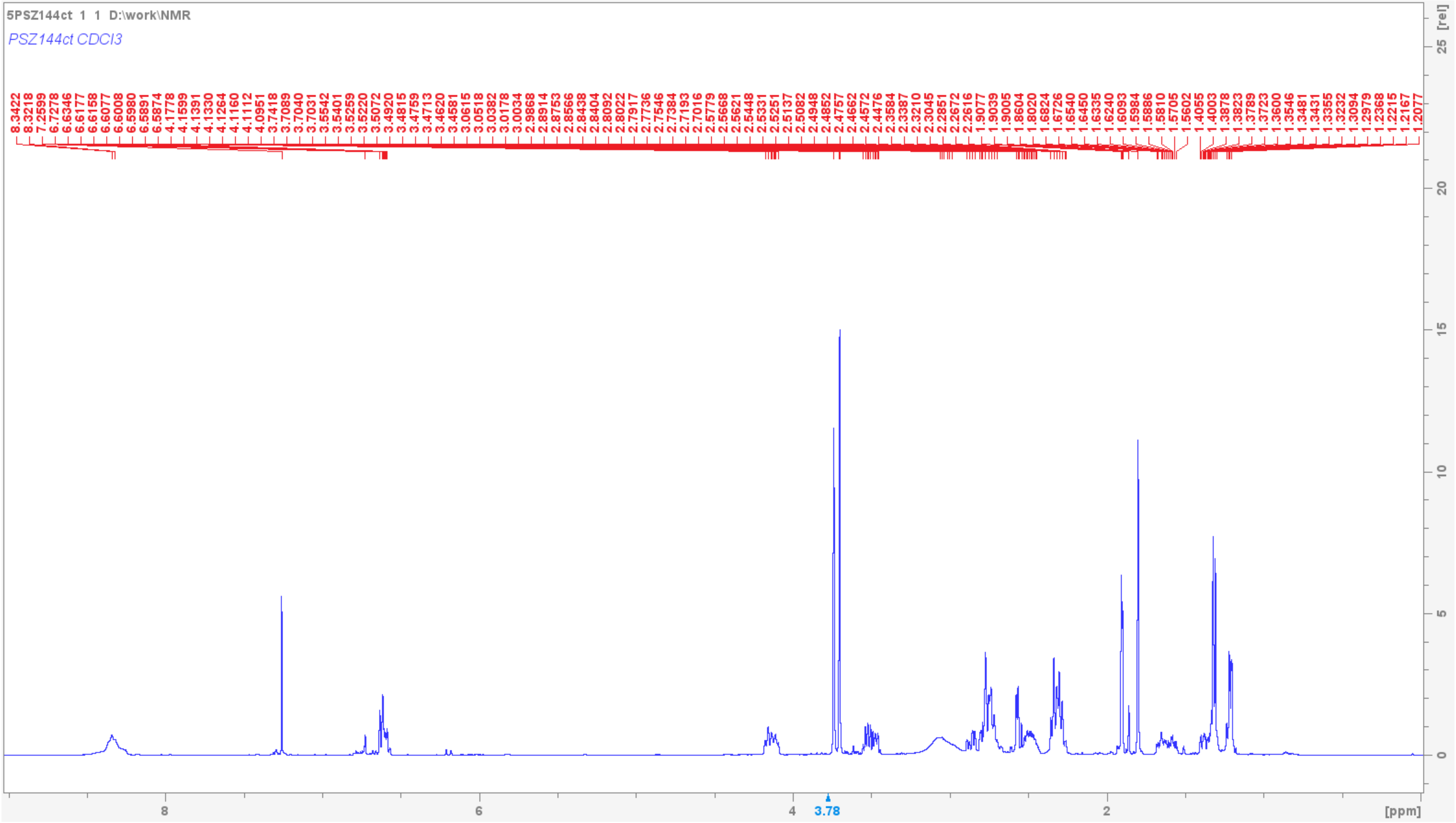

^{13}C NMR spectrum in $CDCl_3$ for compound **5**

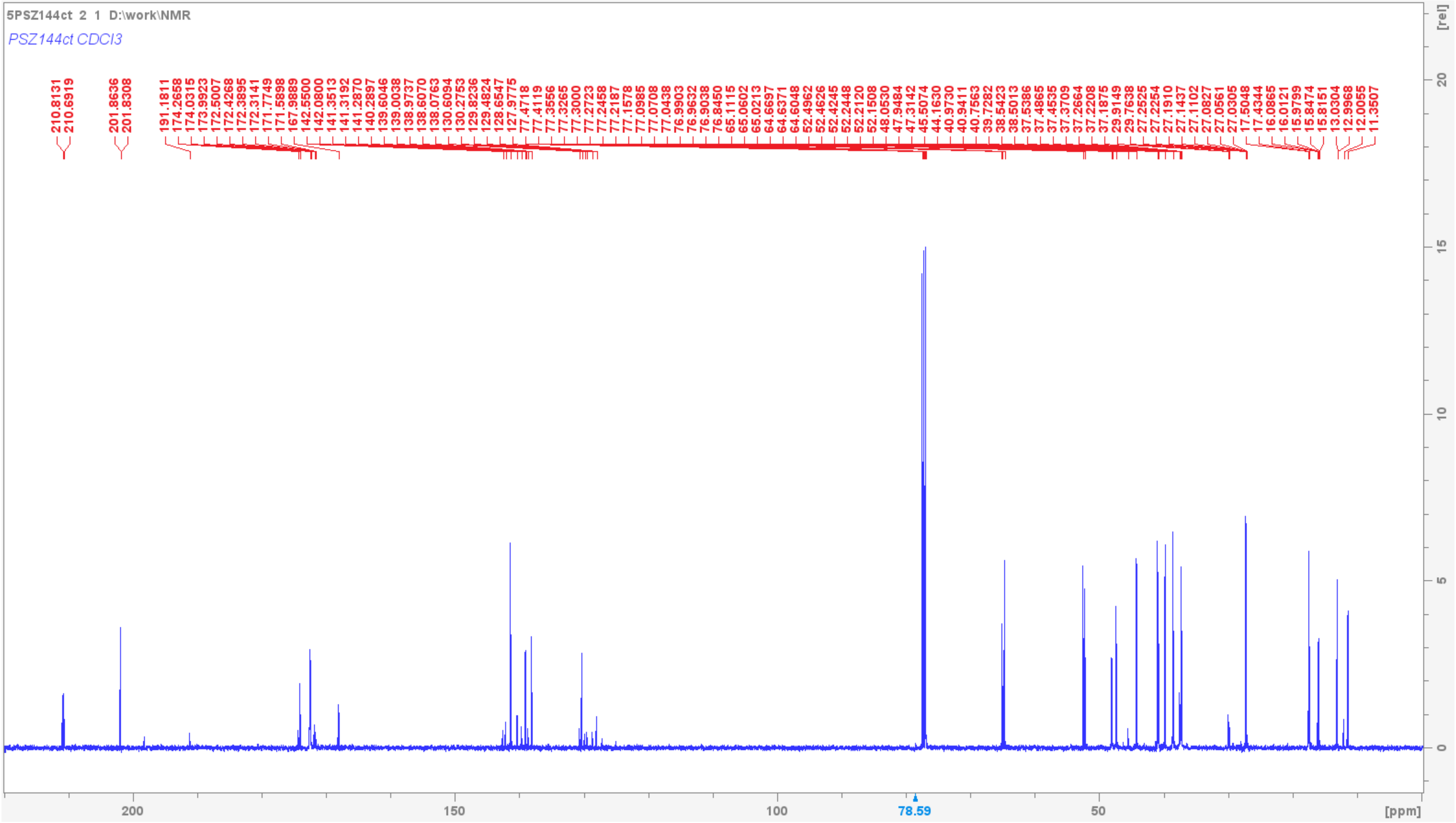

^1H-^1H COSY spectrum in $CDCl_3$ for compound **5**

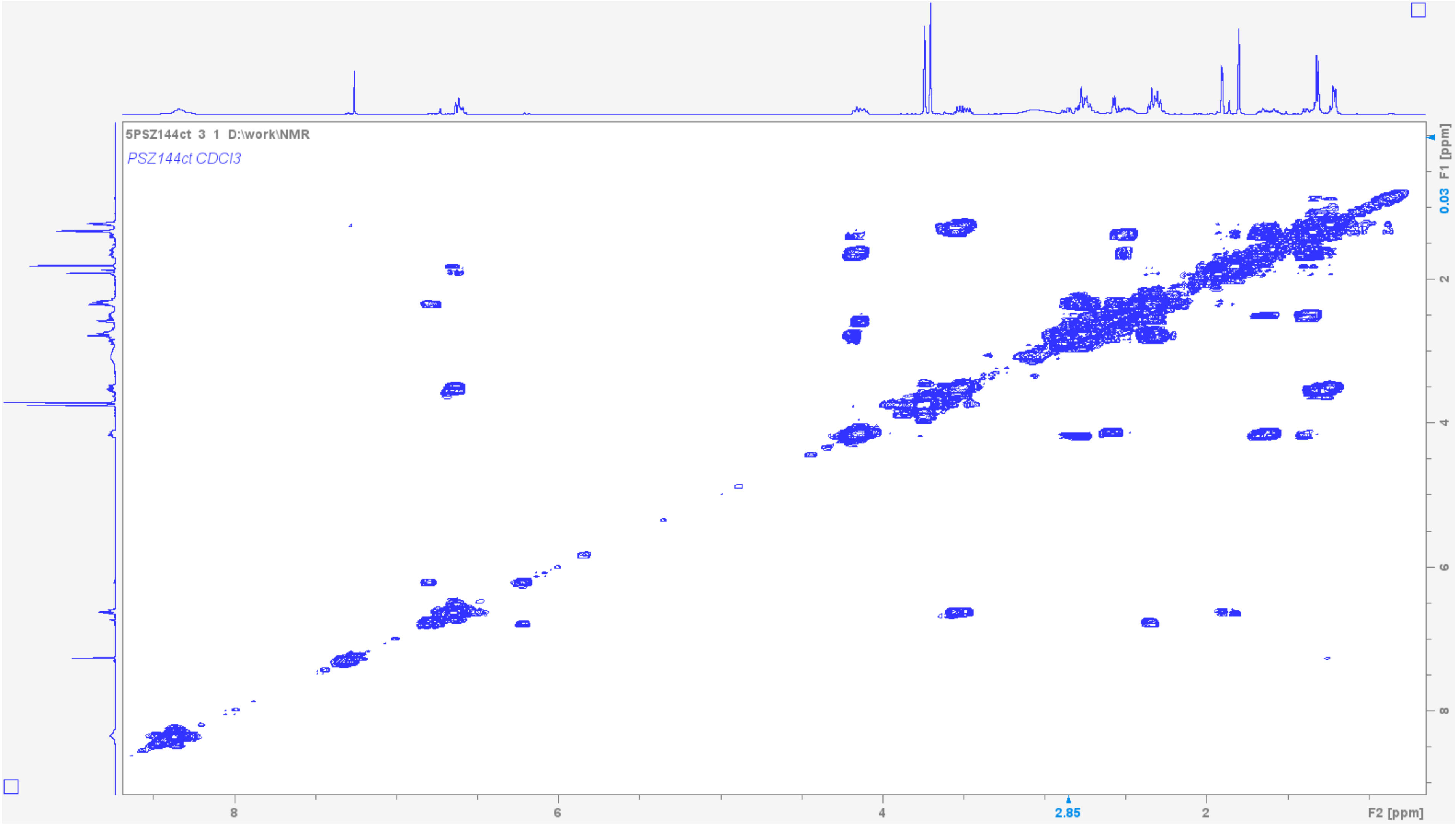

HSQC spectrum in $CDCl_3$ for compound **5**

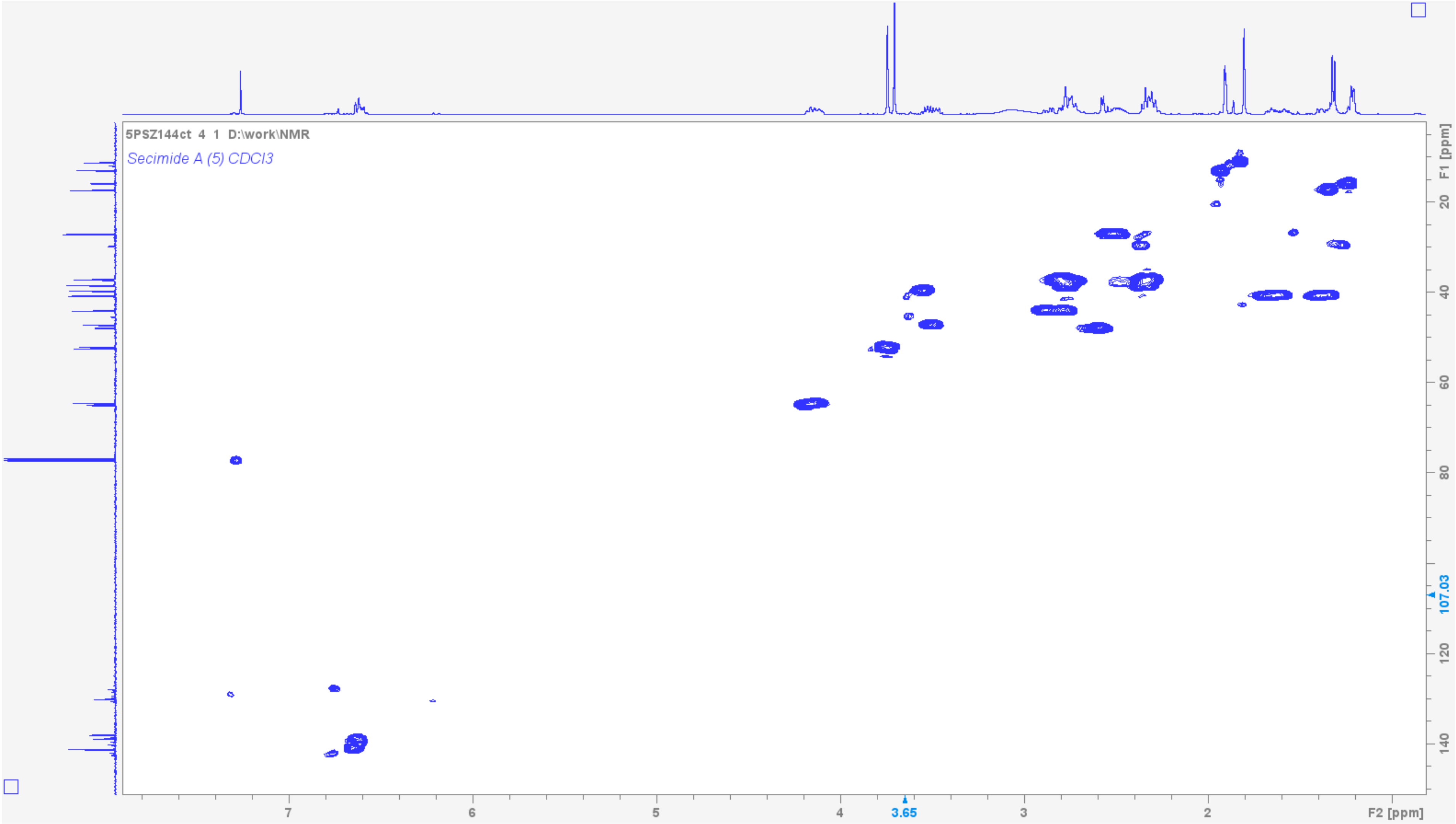

HMBC spectrum in $CDCl_3$ for compound **5**

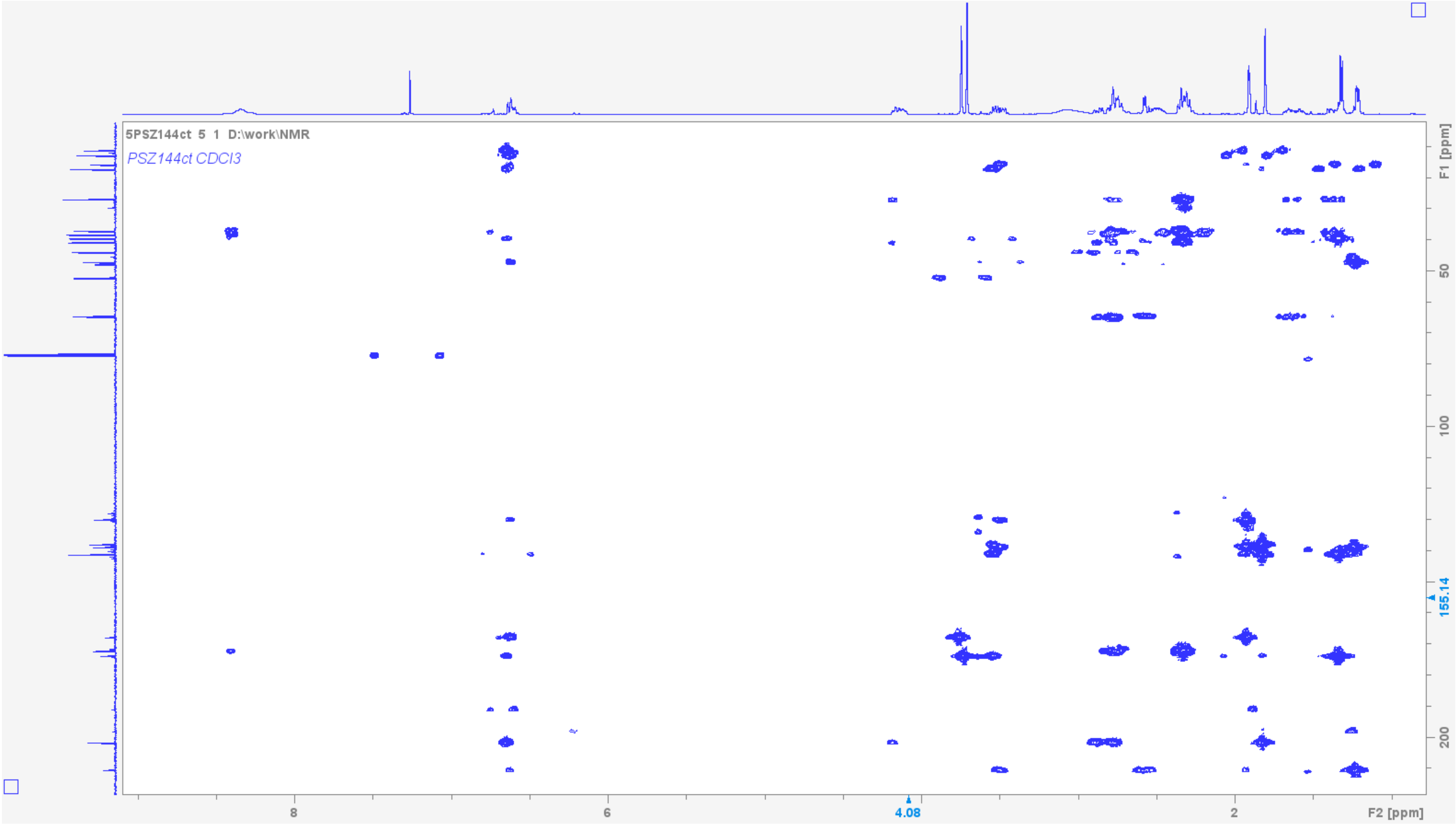

NOESY spectrum in $CDCl_3$ for compound **5**

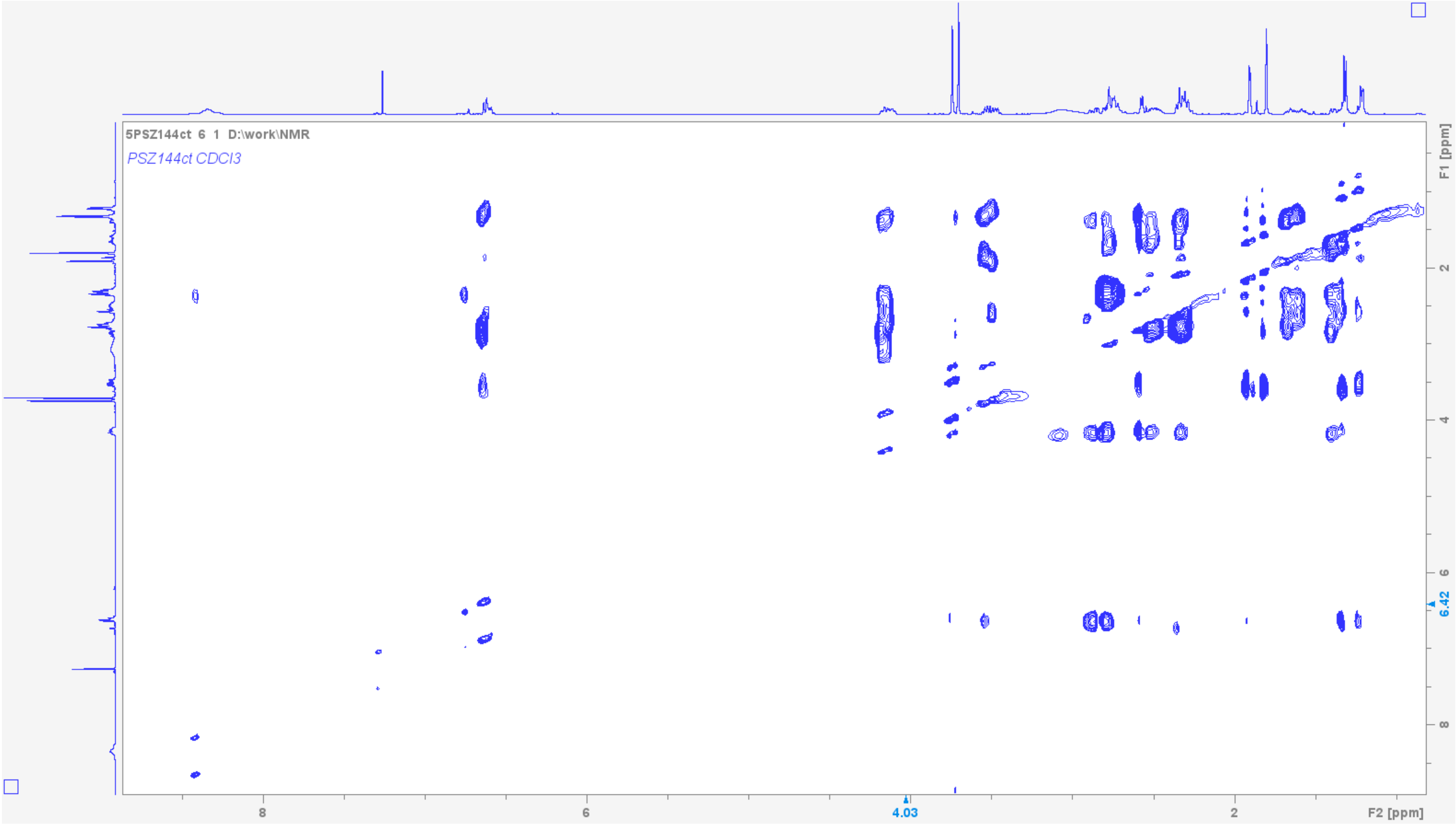

^{1}H NMR spectrum in DMSO-d_6 for compound **5d**

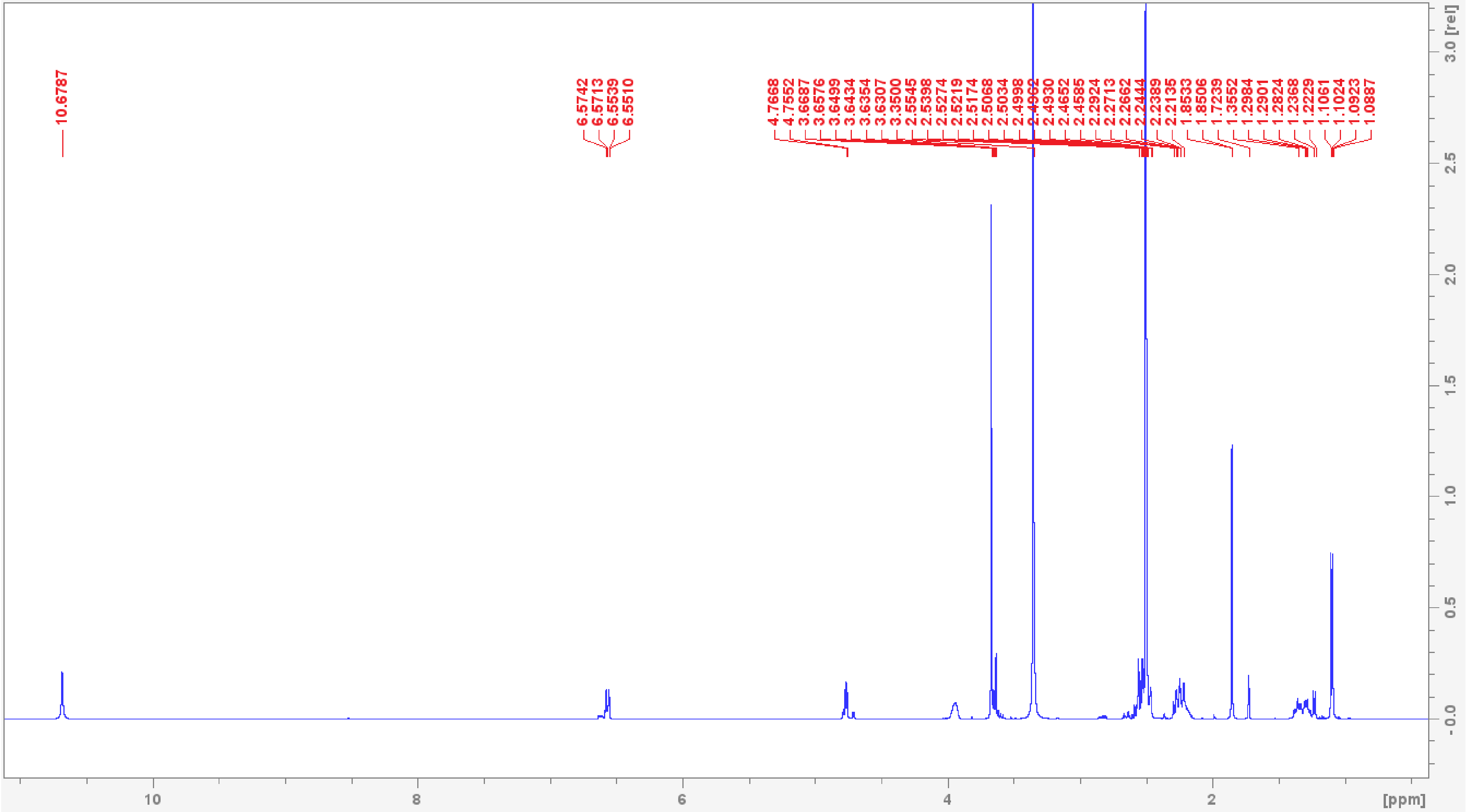

Comparison of ^{1}H NMR spectrum in DMSO-d_6 between mixture of **5a-5d** and **5d**

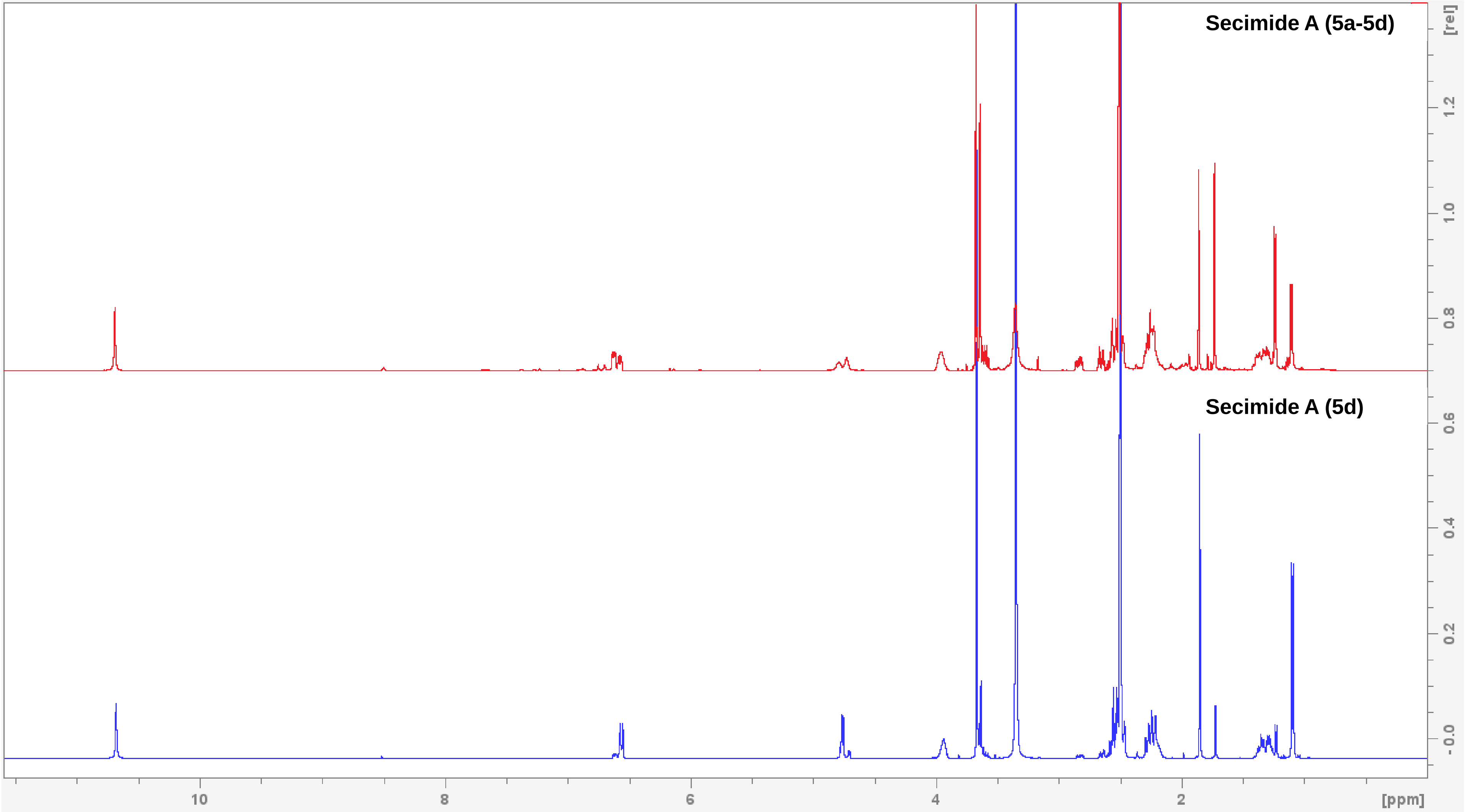

HSQC spectrum in DMSO-d_6 for compound **5d**

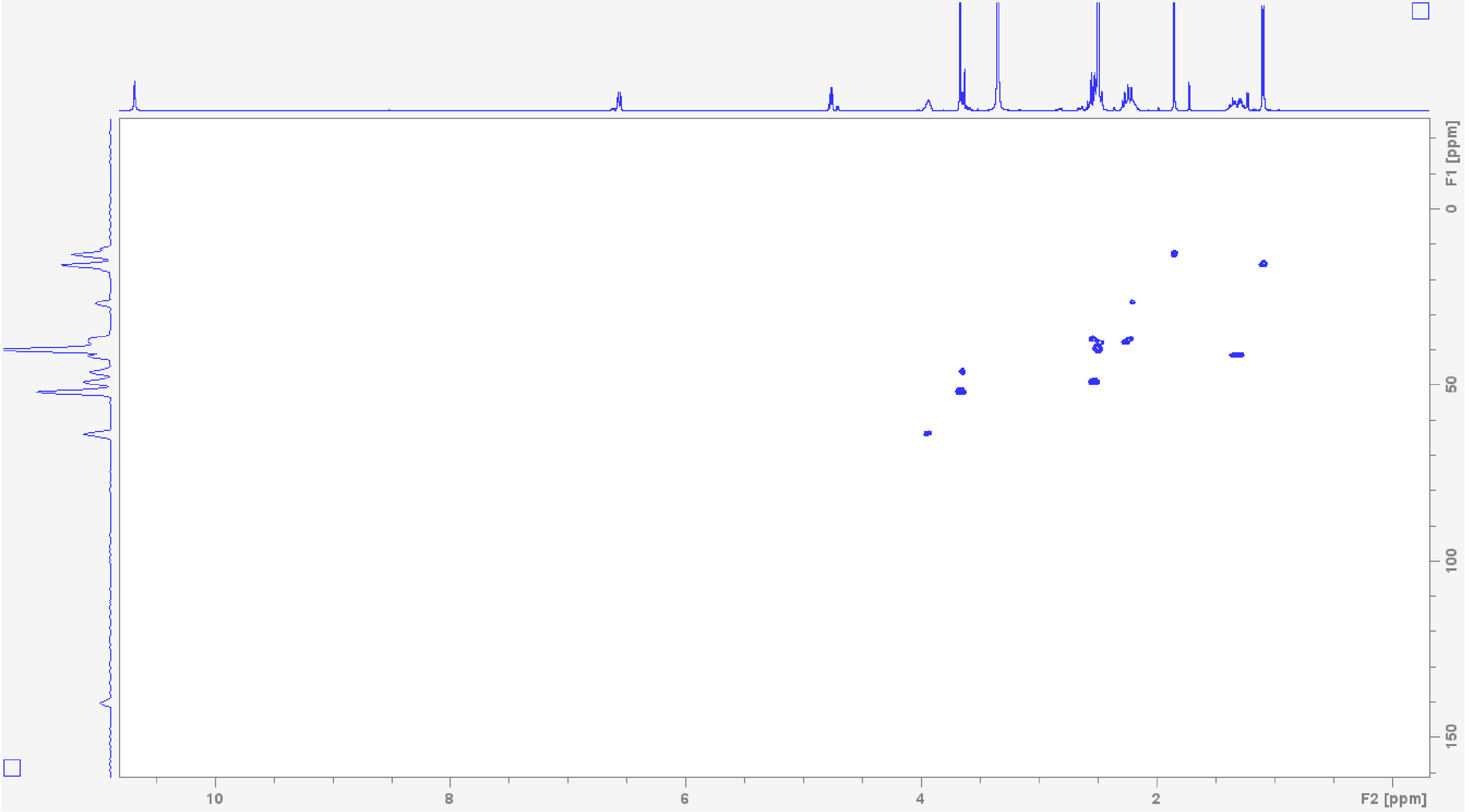

Comparison of HSQC spectrum in DMSO-d_6 between mixture of **5a**-**5d** and **5d** (Red: mixture of **5a**-**5d**; blue: **5d**)

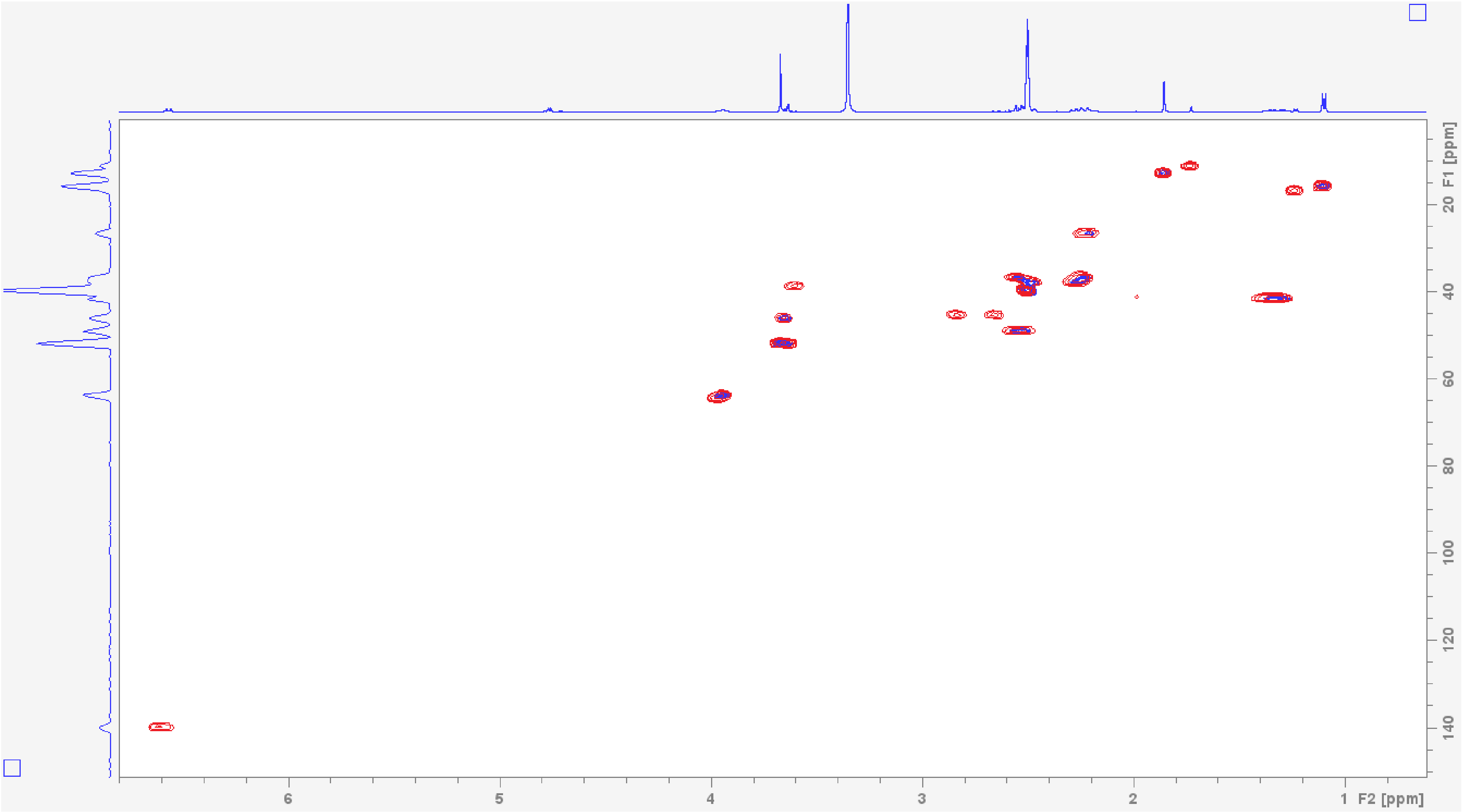

Supplement: Supplementary file 1 — Supporting Information [file ANIE-64-e202503679-s002.pdf]
